# Treating major depression with yoga: A prospective, randomized, controlled pilot trial

**DOI:** 10.1371/journal.pone.0173869

**Published:** 2017-03-16

**Authors:** Sudha Prathikanti, Renee Rivera, Ashly Cochran, Jose Gabriel Tungol, Nima Fayazmanesh, Eva Weinmann

**Affiliations:** 1 Department of Psychiatry, School of Medicine, University of California San Francisco, San Francisco, California, United States of America; 2 Osher Center for Integrative Medicine, University of California San Francisco, San Francisco, California, United States of America; 3 Department of Psychiatry, Weill Cornell Medicine, Cornell University, New York, New York, United States of America; 4 Johns Hopkins Bloomberg School of Public Health, Johns Hopkins University, Baltimore, Maryland, United States of America; 5 Department of Psychiatry, Veterans Affairs Long Beach Healthcare System, Long Beach, California, United States of America; 6 Fuss Ueber Kopf Yoga Studio, Svastha Yoga Therapy Program, Stuttgart, Germany; Pondicherry Institute of Medical Sciences, INDIA

## Abstract

**Background:**

Conventional pharmacotherapies and psychotherapies for major depression are associated with limited adherence to care and relatively low remission rates. Yoga may offer an alternative treatment option, but rigorous studies are few. This randomized controlled trial with blinded outcome assessors examined an 8-week hatha yoga intervention as mono-therapy for mild-to-moderate major depression.

**Methods:**

Investigators recruited 38 adults in San Francisco meeting criteria for major depression of mild-to-moderate severity, per structured psychiatric interview and scores of 14–28 on Beck Depression Inventory-II (BDI). At screening, individuals engaged in psychotherapy, antidepressant pharmacotherapy, herbal or nutraceutical mood therapies, or mind-body practices were excluded. Participants were 68% female, with mean age 43.4 years (SD = 14.8, range = 22–72), and mean BDI score 22.4 (SD = 4.5). Twenty participants were randomized to 90-minute hatha yoga practice groups twice weekly for 8 weeks. Eighteen participants were randomized to 90-minute attention control education groups twice weekly for 8 weeks. Certified yoga instructors delivered both interventions at a university clinic. Primary outcome was depression severity, measured by BDI scores every 2 weeks from baseline to 8 weeks. Secondary outcomes were self-efficacy and self-esteem, measured by scores on the General Self-Efficacy Scale (GSES) and Rosenberg Self-Esteem Scale (RSES) at baseline and at 8 weeks.

**Results:**

In intent-to-treat analysis, yoga participants exhibited significantly greater 8-week decline in BDI scores than controls (p-value = 0.034). In sub-analyses of participants completing final 8-week measures, yoga participants were more likely to achieve remission, defined per final BDI score ≤ 9 (p-value = 0.018). Effect size of yoga in reducing BDI scores was large, per Cohen’s d = -0.96 [95%CI, -1.81 to -0.12]. Intervention groups did not differ significantly in 8-week change scores for either the GSES or RSES.

**Conclusion:**

In adults with mild-to-moderate major depression, an 8-week hatha yoga intervention resulted in statistically and clinically significant reductions in depression severity.

**Trial registration:**

ClinicalTrials.gov NCT01210651

## Introduction

### Major depression and its impact

Annually, nearly 7% of adults in the Unites States suffer from an episode of major depression [1]. The World Health Organization identifies major depression as the second most disabling medical condition in the United States, accounting for more years lived with disability than heart disease, stroke, or diabetes [2]. Major depression contributes not only to disability, but also to mortality. There are more than 49,000 deaths by suicide annually in the United States [3], and it is estimated that major depression accounts for 20–35% of these deaths [4]. Major depression also increases the risk of death from other medical conditions such as coronary artery disease [5–8] and diabetes mellitus [9–11].

According to current diagnostic criteria [12], major depression is characterized by five or more of the following symptoms being present concurrently for at least two weeks: 1) depressed mood, 2) loss of interest in previously pleasurable activities, 3) feelings of inappropriate guilt or worthlessness, 4) recurrent thoughts of death or suicide, 5) psychomotor slowing or agitation, 6) disturbance of appetite, 7) disturbance of sleep, 8) disturbance of energy, and 9) impaired concentration. One of the five symptoms must include depressed mood or diminished interest in previously pleasurable activities. A diagnosis of major depression may be further categorized as mild, moderate or severe, based on the level of functional impairment. In severe major depression, suicidal ideation or psychotic symptoms such as hallucinations and delusions may be present. Diagnostic criteria for major depression require it to be distinguished from normal bereavement, as well as from other depressive disorders that may present with low mood, such as dysthymia, cyclothymia, bipolar disorder, premenstrual dysphoria, or affective symptoms that are the manifestation of co-morbid physical illness or substance misuse. Left untreated, an episode of major depression typically lasts about 6 to 12 months, but 20% of episodes may last 2 years or longer [13]. Even after treatment and recovery, more than half of individuals who suffer one episode of major depression have a recurrent episode within a few years. The lengthy duration of each episode, and the tendency for episodes to recur, both contribute to the high level of disability associated with major depression. In this paper, the term “depression” may refer to low mood of any etiology, but “major depression” will refer only to the specific mood illness meeting diagnostic criteria outlined above.

### Conventional care for major depression

Conventional care for major depression most commonly includes antidepressant medication and/or a variety of psychological interventions such as cognitive-behavioral therapy, interpersonal therapy, or supportive group therapy. However, only half of Americans annually diagnosed with major depression obtain any conventional care, and only one-fifth complete the level of care that is likely to treat their major depressive episode [14]. Once initiated, both pharmacological and psychological therapies have dropout rates ranging from 20%-47%, with the higher rates reported in naturalistic studies that reflect “real world” treatment conditions [15–25]. Dropout from conventional care is influenced by several factors [16–24,26], including cost of office visits, duration of treatment, stigma regarding mental illness, insufficient therapeutic alliance with the clinician—and in the case of medications, intolerance to side effects.

Even when guideline-congruent treatment is completed, published studies indicate both pharmacological and psychological interventions for major depression are associated with relatively low remission rates of 28% to 46% following one course of therapy [27–30]. It has been suggested that even these remission rates may over-estimate the efficacy of conventional care in major depression, because of bias toward publishing positive studies [31–36]. Among individuals with poor response to one trial of pharmacotherapy, additional trials of medication and/or psychotherapy appear to increase the likelihood of remission [37–40], so that after 3 to 4 successive therapeutic trials, two-thirds of patients with major depression may ultimately achieve remission [41,42]. However, multiple treatment courses require considerable time, expense, perseverance and tolerance of potential side effects, and are unlikely to be pursued by many individuals with major depression. In sum, conventional therapies for major depression can be effective—and even lifesaving—for those who seek out care, tolerate full treatment courses without dropout, and have responsive symptoms, but many other individuals endure chronic functional impairment due to major depression that remains untreated, or does not remit with conventional care [14,43,44]. Thus, it is essential to develop novel and efficacious interventions for major depression that are safe, affordable, well accepted, and easily accessed.

### The yoga tradition

Yoga originated more than 5000 years ago in India as a comprehensive life discipline to harmonize body, mind, and spirit and to transcend suffering by developing an abiding awareness of one’s spiritual nature [45]. Circa 200 BCE, the Hindu sage Patanjali consolidated ancient yogic philosophy and practices into a classical Sanskrit treatise called the *Yoga Sutras* [45,46], describing eight components of yoga: ethical behavior, self-discipline, body postures, breath regulation, sensory withdrawal, and three progressively deeper meditative practices meant to cultivate union with one’s spiritual essence. The *Yoga Sutras* inform the many schools of yoga present today, although each school may emphasize different components of the classical tradition. Hatha yoga [47] is the most prevalent school of yoga in western countries and emphasizes physical components—such as body postures, breathing and relaxation techniques, and dietary practices. Most yoga classes available in North America are adaptations of hatha yoga, including Iyengar, Ashtanga, Vinyasa, Integral, Sivananda, and Bikram yoga.

### Yoga for depressive symptoms

In the United States, the practice of yoga is increasing markedly, with nearly 10% of the population in 2012 reporting some participation in yoga within the previous year [48]. Yoga exercises are associated with a relatively favorable risk/benefit profile [49–51], and are easily adapted for a wide range of practitioners—including frail seniors and individuals with medical problems for whom other physical activity may be difficult. The American public uses yoga not only to maintain wellness, and but also to treat specific health conditions [52–57]. Depression consistently ranks among the most common health conditions self-treated with yoga [52,53,58–60]. Notably, other mind-body therapies frequently used by the public to self-treat depression include breathing exercises, meditation, and relaxation techniques [58–60], all comprising key elements of yoga practice. The appeal of yoga as a treatment for depression may be related to its relatively low cost, ease of access, high social acceptance, and the perception that yoga “focuses on the whole person—mind, body, and spirit” [56].

Commensurate with increasing public interest in the mood benefits of yoga, the number of randomized controlled trials (RCTs) investigating yoga for depression grew significantly in the decade from 2005 to 2015, with several systematic reviews conducted in succession [61–67] to evaluate these studies. In all RCTs included in these reviews, the primary intervention involved yoga-based postures, breathing exercises, and/or techniques for meditation or relaxation. By the end of 2015, 24 RCTs of yoga for depression [68–91] were identified via systematic reviews, nearly a 5-fold increase from the 5 RCTs initially identified in 2005 by Pilkington et al [61].

Despite the increasing number of RCTs examining mood effects of yoga, investigation remains at an early stage of development. While most trials report measureable mood benefits from yoga, this collective evidence base must be interpreted with caution, as it may reflect publication bias toward positive studies. Moreover, systematic reviews [61,62,65,67] indicate that most published RCTs on yoga for depression are hampered by methodological problems. For example, few studies provided adequate information on methods for randomizing participants, concealing allocation schedule, blinding assessors and implementing other steps to minimize bias in outcomes. Blinding to the yoga allocation was typically not possible, but steps to address performance bias were not mentioned. Individual trials have tended toward small sample sizes and selective study populations—such as prenatal women, psychiatric inpatients, young adults, or community seniors—raising questions about generalizability of findings to the wider population with depression. From study to study, baseline symptoms of participants varied considerably: in some trials, participants were healthy with no significant mood symptoms [76,79,86,88]; in other trials, participants reported elevated depressive symptoms but had no diagnosed depressive disorders [73,77,80,82,91,92]; in yet other trials, participants were diagnosed with either major depression or dysthymia [78,81,83–85,87,89,93]; and finally, in a few trials, participants were diagnosed with only major depression [69–72,74,90]. While some trials specifically evaluated yoga as an adjunct to conventional depression care [68,69,74,90], others allowed some degree of co-intervention with conventional care in an unsystematic manner, confounding potential mood effects of yoga [78,81,83–85,87,92,93]. Additionally, the heterogeneity of yoga interventions used in various RCTs limits the ability to pool data for meta-analysis and draw conclusions about efficacy. Nonetheless, with caveats regarding these limitations, authors of 5 systematic reviews [61–64,67] and 2 meta-analyses [65,66] conclude there is preliminary evidence for the efficacy of yoga in the acute treatment of depression.

On the basis of existing clinical trial data, authors of systematic reviews generally suggest that yoga may be considered as an adjunctive therapy option in depressive disorders [63,65–67]. However, in at least one review [62], authors suggest there is reasonable evidence to consider yoga as second-line mono-therapy in major depression of mild-to-moderate severity. This possibility is supported by 3 early RCTs from India [70–72] in which yoga interventions were investigated as mono-therapy in major depression, and found to have significant mood benefits. In one trial [71], the yoga intervention was even comparable to pharmacotherapy with imipramine in achieving remission from depression. Since 2 of the trials involved psychiatric inpatients [71,72], and all 3 trials involved Indian participants perhaps having culture-specific, positive expectations about the efficacy of yoga, questions have been raised about applicability of findings to non-hospitalized, western populations with major depression. Pilkington et al [61] outline additional caveats in interpreting findings from these trials, but as a group, these 3 RCTs offer persuasive data for continued examination of yoga as mono-therapy for major depression.

### Study aims and hypotheses

The primary aim of this pilot RCT was to investigate potential mood benefits of hatha yoga as mono-therapy for mild-to-moderate major depression in a non-hospitalized, metropolitan U.S. population sample. Related aims were to assess feasibility and acceptability of the specific 8-week hatha yoga program pilot-tested in this trial, and to obtain empirical data for anti-depressant effect size estimation for a future, larger scale RCT. Our study was designed to recruit only individuals with mild-to-moderate major depression, and to exclude those with suicidal ideation, psychosis, or other functional impairment indicative of severe depression. Several large meta-analyses [94–96] of placebo-controlled antidepressant medication trials have compared mood benefits of active medication to placebo in hundreds of adults with major depression: while benefits of pharmacotherapy over placebo appeared significant for people with severe major depression, such benefits appeared minimal or even uncertain for those with mild-to-moderate major depression. Psychological treatments have not appeared any more efficacious than pharmacotherapy in this latter population [97,98]. Thus, by limiting our study sample to those with mild-to-moderate major depression, we aimed to recruit individuals for whom conventional care may offer uncertain benefit.

Due to resource constraints, we did not aim to investigate possible biological factors mediating anti-depressant effects of yoga. However, we aimed for a preliminary investigation of self-esteem and self-efficacy as possible psychological factors mediating mood improvement with yoga. Feelings of worthlessness (low self-esteem) and feelings of failure and powerlessness in meeting life challenges (low self-efficacy) are among the hallmark psychological features of major depression [99–101]. Classical yoga practice encourages compassionate, non-judgmental acceptance of one’s limitations and honoring of one’s life essence, thereby having potential to improve self-esteem. Concurrently, classical yoga practice moves one toward gradual mastery of physical and mental exercises initially perceived as difficult, so that self-efficacy may also improve. Thus, possible increases in self-esteem and self-efficacy from yoga practice may help to challenge and reverse the poor self-evaluation characteristic of depressive cognitions, facilitating recovery from depression. This theory has preliminary support from RCTs suggesting that self-efficacy and self-esteem tend to increase with yoga practice [102–107], and are associated with concurrent decline in depression [108–113]. Therefore, our study aimed to explore relationships between yoga practice and changes in depression severity, self-efficacy and self-esteem.

We hypothesized that relative to an attention control intervention, participation in an 8-week hatha yoga program for major depression would lead to statistically significant reduction in depression severity, our primary outcome, and statistically significant increases in self-efficacy and self-esteem, our secondary outcomes. Per recommendations from prior systematic reviews of yoga RCTs, we sought to reduce methodological issues in this trial by consistent adherence to good clinical research practice [114] and to reporting guidelines for non-pharmacological trials developed by the Consolidated Standards of Reporting Trials (CONSORT) [115].

## Methods

### Ethics statement

The Committee on Human Research, which is the Institutional Review Board (IRB) at the University of California, San Francisco (UCSF), reviewed and approved on April 29, 2010, the study protocol for this trial (CHR# H49362-35940-01). The approved protocol and corresponding CONSORT checklist are available as supporting information; see S1 and S2 Files. Recruitment took place from May 4, 2010 thru October 22, 2010, and the trial ended on January 15, 2011. The trial was registered at ClinicalTrials.gov and is accessible at https://clinicaltrials.gov/ct2/show/NCT01210651. Trial registration occurred after recruitment started, rather than prior to recruitment. The delay was due to investigators being unfamiliar with administrative procedures for registration, especially as some institutional procedures for registering cross-departmental trials were still being refined. The authors confirm that all ongoing and related trials for this intervention are registered.

After preliminary phone screening of individuals responding to recruitment ads, those who appeared eligible were invited to office screening visits with the principal investigator (PI). The PI engaged each participant in a detailed informed consent discussion about the study. Participants learned that if they met eligibility criteria and chose to enroll, they would be opting for randomization to one of the study interventions, instead of obtaining conventional depression care during the study. The PI explained that neither intervention is standard depression care, and offered participants referral to standard care in lieu of study enrollment. The PI discussed the risk of depression increasing over the 8-week study, in which case participants would be evaluated for study withdrawal and referral to urgent psychiatric care as appropriate. Only those who provided written informed consent were eligible to proceed with the rest of screening.

### Overview of study design

This was a prospective, single-center, single-blind, randomized, controlled, parallel group pilot trial of an 8-week hatha yoga program as mono-therapy in major depression. We recruited 38 adults from the San Francisco community who met diagnostic criteria for mild-to-moderate major depression, per evaluation with the Mini International Neuropsychiatric Interview and scores of 14–28 on the Beck Depression Inventory-II (BDI). At screening, individuals engaged in psychotherapy, antidepressant pharmacotherapy, herbal or nutraceutical mood remedies, or any mind-body practices were excluded. Eligible participants were randomized in a 1:1 ratio to one of two instructor-led intervention groups: a yoga practice group assigned to 90-minute hatha yoga exercises twice weekly for 8 weeks, versus an attention control education group assigned to 90-minute yoga history modules twice weekly for 8 weeks. Stratified block randomization ensured that each intervention group had equal numbers of participants with mild depression (per screening BDI scores of 14–19) versus moderate depression (per screening BDI scores of 20–28). Participants were informed at screening that anyone randomized to the education group would be offered 16 free hatha yoga classes, upon completion of the study, to learn the same exercises taught to the yoga practice group. Blinded assessors analyzed outcome data. The primary outcome was depression severity, measured by scores on the BDI every 2 weeks from baseline thru 8 weeks. Secondary outcomes were self-efficacy and self-esteem, measured by scores on the General Self-Efficacy Scale (GSES) and the Rosenberg Self-Esteem Scale (RSES) at baseline and at 8 weeks.

### Recruitment of participants

Participants were recruited via consecutive sampling of eligible individuals responding to IRB-approved ads posted throughout San Francisco, including libraries, community centers, shopping areas, online classifieds, and UCSF outpatient clinics and clinical trials websites.

Inclusion criteria for participation were: 18 years of age or older; written informed consent; all genders; all ethnicities; English proficiency sufficient for participation; ability to attend all required study visits; diagnosis of current major depression per screening Mini International Neuropsychiatric Interview (MINI); and depression symptoms of either mild severity (per screening BDI scores of 14–19) or moderate severity (per screening BDI scores of 20–28).

Exclusion criteria for participation were: use of antidepressant or psychotropic medication within 2 months of screening, or plan to initiate use during study; use of herbal or nutraceutical mood remedies—such as St. John’s Wort, fish oil, SAMe, or high-dose vitamins—within 2 months of screening, or plan to initiate use during study; use of psychotherapy at screening, or plan to initiate during study; use of yoga/mind-body practices at screening, or plan to initiate such practices during the study, other than allocated intervention; impaired cognition per score < 24 on screening Folstein Mental Status Exam; illicit drug use or diagnosis of substance use disorder within 3 months of screening MINI; diagnosis of bipolar disorder or other Axis I disorders comorbid with major depression per screening MINI; severe major depression per score > 28 on screening BDI; history of suicide attempts, suicidal ideation or psychosis; diagnosis of pregnancy; history of seizure disorder, uncontrolled hypertension, or carotid artery stenosis; and acute medical or psychiatric symptoms likely to interfere with study participation.

### Randomization and concealment of allocation

Each consenting participant completed a screening assessment with the PI, who was the study physician. The PI indicated on a Summary Form whether the participant—referenced only by a unique, computer-generated, random identification number—was eligible to continue in the study, and if so, whether depression severity was “mild” versus “moderate” according to screening BDI score. The PI had no further contact with participants, barring an adverse event requiring direct physician evaluation. Participants were assigned to intervention independently of the PI, with assignments concealed from the PI to prevent unconscious attempts to predict allocation schedule. For each eligible participant, the study coordinator faxed the Summary Form to the statistician, who worked remotely from the screening site and had no contact with participants. Using a SAS v9.0 program for block randomization stratified by depression severity, the statistician randomly assigned each participant to an intervention group, such that participants with mild depression, and those with moderate depression, were distributed equally between the two groups; block size was programmed to vary, minimizing risk of study personnel predicting allocation schedule. The statistician faxed back the Summary Form to the study coordinator, indicating the participant’s intervention assignment in a coded format. The study coordinator, who was not involved in delivery of interventions or outcome assessments, communicated the allocation to each participant. All Summary Forms were stored in locked file cabinets upon completion of fax transactions. Except the statistician, no study personnel had access to the SAS program, stored as an encrypted, password-protected electronic file.

### Interventions

#### Background and development of interventions

Prior to the current trial, the PI traveled to Bangalore, India, making site visits to two separate, well-established research centers with special expertise in conducting clinical trials of yoga: the Swami Vivekananda Yoga Research Foundation, and the Integrated Centre for Yoga at the National Institute of Mental Health and Neuroscience. At both sites, the PI met with investigators involved in studies of yoga therapies for depression, and made detailed observations of yoga protocols implemented in these trials. Yoga postures and breathing techniques in the current pilot study were adapted from a publication of the Swami Vivekananda Yoga Research Foundation, which consolidated and documented, on the basis of classical yoga theory, a well-defined set of yoga practices to alleviate depressive symptoms [116]. The PI pilot-tested a portion of this yoga sequence for depression in an open-label clinical trial with outpatients at a San Francisco mental health day program [117]; see S3 File. Based on preliminary data from this open-label trial, and interviews with several (n = 15) U.S. yoga teachers and practitioners, the hatha yoga sequence in the current study protocol was refined and finalized.

Once components of the hatha yoga intervention were established, a major challenge lay in selecting a suitable control intervention. Previous investigators have described the difficulties inherent in choosing an appropriate control when studying mind-body therapies for depression [118–120]. In theory, an optimal control would account for non-specific mood effects of a mind-body practice, but without obscuring or confounding any specific mood effects deriving from the “active ingredient” of that practice. In reality, however, it may be difficult to distinguish the non-specific effects of a mind-body therapy from its specific effects, and to identify the active ingredient(s) producing those specific effects. The RCT of yoga conducted by Rohini et al [72] is illustrative of these difficulties: in an innovative study design, investigators attempted to identify the active ingredient mediating anti-depressant effects of a particular yoga sequence, and then subtracted this component to create a “partial” yoga sequence that would serve as a credible placebo control. Yet, after 4 weeks, both the full and partial yoga sequences appeared to produce substantial and equivalent mood benefits. Perhaps this occurred because the partial yoga sequence still contained some portion of the active ingredient that provided a specific anti-depressant effect; alternatively, perhaps non-specific effects of both yoga sequences were powerful in alleviating depressed mood among participants.

Since mind-body therapies generally are not amenable to placebo control, other types of control intervention must be considered carefully, bearing in mind that the choice of comparator can significantly moderate effect size. In meta-analyses that examined yoga for depression [65], mindfulness-based therapies for depression [121], and exercise for depression [122], the effect size of the studied intervention decreased significantly in magnitude when the comparator changed from waitlist control/treatment-as-usual (TAU) to a more active control. In a recent meta-analysis of meditation RCTs, which included several yoga-based meditation interventions, Jain et al [119] point out that even TAU and waitlist controls may not be as “inert” in their mood effects as expected: they found that TAU/waitlist controls ranged from having moderate negative effects (-0.60) to large positive effects (1.54). This suggests that TAU/waitlist controls may function variably as “nocebos” with detrimental effects due to negative expectation, or as placebos with salutary effects due to positive expectation, or even as interventions with specific mood effects, depending on how much anti-depressant treatment occurs in the TAU arm. Jain et al suggest that psycho-education groups may offer the best option as control interventions in RCTs of meditation for depression, as these groups are associated with a fairly consistent range of small, positive effect sizes (0.02 to 0.54) when used as comparators in depression trials.

We considered the option of a psycho-education group as the comparator in this trial, but had concerns about potential nocebo effects. We reasoned that participants recruited to our study might have a particular interest in yoga or mind-body therapies, and perhaps the prospect of yoga therapy, more so than the prospect of conventional care, had finally motivated them to address their depressive symptoms. For such participants, randomization to a conventional psycho-education group, with no connection to mind-body therapies, might carry more risk of exerting a negative effect on mood than if these individuals had not had a pre-existing interest in alternative medicine. Therefore, in designing our control, we retained the format of education modules associated with conventional psycho-education groups, but modified the content to focus on yoga-related themes. Psycho-education modules for major depression typically provide information and facilitate discussion on the etiology and epidemiology of depression, as well as symptoms, treatment options and coping strategies. Instead, our education modules provided information and facilitated discussion on yoga origins, history and philosophy, as well as major yoga schools and teachers in India and abroad. We anticipated that delivery of yoga-themed content to control participants, along with an opportunity to learn hatha yoga exercises on study completion, would help defray disappointment and possible nocebo effects related to allocation.

#### Intervention setting and precautions

Both interventions took place at the UCSF Osher Center for Integrative Medicine, and all data were collected at this site. Within one week of the office screen, each randomized participant was assigned to begin attending his or her allocated group sessions twice weekly for 8 weeks total. Group instructors took attendance. Sessions for both interventions were available on a rolling basis throughout the study until the last participant completed the 8-week protocol.

It was not possible to blind either instructors or participants to the allocated intervention. The PI took several steps to mitigate any resulting performance bias. Prior to recruitment, when training instructors, the PI highlighted the potential of non-specific mood benefits to result from allocation to either intervention, and stressed the importance of holding a neutral attitude with participants regarding outcomes. Once the trial began, the study coordinator made periodic unannounced visits to both intervention groups to monitor instruction quality and adherence to protocol, and the PI met regularly with each instructor to discuss any issues in delivering interventions. To minimize performance bias in participants, the PI informed prospective participants at screening that both interventions might have positive effects on mood due to the process of learning novel information; the PI also mentioned that for people randomized to the education group, information gained on yoga history and philosophy might enhance subsequent learning and practice of hatha yoga exercises during free classes offered on study completion.

To protect participants against adverse consequences of any psychiatric decline during the study, several safety measures were in place: (a) During the pre-recruitment phase, all study personnel having contact with participants were trained by the PI to recognize and report potential signs of worsening depression in participants. (b) Instructors observed participants closely during the twice-weekly intervention sessions, monitoring for evidence of clinical decline. (c) Formal monitoring of depression severity occurred every 2 weeks via the BDI, with plan for direct evaluation by the PI, a Board-certified psychiatrist, should any participant indicate suicidal ideation on Item 9 of the BDI. (d) Study personnel undertook vigorous follow-up of any participant absenteeism. (e) Direct evaluation by the PI was available should any participant report suicidal ideation, psychosis or subjective worsening of depression during the study.

#### Attention control intervention

Participants randomized to the attention control group attended instructor-led, 90-minute education modules on yoga history and philosophy. The 16 modules comprising the attention control intervention were adapted from a series of community lectures previously given by the PI; see S4 File. Since participants would join the attention control group on a rolling basis, the education modules were designed to function as stand-alone sessions, rather than requiring presentation in a specific sequence. The instructor was a registered yoga teacher with additional certification by the Chopra Center as a Vedic Master Educator in yoga philosophy. In each module, short lectures were enhanced by documentary film clips carefully selected to focus on key historical figures and major branches of the yoga tradition, rather than depicting details of specific yoga practices. Interactive dialogue was encouraged between instructor and participants. Modules were intended to control for non-specific mood benefits of the hatha yoga intervention, from factors such as attention from study personnel, peer interaction, time spent away from routine activities, and anticipation related to mastering novel yoga-related information.

#### Hatha yoga intervention

Participants randomized to the hatha yoga group attended instructor-led, 90-minute practice sessions comprised of classical yoga breathing techniques, mindful body postures, and final deep relaxation pose (Table 1). Several postures featured a chest-opening element, traditionally thought to help alleviate depression. The same hatha yoga sequence was used in all sessions. The instructor was a certified yoga teacher and also a licensed, registered nurse with 5 years of experience in teaching hatha yoga to adults with a range of medical issues and yoga skill levels. During sessions, yoga practices were broken down into component elements and taught progressively to each student in accordance with his or her ability. The nurse-instructor encouraged participants to remain within their range of motion or comfort, and made appropriate accommodations for those with limitations in flexibility or tolerance for any exercise. Props, such as blocks and bolsters, were used to support participants in learning and holding yoga poses safely, particularly backbends or inversions. In the unlikely event of injury, the nurse-instructor was equipped to provide immediate medical triage and assistance.

**Table 1:** Hatha yoga sequence*.

Segment	Description
Introduction & Guidelines (5 mins)	Instructor welcomes all participants, including those joining session for first time. Reviews guidelines for yoga practice, encouraging participants to remain within their range of motion or comfort in performing any exercise. Emphasizes that accommodations are available for each exercise, so that those with limitations may still take part. Each participant settles into a comfortable seated position with erect spine and neck, either on chair or cross-legged on the ground.
Breath Regulation (20 mins)	*Ujjayi* (Victorious Breath): deep, slow breathing completed at rate of about 4 respirations/minute against mild airway resistance. Participants learn techniques to slightly contract laryngeal muscles and partially close the glottis to maintain mild airway resistance during inspiration and expiration.
	*Bhastrika* (Bellows Breath): breath is forcefully inhaled and then exhaled through nostrils at a rate of about 20–30 respirations/minute while using strong abdominal muscle contractions.
	*Nadi Shodana* (Alternate Nostril Breathing): relaxed breathing pattern with inhalation and exhalation done through just one nostril at a time. Breathing occurs at a normal rate of 12 respirations/minute.
	*Brahmari* (Bumblebee Breath): Slow breathing is done while eyes and ears are covered via specific placement of hands over face and external ear flaps. There is long slow inhalation and then a gentle humming sound is made with exhalation. Breathing occurs at a rate of about 6 respirations/minute.
Mindful Poses & Movement (50 mins)	*Ardhakati Chakrasana* (Half Waist Wheel Pose): Participants stand upright and then gently bend from waist sideways to the right as far as comfortable, while stretching left arm upward. Returning to an upright stance, they repeat exercise, but bending to the left while stretching right arm up.
	*Ardha Chakrasana* (Half Wheel Pose): Participants stand upright with hands supporting the lower back. They gently bend backwards from the lower lumbar region as far as comfortable, allowing neck to drop slowly while hands continue to support the lower back.
	*Pada Hastasana* (Hands to Feet Pose): Participants stand upright with arms raised, and gently bend forward until torso is parallel to ground; participants flexible enough continue to bend forward until hands are touching the feet and the head is touching the knees.
	*Bhujungasana* (Cobra Pose): Participants lie on ground in prone position. They gently arch the head and spine backwards as far as comfortable, supporting the torso with arms bent at elbow and palms firmly on ground.
	*Dhanurasana* (Bow Pose): Participants lie on the ground in a prone position. Then, knees are bent with feet moving toward buttocks as far as possible, and the neck gently arched backward. Participants flexible enough gently grasp the ankles with their hands.
	*Sarvangasana* (Shoulder Stand): Participants lie on the ground in a supine position. Participants with sufficient strength and flexibility raise legs slowly until the legs are perpendicular to the ground; other participants position their legs against a wall so that the legs are as perpendicular to the ground as possible. Those with sufficient strength and flexibility then raise the buttocks and trunk off the ground, keeping the shoulders and elbows firmly on the ground and supporting the back with both palms while the legs remain perpendicular to the ground.
	*Matsyasana* (Fish Pose): Participants lie on ground in supine position. Hands are placed palm down underneath the back, with fingers pointing toward spine. Pressing down on palms, the chest is slowly raised and back is arched as far as comfortable, with weight of torso supported on elbows.
	*Setu Bandhasana* (Bridge Pose): Participants lie on ground in a supine position. Then the knees are bent while keeping the soles of the feet on the ground. Arms are kept alongside the torso, but with palms on the floor. The hips are lifted off the floor towards the ceiling as far as comfortable, keeping the feet and palms flat on the floor.
	*Balasana* (Child Pose): Participants kneel on ground with feet tucked under the buttocks, if possible. Participants then bend forward at waist, bringing chest as close to knees as possible, and resting forehead on the ground, if flexible enough. Arms are extended on the ground, palms down.
	*Vakrasana* (Twisted Pose): Participants sit on ground with both legs outstretched. Then the left leg is bent at knee with sole of the left foot placed on ground alongside the right knee. The upper part of the body is twisted to the left as much as possible, keeping shoulders in plane with the chest and supporting the torso on the left arm, with left hand firmly planted palm-down on the ground. Elbow of the right arm should be straight and pressed against the lateral surface of the bent left knee. After holding this pose, participants return torso, arms and legs to the beginning position. Then participants repeat pose so that right leg is bent and upper body twists to the right side.
	*Ustrasana* (Camel Pose): Participants kneel on ground, keeping neck and spine perpendicular to ground. Then spine is arched back gently so that chest puffs out. Those flexible enough reach hands back one at a time to grasp heels. Others reach back to blocks placed on either side of feet.
Final Deep Relaxation (15 mins)	*Shavasana* (Corpse Pose): Participants lie on ground in a supine position, with eyes closed. Arms are stretched away from the torso, palms up. Legs are separated about a shoulder width apart, and the feet rotate out slightly. With calm, soft verbalizations, the instructor guides participants into deep relaxation in this pose, as the breath gradually settles into a slow, restful rhythm.

* Hatha yoga exercises adapted from: Nagarathana, R, Nagendra, HR. Yoga Practices for Anxiety and Depression. Bangalore: Swami Vivekananda Yoga Prakashana; 2001.

### Instruments and measures

#### Physician evaluation form

During office screens, the PI used this project-specific form to record each participant’s demographic data, medical history, physical exam findings, and study eligibility.

#### Mini International Neuropsychiatric Interview (MINI)

During office screens, the PI administered the Mini International Neuropsychiatric Interview (MINI 6.0.0) to diagnose major depression and to exclude co-morbid psychiatric disorders in participants. The MINI is a validated, structured, diagnostic interview tool [123,124] with questions that parallel symptoms in the Diagnostic and Statistical Manual of Mental Disorders [125]. The MINI can be administered in 15 minutes by trained clinicians and has high diagnostic reliability with lengthier instruments developed by the World Health Organization.

#### Folstein Mini Mental Status Exam (MMSE)

During office screens, the PI administered the Folstein Mini Mental Status Examination (MMSE) to assess cognition of participants. The MMSE is validated and widely used instrument to evaluate cognitive status via tests of orientation, attention, memory, and basic language skills [126,127]. Total score on the MMSE ranges from 0–30. Performance varies by age and educational level, but scores below 24 may suggest cognitive impairment.

#### Beck Depression Inventory-II (BDI): Primary outcome measure

The Beck Depression Inventory-II (BDI) was first completed by all participants at the office screen to help determine eligibility, and then repeated by randomized participants just prior to baseline session and just after sessions at Weeks 2, 4, 6 and 8. The BDI is a 21-item, validated instrument for the self-report of depressive symptoms, appearing in hundreds of studies worldwide [128–130]. Individual item scores are summed for a total BDI score ranging from 0–63. BDI scores from 0–13 suggest absent to minimal depressive symptoms, from 14–19 mild symptoms, from 20–28 moderate symptoms, and from 29–63 severe depressive symptoms.

#### General self-efficacy scale: Secondary outcome measure

Participants completed the General Self-Efficacy Scale (GSES) just prior to the baseline session and just after the 8-week session. The GSES is a validated, 10-item, self-administered instrument [131,132] designed to assess self-efficacy—that is, the belief that one’s actions lead to successful outcomes in coping with difficult life demands. Individual item scores are summed for a total GSES score of 10–40. Higher scores indicate stronger belief in one’s self-efficacy.

#### Rosenberg self-esteem scale: Secondary outcome measure

Participants completed the Rosenberg Self-Esteem Scale (RSES) just prior to the baseline session and just after the 8-week session. The RSES is a validated self-administered 10-item scale [133,134] to assess feelings of self-worth. Individual item scores are summed for a total RSES score of 0–30. Scores below 15 suggest possible problems with self-esteem, while scores of 15–25 suggest typical self-esteem, and scores of 26–30 suggest high self-esteem.

#### Enrollment Log

**Fig 1:**
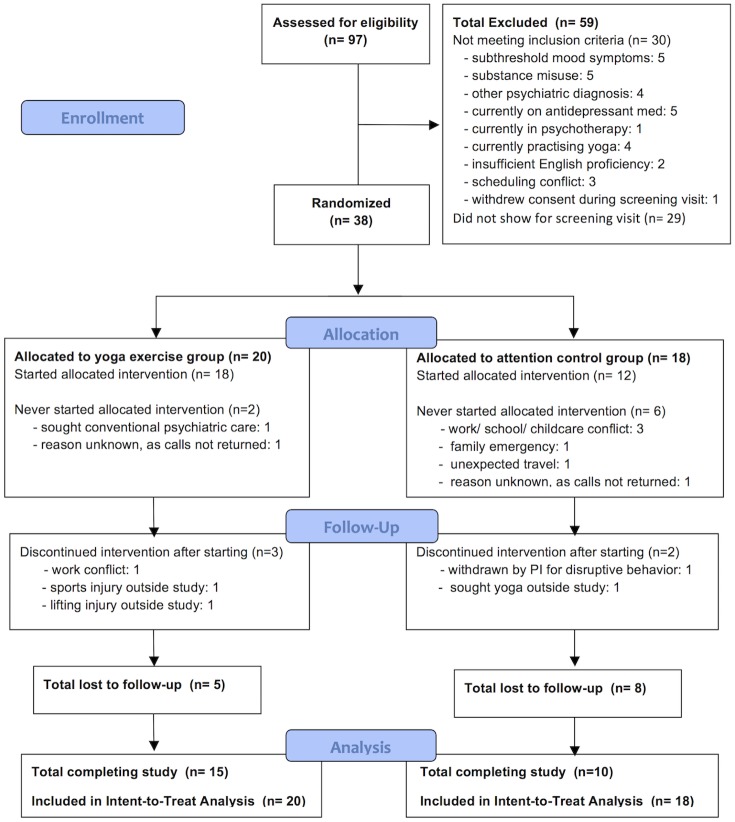
Participant flow diagram.

Throughout the trial, the study coordinator maintained a project-specific Enrollment Log tallying the screening, accrual, allocation and attrition data depicted in Fig 1.

#### Session Logs

Instructors used project-specific Session Logs to record attendance at all intervention groups and to make notes regarding progress and engagement of individual participants.

#### Adverse Events Log

Monitoring for adverse events occurred continuously throughout the clinical trial by study personnel having contact with participants. Any adverse event in either intervention group was reported immediately to the PI, who maintained a written, project-specific Adverse Events Log and provided guidance and oversight to study personnel regarding appropriate responses.

#### Final Feedback Questionnaire

The project-specific Final Feedback Questionnaire requested study completers to disclose use of any co-interventions and to rate instructor quality, content quality, and difficulty and acceptability of the allocated intervention. Suggestions for improvement were also solicited.

### Outcome data collection

Since all primary and secondary outcome data in this trial were to be obtained from self-report instruments (BDI, RSES and GSES), it was possible to have instruments self-administered by participants. Five envelopes, corresponding to the 5 measurement points, were prepared for each participant prior to group allocation. Each envelope contained blank BDI, RSES, and GSES forms as appropriate for the corresponding measurement point. Each envelope was pre-labeled with participant’s study ID number and time-point at which contents should be completed. At baseline session and subsequent 4 sessions comprising measurement points, participants went to a designated file box that held their 5 pre-labeled envelopes, and picked out the envelope labeled for that time-point. In an unstaffed classroom, participants opened their envelopes, completed enclosed forms using written instructions, sealed forms in envelopes, and deposited envelopes into a slotted lockbox. At the end of each intervention day, all sealed envelopes in lockbox were delivered to study personnel blind to participant allocations. Envelopes were unsealed and enclosed outcome data recorded electronically on a secure drive, with participants identified only by study ID numbers. Since hatha yoga sessions were held on the same days as control group sessions, personnel opening sealed envelopes could not predict the intervention group from which a given envelope originated.

### Data analysis methods

#### Overview and sample size

Statistical analyses were conducted in a blinded manner, with de-identified data provided to the statistician in electronic files labeled only as “Dataset A” and “Dataset B.” Data were reviewed to ascertain that distributions of measures met the assumptions of the statistical tests to be used. Analyses were performed with Stata software, Version 14.1. All statistical tests were two-tailed, and a p-value < 0.05 was set *a priori* as the threshold for statistical significance.

Sample size in this pilot trial was based primarily on participant and resource availability during the recruitment timeline. However, we aimed to collect data from a sufficient number of study completers to estimate a Cohen’s d anti-depressant effect size of yoga relative to control. Prior to recruitment, we calculated that a sample size of 20 participants in each intervention group, even with 25% attrition, would still provide 80% power to detect a between-group effect size of d  =  1.0, using an independent samples t-test with two-tailed alpha = 0.05. Anti-depressant effect sizes of this magnitude were found in prior yoga RCTs with small samples [70,73,81,86].

#### Evaluating feasibility and acceptability

Feasibility and acceptability of this trial were evaluated, in part, via accrual, retention and adherence rates tallied from the Enrollment and Session Logs. Our target accrual rate was set at 8 randomized participants per month over 5 months, to achieve a total sample size of 40. Retention rate was defined as the percentage of randomized participants completing 8-week study measures. Adherence rate was defined as the percentage of allocated sessions attended by participants; adherence was examined only in study completers, since retention data would already reflect any non-adherence due to dropout. Feasibility and acceptability were also evaluated via data from the Adverse Events Log and the Final Feedback Questionnaires.

#### Evaluating participant characteristics

Salient demographic and clinical characteristics of participants were evaluated via descriptive statistics. Descriptive measures were constructed for the sample as a whole, and also for sub-groups based on intervention assignment. Frequencies were calculated for categorical variables, and means with standard deviations were calculated for continuous variables. To examine whether clinical and demographic characteristics of randomized participants were distributed equally between the two groups, categorical variables were compared via Fisher’s exact test and continuous variables via the Wilcoxon rank sum test.

#### Approach to intent-to-treat analyses of depression severity

The main analysis of depression severity, measured by BDI scores, used an intent-to-treat approach and incorporated data from all randomized participants regardless of adherence to protocol or premature dropout. We hypothesized that over the 8-week study period, participants in the yoga practice group would achieve a statistically significant reduction in BDI scores compared to participants in the control group. We estimated and tested a random-effects generalized least squares (GLS) regression model for depression severity, using BDI scores obtained at baseline, 2 weeks, 4 weeks, 6 weeks, and 8 weeks. Baseline BDI scores were obtained just prior to participants beginning the first allocated intervention session, and final BDI scores were obtained just after the last intervention session at 8 weeks. For randomized participants who dropped out before baseline BDI scores could be obtained, data imputation was done via carrying forward BDI scores from screening. Trends in BDI scores across the five assessment points were modeled for each intervention group, and maximum likelihood estimations were used to derive an adjusted mean BDI score for each intervention group at each assessment point. BDI scores were modeled as correlated within participants, but independent between participants. Demographic and clinical covariates appraised at screening—such as age, gender, ethnicity, depression severity, and so on—were each examined in the preliminary statistical model as controls, and impact on depression outcomes was evaluated. Covariates that proved to be statistically non-significant were omitted in the final model. The model included effects for intervention group, to test whether change in mean BDI scores over a given interval varied significantly by group, and for intervention-by-time, to test whether mean BDI scores within each group varied significantly between assessment points.

The main analysis of depression severity was followed by two additional intent-to-treat GLS regression analyses. The first of these was a sensitivity analysis to examine an alternate method for handling missing BDI scores at baseline. In the sensitivity analysis, we considered each randomized participant’s BDI score from screening as the first of six repeated outcome measures, rather than a pre-intervention covariate in the model. Results from the sensitivity analysis were then compared to those from the main analysis. In the second follow-up analysis, we included as a key predictor the number of assigned intervention sessions completed by randomized participants at each assessment point, and examined the interaction of this variable with intervention group and with depression outcomes.

#### Approach to completers analyses of depression severity

Following intent-to-treat analyses of BDI scores, we re-analyzed BDI scores in study completers (n = 25), defined as those participants providing both baseline measures and final 8-week measures. Among study completers, we first examined the interaction of BDI scores with intervention group and with intervention-by-time, using a random-effects GLS regression model. We hypothesized that completers in the yoga group would have greater decline in BDI scores than completers in the control group. Next, we evaluated the number of completers in each intervention group who achieved remission from depression, defining remission as a BDI score ≤ 9 at the final 8-week assessment [135–137]. We hypothesized that study completers in the yoga group would be more likely than those in the control group to achieve remission; we tested this hypothesis by using Fisher’s exact test to compare remission rates in the two groups.

#### Approach to analyses of self-efficacy & self-esteem

Analyses of GSES scores (measure of self-efficacy) and RSES scores (measure of self-esteem) were limited to study completers (n = 25), since the GSES and RSES were collected only at baseline and at 8 weeks. We hypothesized that over the 8-week study, yoga participants would show greater increase in GSES scores and in RSES scores compared to controls. Using paired t-tests, we first analyzed within each group whether mean GSES scores differed between baseline and 8 weeks. Next, using an independent samples t-test, we determined whether mean change in GSES scores differed between the two intervention groups. We repeated this procedure to analyze change in RSES scores within each group, and then between groups.

#### Estimating effect size

Following tests of statistical significance, we analyzed study outcomes for clinical significance via estimates of effect size. For this analysis, we examined outcome data from study completers in each intervention group (n = 15 in yoga group, n = 10 in control group). For each outcome measure, we determined the mean change score over 8 weeks, along with corresponding standard deviation. The magnitude and direction of the yoga intervention’s effect, relative to the control, was then calculated for each outcome via the formula for Cohen’s d [138], along with 95% confidence intervals. Following conventions proposed by Cohen [139], we considered an effect size with an absolute value ≥ 0.2 and < 0.5 to be a small but appreciable clinical effect; an absolute value ≥ 0.5 and < 0.8 to be a moderate clinical effect; and an absolute value ≥ 0.8 to represent a large clinical effect.

## Results

### Overview of enrollment

Over a 5-month recruitment period, 97 individuals were screened for eligibility (Fig 1). A total of 59 individuals were excluded from study participation, for reasons described in Fig 1. A total of 38 participants met inclusion criteria and were randomized to study interventions, with 20 participants allocated to the yoga group and 18 participants allocated to the attention control group. Among those randomized, 8 participants never started their allocated interventions (n = 2 in yoga group, n = 6 in control group), for reasons listed in Fig 1. An additional 5 participants dropped out before the final session at 8 weeks (n = 3 in yoga group, n = 2 in control group), for reasons listed in Fig 1. Thus, a total of 13 participants were lost to follow-up (n = 5 in yoga group, n = 8 in control group). Final 8-week measures were obtained from 75% (n = 15) in the yoga group, 56% (n = 10) in the control group, and 66% (n = 25) in the total sample. Despite any participants lost to follow-up, all 38 randomized participants were included in intent-to-treat analyses of depression severity, the primary outcome.

### Findings on feasibility & acceptability

#### Accrual, retention and adherence

The average accrual rate was 7.6 randomized participants per month, close to the target of 8 we had aimed for. Participants were drawn largely from non-clinical community venues. Retention rate in the total sample was 66%, and the retention rate of 75% in the yoga group and 56% in the control group were statistically comparable (Fisher’s exact test p-value = 0.307). As evident from Fig 1, nearly all dropout by control participants occurred very early in the study, after learning the intervention allocation, and before attending the first allocated session. When we re-evaluated retention among participants (n = 30) who attended at least the first allocated session, retention rates in the two groups were identical at 83%. Adherence rate in study completers was found to be 65%, and the adherence rate of 74% in yoga group completers and 51% in control group completers were statistically comparable (unpaired t-test p-value = 0.10).

#### Report of adverse events

Aside from 2 instances of musculoskeletal injury (back strain from heavy lifting; sprained ankle from soccer) that occurred outside study sessions, there were no serious adverse events related to participation in either intervention. In minor adverse events, 5 of 18 participants (28%) who attended at least one yoga practice session reported transient musculoskeletal discomfort when learning yoga poses; discomfort resolved as participants adjusted to the yoga regimen and implemented accommodations for limited flexibility. No adverse events occurred during breathing exercises or the final relaxation segment.

#### Qualitative Feedback from participants

The Final Feedback Questionnaire was obtained from 9 of 15 study completers in the yoga group (60%) and 5 of 10 study completers in the control group (50%). Participants in neither group reported co-intervention with pharmacotherapy or mind-body practices outside study sessions, but 1 participant in the control group reported co-intervention with psychotherapy. Participants in neither group felt that depressive symptoms interfered with learning and mastering the content presented. Overall, 7 of 9 yoga participants and 4 of 5 control participants made positive comments about their learning experience, appreciating the pace of instruction, instructor quality, and content quality. To improve the study in future, 4 of 9 yoga participants suggested increasing practice sessions to 3 times weekly, and 5 of 9 yoga participants suggested optional yoga history modules offered to those who completed the yoga practice group. There were no suggestions for change regarding the yoga history modules, except from 1 of 5 control participants, who thought modules would be improved by emphasizing scientific studies of yoga rather than yoga philosophy.

### Findings on participant characteristics

Demographic and clinical characteristics of all randomized participants are shown in Table 2. There were no statistically significant differences between the two intervention groups. Our total sample was comprised of about two-thirds women and one-third men, ranging in age from 22 to 72 years and having a mean age of 43.4 years (SD = 14.8). While 58% of participants were of European descent, participants of Asian, Latino, African and multi-ethnic descent were also represented, reflecting the diversity of the San Francisco area. A large proportion (89%) of participants were single (divorced, widowed or never married). The majority (63%) had a 4-year college degree or higher, and another 32% had attended 2 years of college. Of the total sample, 53% were employed and 13% retired, with the remaining one-third either in school or unemployed. Nearly two-thirds of the sample (64%) had some previous yoga exposure—typically erratic past attendance at a few community yoga classes, rather than regular practice; anyone engaged in yoga practice at screening had been excluded from study participation.

**Table 2:** Demographic and clinical characteristics of participants.

Covariate	Covariate Sub-category	Full Sample, N = 38	Yoga Group, N = 20	Control Group, N = 18	P-value
Age, in Years: Mean (SD), Range		43.4 (14.8), 22–72	43.1 (15.2), 22–64	43.8 (14.7), 23–72	0.89
Female Gender		26 (68%)	15 (75%)	11 (61%)	0.49
Married		4 (11%)	1 (5%)	3 (17%)	0.33
Ethnicity*					0.77
	European descent	22 (58%)	13 (65%)	9 (50%)	
	Asian descent	6 (16%)	2 (10%)	4 (22%)	
	Latino descent	4 (11%)	2 (10%)	2 (11%)	
	African descent	3 (8%)	1 (5%)	2 (11%)	
	Multi-ethnic	3 (8%)	2 (10%)	1 (6%)	
Highest Education					0.19
	High school diploma	2 (5%)	0 (0%)	2 (11%)	
	Two years of college	12 (32%)	5 (25%)	7 (39%)	
	Four-year college degree or higher	24 (63%)	15 (75%)	9 (50%)	
Occupational Status*					0.29
	Employed	20 (53%)	8 (40%)	12 (67%)	
	Unemployed	9 (24%)	7 (35%)	2 (11%)	
	Student	4 (11%)	2 (10%)	2 (11%)	
	Retired	5 (13%)	3 (15%)	2 (11%)	
Previous Yoga Exposure		24 (64%)	10 (50%)	14 (78%)	0.10
Number of Previous Depressive Episodes*					0.74
	None	4 (11%)	3 (15%)	1 (6%)	
	1 Episode	22 (58%)	12 (60%)	10 (56%)	
	2 Episodes	9 (24%)	4 (20%)	5 (28%)	
	3 Episodes	3 (8%)	1 (5%)	2 (11%)	
Number of Previous Antidepressant Trials*					0.24
	None	13 (34%)	10 (50%)	3 (17%)	
	1 Trial	15 (39%)	5 (25%)	10 (56%)	
	2 Trials	5 (13%)	2 (10%)	3 (17%)	
	3 Trials	4 (11%)	3 (15%)	1 (6%)	
	4+ Trials	1 (3%)	0 (0%)	1 (6%)	
Screening BDI Score: Mean (SD)		22.4 (4.5)	22.8 (4.4)	22.4 (4.6)	0.81
	Score 14–19 (Mild depression)	8 (21%)	4 (20%)	4 (22%)	
	Score 20–28 (Moderate depression)	30 (79%)	16 (80%)	14 (78%)	
Screening MMSE Score: Mean (SD)		28.0 (1.6)	27.7 (1.8)	28.4 (1.2)	0.15

BDI = Beck Depression Inventory-II-II; MMSE = Folstein Mini Mental Status Exam

* Percentage total of sub-categories may not equal 100% when summed, due to rounding.

The majority of our sample (79%) had depression of moderate severity; our stratified block randomization method was successful in distributing participants with mild depression, and those with moderate depression, in equal proportions between the two groups. The great majority of participants had experienced previous episodes of major depression, with 58% having one prior episode, 24% having 2 prior episodes, and 8% having 3 prior episodes. About a third of participants had never tried antidepressant medication, while 39% had been on one previous medication trial, and the remaining 27% had been on 2 or more medication trials. On the Folstein Mini Mental Status Exam, participants met cognitive norms for age and education.

### Results from intent-to-treat analyses of depression severity

**Fig 2:**
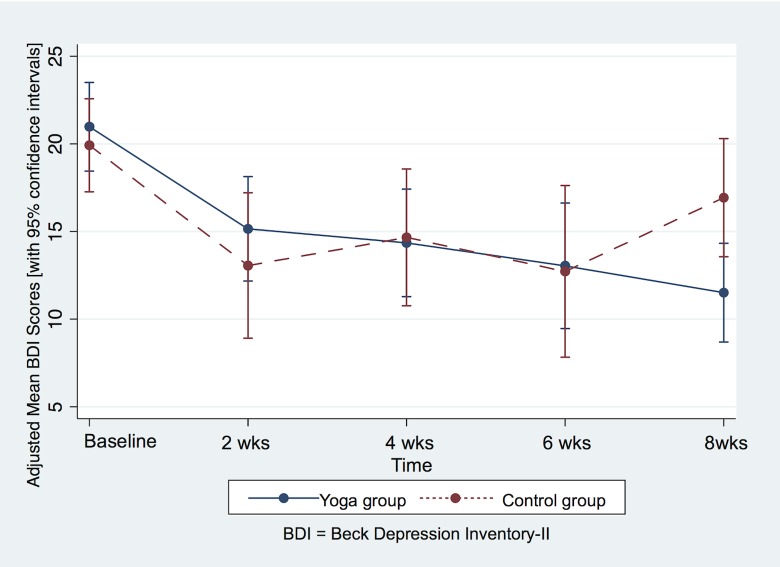
Intent-to-treat analysis: Adjusted mean BDI scores plotted by time.

In the main intent-to-treat GLS regression analysis of depression outcomes, BDI scores from all randomized participants (n = 38) were evaluated from baseline thru study completion. As none of the appraised demographic or clinical covariates of participants proved to be significant in the preliminary model, they were omitted in the final analysis. Fig 2 depicts time-plots of the adjusted mean BDI scores, along with 95% confidence intervals, for each intervention group at each assessment point. As hypothesized, a statistically significant group-by-time interaction emerged, indicating that in comparison to the control group, those in the yoga practice group experienced a greater decline in depression symptoms over the 8-week study (p-value = 0.034).

The adjusted mean BDI scores and 95% confidence intervals depicted in Fig 2 time-plots are presented in numeric form in Table 3. From baseline to final assessment at 8 weeks, the adjusted mean BDI score in the yoga group decreased by 9.47 points [95% CI, 12.37 to 6.57]; during the same interval, the adjusted mean BDI score in the control group decreased by 2.99 points [95% CI, 6.43 to 0.45]. The difference between the two groups in these 8-week BDI change scores is statistically significant (p-value = 0.005). However, the more conservative p-value of 0.034 obtained from the GLS regression analysis is likely a more reliable estimate of statistical significance when comparing depression outcomes between the two groups, since the regression analysis takes into account the trajectory of change in BDI scores over the entire 8 weeks, rather than focusing on change between only two time-points.

**Table 3:** Intent-to-treat analysis: Adjusted mean BDI scores by time, with 8-wk change scores.

Time	Adjusted Mean BDI Score [95% CI]		8-wk BDI Change Score [95% CI]	
	Yoga Practice Group, n = 20	Attention Control Group, n = 18	Yoga Practice Group, n = 20	Attention Control Group, n = 18
Baseline	20.98 [18.45, 23.51]	19.92 [17.26, 22.58]	-9.47 [-12.37, -6.57]	- 2.99 [-6.43, -0.45]
2 wks	15.15 [12.17, 18.12]	13.06 [8.91, 17.20]		
4 wks	14.35 [11.28, 17.41]	14.66 [10.76, 18.56]		
6 wks	13.04 [9.46, 16.62]	12.72 [7.82, 17.62]		
8 wks	11.51 [8.69, 14.33]	16.93 [13.56, 20.30]		

Two additional intent-to-treat GLS regression analyses augmented findings from the main analysis of depression outcomes. In the sensitivity analysis, the yoga group again showed more decline in BDI scores over the 8-week study compared to controls; this group-by-time interaction was associated with a p-value of 0.036 in the sensitivity analysis, nearly identical to the p-value of 0.034 in the main analysis. In the second analysis, which examined BDI scores after controlling for number of sessions attended by each participant, the yoga group continued to show greater reduction in BDI scores by 8 weeks than did the control group (p-value = 0.045).

### Results from completers analyses of depression severity

Among study completers (n = 25), GLS regression analysis of depression severity demonstrated a statistically significant group-by-time interaction; as hypothesized, BDI scores over the 8-week intervention period declined more significantly in the yoga group than in the control group (p-value = 0.014). In the analysis of remission, 9 of 15 completers in the yoga group achieved remission (60% remission), while 1 of 10 completers in the control group achieved remission (10% remission). The difference in these remission rates was statistically significant (Fisher’s Exact Test p-value = 0.018).

### Results from analyses of self-efficacy & self-esteem

Analyses of GSES scores (measure of self-efficacy) and RSES scores (measure of self-esteem) were limited to study completers (n = 25), and are summarized in Table 4.

**Table 4:** Analyses of GSES scores and RSES scores in study completers.

Intervention Group	OutcomeMeasure	Mean Baseline Score (SD)	Mean 8-wk Score (SD)	Mean Change Score (SD)	P-value: Within-group	P-value: Between-group
Yoga (n = 15)	GSES	26.87 (3.09)	29.0 (3.89)	2.13 (2.07)	0.001	0.50
Control (n = 10)		28.5 (4.33)	30.0 (2.67)	1.5 (2.4)	0.076	
Yoga (n = 15)	RSES	14.6 (3.48)	17.47 (3.87)	2.87 (3.42)	0.006	0.053
Control (n = 10)		16.0 (3.77)	16.2 (3.88)	0.2 (3.01)	0.838	

From baseline to 8 weeks, GSES scores among completers in the yoga group increased significantly (p-value = 0.001). However, over the same interval, GSES scores among completers in the control group also showed an increase, trending toward statistical significance (p-value = 0.076). Therefore, contrary to our hypothesis, increase in self-efficacy, as measured by GSES change scores, was comparable in both groups of completers (p-value = 0.50). From baseline to 8 weeks, RSES scores increased significantly among completers in the yoga group (p-value = 0.006), but not in the control group (p-value = 0.838). However, the difference between the two groups in RSES change scores narrowly missed the threshold for statistical significance (p-value = 0.053). Thus, contrary to our hypothesis, increase in self-esteem, as measured by RSES change scores, was comparable in both groups of completers.

### Results on effect size

Using change score data from study completers (n = 25), the effect size of yoga, relative to control, was estimated for each outcome measure (Table 5). The Cohen’s d effect size of yoga in reducing BDI scores was relatively large at -0.96 [95% CI, -1.81 to -0.12]. All values in the confidence interval were noted to be negative, indicating that the direction of effect, at least 95% of the time, would be toward reducing BDI scores. However, some values in the confidence interval were noted to fall below an absolute value of 0.20, the threshold proposed by Cohen to represent a small but appreciable clinical effect.

**Table 5:** Effect sizes for primary and secondary outcomes in study completers.

Outcome Measure	Mean Change Score (SD)		P-value	Between-group Effect Size (Cohen’s d*) with 95% CI
	Yoga Group, n = 15	Control Group, n = 10		
BDI (Depression Severity)	- 9.47 (8.85)	- 1.70 (6.67)	0.020	-0.96 [-1.81, -0.12]
GSES (Self-efficacy)	2.13 (2.07)	1.5 (2.37)	0.245	0.29 [-0.52, 1.09]
RSES (Self-esteem)	2.87 (3.42)	0.20 (3.01)	0.053	0.82 [-0.01, 1.65]

* ${\text{Cohen}}^{\text{'}}\text{s d}=({\text{Change Score}}_{\text{Yoga}}-{\text{ Change Score}}_{\text{Control}})\;/{\text{ SD}}_{\text{pooled}}$ where ${\text{SD}}_{\text{pooled}}=\sqrt{\frac{\;({\text{N}}_{\text{Yoga}}\;-\;1){\left(\text{SD}\right)}_{\text{Change Score Yoga}}^{2}\;+{\;}_{\;}\;({\text{N}}_{\text{Control}}\;-\;1){\left(\text{SD}\right)}_{\text{Change Score Control}}^{2}}{{\text{N}}_{\text{Yoga}}\;+{\text{ N}}_{\text{Control}}\;-\;2}}$

Yoga appeared to exert a small, positive effect of 0.29 [95% CI, -0.52 to 1.09] in increasing GSES scores, and a large, positive effect of 0.82 [95% CI, -0.01 to 1.65] in increasing RSES scores. However, in both cases, the 95% confidence interval for the effects included zero, leaving open the possibility that any apparent effects of yoga on GSES scores and RSES scores were simply due to chance.

## Discussion

### Unique aspects of this study

To our knowledge, this is the first RCT of yoga, outside of India, characterized by both of the following features: a) all participants restricted to those with diagnosed major depression, and b) eligibility criteria explicitly excluded co-intervention with either conventional or non-conventional depression therapies. Both features are essential to an investigation of yoga as a potential mono-therapy for major depression. As described earlier, there have been an expanding number of RCTs investigating the efficacy of yoga in treating depressive symptoms. However, among such RCTs identified via published systematic reviews, there appear to be only 6 trials in which all participants were restricted to a clinical diagnosis of major depression [69–72,74,90]. Among these 6 trials, 3 were designed to assess yoga as an augmentation to anti-depressant medication [69,74,90], rather than as mono-therapy. The remaining 3 RCTs examined yoga as mono-therapy for major depression [70–72], and were all conducted in India. Thus, with our pilot RCT, the investigation of yoga as mono-therapy for major depression is extended for the first time to a western population sample.

In the United States, our pilot study also appears to be the first RCT to evaluate acute mood effects of yoga in a sample that included both men and women with a diagnosed depressive disorder—whether major depression or not. One prior RCT of yoga in the U.S. recruited a small, mixed-gender sample diagnosed with a variety of chronic depressive disorders [78]; however, in published data from this trial, depression outcomes were not reported at the time the yoga intervention ended, so acute mood effects of yoga are unknown. To date, all other RCTs of yoga involving U.S. participants with diagnosed depressive disorders have recruited only women [81,83–85,87,93], and nearly all of these single-gender RCTs recruited only peri-partum women [81,83–85,93]. Although very important to investigate yoga as a therapeutic option in depressed peri-partum women, RCTs with such selective participant samples may limit the ability to generalize findings to the larger population with depression. In sum, although our sample was small (n = 12 men, n = 26 women), this pilot study contributes important early RCT data regarding acute mood benefits of yoga in a mixed-gender U.S. population with a clinically diagnosed depressive disorder.

This pilot trial appears distinct from many previous yoga trials by providing a relatively detailed description of the yoga intervention. Well-documented protocols facilitate the replication studies essential to build on pilot investigations and eventually expand therapeutic options. We selected fairly commonplace, “open source” practices from classical yoga as components of the hatha yoga sequence, so that participants seeking to continue these yoga practices after study completion would readily find community classes, teachers, or instructional media as needed.

### Interpreting intent-to-treat analyses of depression severity

In the main intent-to-treat analysis, the yoga group exhibited greater decline in BDI scores than controls (p-value = 0.034), supporting the hypothesis that hatha yoga practice would reduce depression severity. In reviewing time-plots in Fig 2, BDI scores at baseline were statistically comparable in the two intervention groups. At 2 weeks, participation in both groups was associated with a sharp and statistically comparable decline in BDI scores; this finding suggests that our control intervention was effective in accounting for the yoga intervention’s potential non-specific mood benefit from factors such as instructor attention, peer interaction, shift from routine activities, and/or anticipation about learning novel yoga-related information. At 4 weeks and 6 weeks, BDI scores in the two groups remained statistically lower than baseline and statistically comparable to one another, suggesting an ongoing non-specific mood benefit of similar magnitude in both groups related to study participation.

BDI scores did not statistically differentiate the two intervention groups until the final 8-week measurement point, when depression scores in the yoga group were significantly lower than in the control group. One interpretation of this finding is that there may be a delay in onset of yoga-specific mood benefit, perhaps related to the time involved for participants to learn and master the yoga exercises, and the time required for psycho-physiological factors mediating specific mood benefits of yoga to develop and exert a measureable effect. Such an interpretation is plausible given that other interventions for major depression have been associated with some delay in exerting specific, measureable mood effects. For example, pharmacological interventions for major depression often have a delay of 4 weeks before exerting significant mood effects over placebo, and may take 12 weeks to achieve full anti-depressant effects [140–143]. Aerobic exercise for major depression may have a delay of at least 4 weeks before exerting significant mood effects over a comparator, and may take 16 weeks to achieve full anti-depressant effects [144–147].

Thus, to the extent that anti-depressant effects of yoga may involve some of the same mechanisms of action as pharmacotherapy or aerobic exercise, it seems consistent that hatha yoga practice may have taken 8 weeks in this trial to exert mood effects of sufficient magnitude to differentiate the yoga group from the control group. After controlling for the number of assigned intervention sessions attended by all randomized participants, those in the yoga group continued to show greater decline in BDI scores by 8 weeks (p-value = 0.045), adding support to the possibility of yoga-specific mood benefit. Since maximum anti-depressant benefit from pharmacotherapy or exercise may occur several weeks after intervention-specific effects first become appreciable, perhaps the same may be true for yoga. Future RCTs of larger sample size and longer duration may test whether any potential yoga-specific mood effects initially detected within the intervention period become stronger over time.

Several caveats in the interpretation of our data must be considered. Given our small sample size and the single time-point at which BDI scores differentiated the two groups, the statistically significant result at 8 weeks has a higher chance of being a spurious finding. For example, the difference in BDI scores at 8 weeks may not have resulted from any yoga-specific effects, but instead, from random mood variations in either group that fortuitously coincided with study hypotheses at the 8-week time-point. Thus, it is critical to undertake replication trials with larger sample sizes to gauge whether any yoga-specific mood effects may be found and reproduced reliably in studies with greater statistical power. Moreover, replication trials of longer duration, such as 16 or 24 weeks, can evaluate whether any findings of yoga-specific mood benefit persist reliably beyond just one or two measurement points. In sum, results from this trial must be viewed with caution as early, exploratory data that may support further investigation of hatha yoga in major depression, but do not offer any conclusions on its mood effects.

### Interpreting completers analyses of depression severity

Over the 8-week trial, study completers in the yoga group appeared to show greater decline in BDI scores than those in the control group (p-value = 0.014), underscoring the same group-by-time interaction observed in the full, intent-to-treat sample (p-value = 0.034). In addition, completers in the yoga group appeared to have a relatively high remission rate of 60%. However, BDI scores in completers, just as in the full sample, did not differentiate the two groups until the 8-week time-point, and the sample size of completers (n = 25) was even smaller than the full sample (n = 38). Results from the completers’ analyses are subject to the same caveats mentioned above regarding interpretation of outcomes in trials of small sample size. These pilot findings are preliminary and non-conclusive, but may serve as the basis for further investigation of this hatha yoga intervention for depression.

### Mechanisms for anti-depressant effects of yoga

The hatha yoga intervention in this study may be conceptualized as having several potential therapeutic elements—such as the basic physical activity in assuming yoga poses, the mindful way of approaching exercises with a non-judgmental attitude of observing and working with one’s limitations, the regulation of breath in specific patterns to promote alert calmness, and the deep relaxation and detachment from mental and physical activity in the final resting pose. Each of these yoga elements may comprise an “active ingredient” having its own anti-depressant mechanism(s) of action, triggering mood effects that may be additive or perhaps even synergistic in relation to effects of another active ingredient. If so, the integrated practice of several yoga elements may produce more mood benefit than any one element practiced alone. This hypothesis appears to have preliminary support from one meta-analysis [66] that examined RCTs of yoga for prenatal depression; “integrated yoga” interventions that combined yoga poses with breathing exercises, meditation or relaxation were found to be more effective in reducing depression severity than yoga poses alone. More studies are needed to clarify mood efficacy of different yoga elements, and optimal combination of elements to reduce depression.

Several mechanisms of action, both biological and psychological, have been hypothesized to underlie the anti-depressant effects of yoga practices. These effects may encompass acute, transient responses that occur during or immediately after a yoga session, as well as longer-term adaptive responses following yoga practice for several months or more. Biological mechanisms may include [64,82,148–155] down-regulation of the hypothalamic-pituitary-adrenal axis and sympathetic nervous system, increase in vagal tone with activation of the parasympathetic nervous system, increase in central nervous system neurotransmitters such as serotonin and gamma-aminobutyric acid (GABA), increase in brain-derived neurotropic factor (BDNF), promotion of cerebral alpha wave activity, and increase in telomerase activity with mitigation of depression-related cellular decline. Several psychological mechanisms have also been proposed. To the extent that yoga may offer experiences of mastery and self-acceptance, feelings of self-efficacy and self-esteem may increase [102–107] and mediate recovery from depression [108–113]. Mindfulness aspects of yoga may reduce negative ruminations in depression [87,156], leading to reduced feelings of distress [157–159]. Moreover, the decision to learn yoga may facilitate behavioral activation, a psychological mechanism by which lifestyle choices that disrupt depressive routines and lead to increased social connection or other experiences of reward become positively reinforced, thereby mediating additional adaptive behavioral change with further mood benefit [160–162]. Despite plausible support for several hypotheses, much more investigation is required on how yoga may exert mood effects.

### Study limitations and strategies for improvement

Several limitations of this study warrant further discussion. First, within the resource constraints of a pilot investigation, only a small sample size was feasible. While our study had statistically significant findings and raised the possibility of yoga-specific mood benefits, no reliable conclusions about mood effects of yoga can be drawn from such a small sample. As Button et al [163] emphasize, “a study with low statistical power…reduces the likelihood that a statistically significant result reflects a true effect.” Thus, replication studies with larger sample sizes are critical to further evaluating any potential mood effects of this hatha yoga intervention.

A second limitation was the difficulty in estimating a minimum effective “dose” of hatha yoga to produce mood effects. Yoga completers in this trial achieved a mean adherence rate of 74% to the prescribed dose of 90 minutes of yoga practice twice weekly for 8 weeks, and this intensity of practice may have produced sufficient yoga-specific mood benefit by 8 weeks to differentiate BDI scores in the two groups. However, in prior RCTs of yoga mono-therapy for major depression [70–72], yoga-specific mood benefit appeared to emerge in just 4 weeks—perhaps because yoga sessions were assigned on a daily or near-daily basis and conducted in residential or inpatient settings with nearly 100% participant adherence. If yoga-specific mood benefits of this hatha yoga sequence are confirmed in future RCTs, next steps might be to test whether increased practice intensity and/or increased adherence promote earlier manifestation of mood effects. One method to increase practice frequency (and perhaps enhance adherence) would be to augment on-site group practice with home practice, using instructional DVDs or online sessions. A trial period of 24 weeks would clarify longer-term impact on mood outcomes.

A third limitation related to the timing of outcome measures. Except for baseline measures, outcome measures were obtained from each participant immediately after he or she finished certain designated intervention sessions. While this measured the participant’s mood, self-efficacy, or self-esteem just after the completed session, it is unknown whether similar responses would have been obtained if outcome measures were done a few hours or few days later. Thus, any potential yoga-specific mood changes detected in our trial may have reflected acute effects of yoga practice that may or may not have any lasting impact. In future, this limitation might be addressed via ecological momentary assessment (EMA) technology—specifically, smartphone apps designed for frequent self-monitoring of psychological symptoms [164,165]. Traditional psychometric instruments like the BDI are not designed to measure daily mood fluctuations and are used no more than once weekly. In contrast, EMA technology allows research participants to self-assess mood at several specified intervals throughout the day, transmitting real-time outcome data that appears well-correlated with traditional psychometric scales [166–169]. In future trials, use of EMA apps, along with traditional scales like the BDI, would provide more comprehensive assessment of intervention effects from end of one session to start of the next. After trial completion, additional outcome data might be obtained from participants at 3 months, 6 months and 1 year to evaluate any longer-term effects.

Finally, another limitation of this trial was that neither participants nor intervention providers could be blinded to allocation, thereby increasing risk of performance bias. To mitigate this risk, the PI took steps (described in the Methods section) to cultivate some expectation of benefit toward both interventions, and these efforts seemed to have some success. As per time-plots in Fig 2, BDI scores in the control group appeared to match those in the yoga group quite closely for the first 6 weeks, which is unlikely to have occurred unless control participants were engaged by some expectation of benefit. Among participants (n = 30) attending at least the first allocated intervention session, education modules appeared on par with yoga in keeping participants engaged until study completion, per equivalent retention rates of 83%. Nevertheless, some residual performance bias in the two groups may have existed. Overall rates of retention and adherence in the two groups were statistically comparable, but the trend toward lower retention and adherence in the control group may have reflected expectations of lesser benefit. In particular, negative expectation very likely influenced the 6 of 18 control group participants who dropped out of the study at a very early stage, before attending even the first assigned session; this early dropout accounted for half of all attrition in the study.

We hypothesize that participants were most vulnerable to expectation-related dropout immediately after learning of their allocation to the control group. Their initial commitment to the study may have been diminished by disappointment or frustration about needing to wait several weeks before learning yoga practices, and doubts regarding the benefit of the education modules may have become heightened and influenced dropout. Assuming this hypothesis is correct, then in future, one way to mitigate early dropout and improve retention rate in the control group might be to delay disclosure of allocation until participants attend the first assigned session. In this way, even if participants are disappointed about allocation to the control group, immediate exposure to yoga-related content of the educational modules might counter negative expectations and encourage them to stay engaged in the study, as suggested by the 83% retention rate in this trial among all participants attending the first allocated intervention session. To obtain outcome data less affected by possible performance bias of participants, future trials might include a (blinded) clinician-administered depression measure, such as the Quick Inventory of Depressive Symptomatology [170], along with the participant-administered BDI.

Interestingly, several participants in the yoga group, on study completion, wanted the option to attend education modules, just as they knew participants allocated to education modules could opt for yoga classes afterwards. Participants who completed the education modules and subsequently took free yoga classes perceived benefit from both interventions, mentioning a helpful “synergy” in learning the philosophy of yoga as well as its practice. Clearly, the suggestion given to all participants at screening—that information about yoga history and philosophy might enhance later practice of hatha yoga exercises—succeeded, to some extent, in cultivating an expectation of benefit toward the control intervention. Therefore, in a future trial, we hypothesize that a crossover design may allow the expectation of benefit toward the education modules to have a more positive impact on adherence. More so than a parallel group design, a crossover design may implicitly convey to participants that both interventions may have substantive mood benefits, since both would be studied formally in every participant. This implicit message might mitigate early dropout and improve adherence in the control group.

### Future directions

Data from early RCTs have suggested potential mood benefits of yoga for a range of depressive symptoms. While these data constitute a promising evidence base from which to consider further investigation, there is insufficient evidence, as yet, to advocate yoga as a first-line treatment in any diagnosed depressive disorder. In order to advance the possibility of yoga as a therapeutic option for major depression, it is essential to undertake carefully designed studies that address methodological limitations in previous trials, and that replicate and build on earlier findings. Future studies must refine our understanding of the control interventions most suitable for ascertaining yoga-specific mood effects. Recruited participants must have well-defined depression symptomatology, so as to identify those with symptoms most amenable to yoga-based therapies. For example, a recent meta-analysis suggests that mood benefit of meditation may be greater in individuals with an acute episode of major depression, rather than chronic residual symptoms [119]. It also remains to be established whether acute mood effects of yoga suggested by short-term RCTs, such as this pilot trial, can be maintained in the longer term; thus far, one RCT [89] has provided follow-up data suggesting that a yoga intervention which acutely decreased depressive ruminations may continue to exert effects one year later.

Several populations may be particularly well served by increased research on yoga as a non-pharmacological therapeutic option for major depression. These populations include peri-partum women, adolescents, medically frail seniors, and those with medication sensitivities. While investigation of yoga has increased in peri-partum women with depression [81,83–85,93], research has been minimal in the other populations mentioned above.

Eventually, it would be helpful to ascertain comparative efficacy of yoga therapies in relation to conventional care. Perhaps most intriguing would be a direct comparison of yoga mono-therapy with conventional antidepressant medication in the treatment of acute major depression; to date, there has been only one such trial [71], and while it suggested that yoga mono-therapy may be comparable to imipramine pharmacotherapy, additional RCTs must be conducted to replicate findings. Also important is continued investigation of yoga as an adjunct to conventional care, bearing in mind that two disparate therapeutic modalities may not always be compatible, and that effects may not always be additive or synergistic. Thus far, mixed results have emerged from the few RCTs that examined yoga as an adjunct therapy to antidepressant medication for major depression [69,74,90].

As the biological and psychological mechanisms underlying mood effects of specific yoga practices are better understood, this may enable development of more “targeted” yoga therapies optimized for individuals with specific clinical presentations of major depression. For example, yoga relaxation/meditation practices that promote down-regulation of the sympathetic nervous system may prove particularly beneficial to individuals with features of anxious major depression—such as motor restlessness, insomnia, and nervous ruminations. In contrast, the vigorous practice of yoga postures and breathing techniques that increase cardiovascular and metabolic activity may prove more efficacious in individuals with melancholic features of major depression—such as excessive hopelessness, cognitive slowing, and loss of emotional reactivity. Moreover, as the neurobiology of major depression becomes better characterized, this may lead to enhanced ability to match treatment modality to individuals who are likely to experience benefit from that modality [171,172]. For example, very interesting early data [173] suggest that biomarkers may predict anti-depressant response to psychotherapy versus pharmacotherapy in individuals with major depression. Neuroimaging scans may predict an individual’s non-response to anti-depressant treatment with *either* psychotherapy or pharmacotherapy [174], raising the intriguing possibility that such individuals may be better suited for non-conventional therapeutic modalities, such as yoga. In any case, it is important to account for personal treatment preferences, as individuals with major depression may experience higher recovery rates when using therapies aligned with their personal health beliefs and expectations [175–178].

Major depression is a complex disorder, with etiology and manifestation influenced by multiple factors, including genetic vulnerability, nature of stressors, sources of support, and learned coping strategies. Therefore, it is highly unlikely that any single therapeutic modality will work uniformly well in all individuals with major depression. The goal in developing novel interventions, such as yoga, is not to replace existing conventional care that may be highly effective for some individuals, but to expand therapeutic options so that more people may benefit from treatment.

### Conclusions

This project was inspired by the need for rigorous evaluation of hatha yoga as a potential mono-therapy for individuals with mild-to-moderate major depression, a population for whom conventional care may offer uncertain benefit. With the rising popularity of yoga in western countries, yoga-based interventions with proven efficacy in major depression may provide a treatment option that is cost-effective, widely accessible, associated with high social acceptance, and has a favorable risk-benefit profile. This pilot trial suggests that in adults with major depression of mild-to-moderate severity, participation in an 8-week hatha yoga program may result in statistically and clinically significant reductions in acute depression severity. Feasibility and efficacy data from this pilot study provide support for a full-scale RCT of hatha yoga as mono-therapy for mild-to-moderate major depression in a non-hospitalized, metropolitan U.S. population sample. If anti-depressant efficacy of hatha yoga were validated in larger scale RCTs, this would be of enormous relevance to public health.

## Supporting information

S1 File IRB-approved study protocol. (PDF)

S2 File CONSORT checklist. (PDF)

S3 File NIMH poster session. (PDF)

S4 File Educational modules on yoga history and philosophy. (PDF)

S1 Dataset Demographic and clinical characteristics of randomized participants. (XLSX)

S2 Dataset Primary and secondary outcomes at each measurement point. (XLSX)
