# Supplementary material for: Treating major depression with yoga: A prospective, randomized, controlled pilot trial
Source: PLoS One. 2017 Mar 16;12(3):e0173869. doi: 10.1371/journal.pone.0173869 (PMC5354384; doi:10.1371/journal.pone.0173869)
Supplement: S1 File — (PDF) [file pone.0173869.s001.pdf]

COMMITTEE ON HUMAN RESEARCH
OFFICE OF RESEARCH, Box 0962
UNIVERSITY OF CALIFORNIA, SAN FRANCISCO
www.research.ucsf.edu/chr/Apply/chrApprovalCond.asp
chr@ucsf.edu
(415) 476-1814

CHR APPROVAL LETTER

TO: Sudha Prathikanti, M.D.
Box 1726

RE: Treating Depression with Yoga: A Pilot Study with Adults and Adolescents

The Committee on Human Research (CHR) has reviewed and approved this application to involve humans as research subjects. This included a review of all documents attached to the original copy of this letter.

Specifically, the review included but was not limited to the following documents:
Consent Form, Dated 4/20/10

The CHR is the Institutional Review Board (IRB) for UCSF and its affiliates. UCSF holds Office of Human Research Protections Federalwide Assurance number FWA00000068. See the CHR website for a list of other applicable FWA's.

APPROVAL NUMBER: H49362-35940-01. This number is a UCSF CHR number and should be used on all correspondence, consent forms and patient charts as appropriate.

APPROVAL DATE: April 29, 2010 **EXPIRATION DATE:** April 6, 2011 **Full Committee Review**

GENERAL CONDITIONS OF APPROVAL: Please refer to www.research.ucsf.edu/chr/Apply/chrApprovalCond.asp for a description of the general conditions of CHR approval. In particular, the study must be renewed by the expiration date if work is to continue. Also, prior CHR approval is required before implementing any changes in the consent documents or any changes in the protocol unless those changes are required urgently for the safety of the subjects.

HIPAA "Privacy Rule" (45CFR164): This study requires individual consent/authorization for use and/or disclosure of Protected Health Information (PHI).

Sincerely,

Diane W. Wara

Diane W. Wara, M.D.
Vice Chair, Committee on Human Research

cc:

UCSF
COMMITTEE ON HUMAN RESEARCH
FULL COMMITTEE REVIEW APPLICATION

Please date form: 4/20/10

Is this a NIH "Just in Time" submission? **[ ]Yes [X]No**

If also applying to the **Clinical and Translational Science Institute (CTSI) at UCSF**, please submit one copy of this application to the CTSI Clinical Research Center where your study will take place. Visit http://ctsi.ucsf.edu/index.html, http://gcrcsfgh.ucsf.edu/, or http://www.gcrc.ucsf.edu/ for additional instructions regarding the CTSI application process.

General Instructions | Submission Checklist

Street Address:
Committee on Human Research (CHR)
Office of Research
3333 California Street, Suite 315
University of California
San Francisco, CA 94118

Campus Mailbox:
CHR
Box 0962

Office Contact for questions:
Office: (415) 476-1814
Facsimile: (415) 502-1347
e-mail: chr@ucsf.edu

PART 1: ADMINISTRATIVE REQUIREMENTS

- Eligibility requirements for Principal Investigator, Co-Principal Investigator and Contact Person
- Training requirement

**A. Principal Investigator:**		
Name and degree<br>Sudha Prathikanti, MD	University Title<br>Assoc Clinical Professor	Department<br>Psychiatry
Campus Mailing Address (Box No.)<br>Box 1726, Osher Ctr for Integrative Med	Phone Number<br>(415) 353-7720	E-mail Address<br>Sudha.Prathikanti@ucsf.edu
Co-Principal Investigator:		
Name and degree	University Title	Department
Campus Mailing Address (Box No.)	Phone Number	E-mail Address
Additional Contact Person (if any):		
Name	University Title	Department
Campus Mailing Address (Box No.)	Phone Number	E-mail Address
Send correspondence to (check *one*): [X]PI only	[ ]PI and Co-PI	[ ]PI and Additional Contact Person

Study Title:	Application Type:
Treating Depression with Yoga:<br>A Pilot Study with Adults and Adolescents.	[ ]New Full Committee Application<br>[pX]Response to "Contingent" or "Return" letter<br>[ ]Modification [ ]Renewal<br>Current CHR #: __<br>Expiration date: __

UCSF Sites (Check all that apply):

[X]UCSF [ ]Cancer Center [X] Mt. Zion [ ]SFGH [ ]ITN [ ]Fresno

CTSI CRC Sites (Check all that apply):

[ ]Moffitt Inpatient Unit	[ ]SFGH Inpatient Unit	[ ]Moffitt Pediatric Inpatient Unit	[ ]Kaiser Oakland DOR
[ ]Moffitt Outpatient Unit	[ ]SFGH Outpatient Unit	[ ]Moffitt Pediatric Outpatient Unit	[ ]CHORI Pediatric
[ ]Mt. Zion Outpatient unit	[ ]VAMC Outpatient Unit	[ ]Pediatric Critical Care Units	[ ]CHORI Adult
[ ]Tenderloin Medical Center			

UCSF Affiliated Sites (Check all that apply):

[ ]VAMC [ ]Gladstone [ ]Gallo [ ]SFDPH [ ]IOA [ ]BSRI [ ]BCP

**UC Campus** – Read the guidance to rely on another UC Campus IRB and submit the correct Notice of Intent to Rely form.			
[ ]UC Berkeley	[ ]UC Irvine	[ ]UC Riverside	[ ]UC Santa Cruz
[ ]UC Davis	[ ]UC Los Angeles	[ ]UC San Diego	
[ ]Lawrence Berkeley National Laboratory	[ ]UC Merced	[ ]UC Santa Barbara	
**Non-UCSF Affiliated Sites - Attach IRB Approval Certification Supplement for all sites checked below:**			
[ ]Foreign Country	[ ]Other Institution: ___________		
[ ]Other Community-Based Site: ________			

B. Funding: If this study is eligible for "Just in Time" NIH review, do not submit your application to the CHR until you have received notification from the federal granting agency that your study appears to be in a fundable range. If this study is federally funded please complete section B.6.
Check all that apply:

**1. Type of funding:**	**2. Source of funding:**	**3. Funds will be awarded to/through:**
[X]Contract/Grant<br>[ ]Subcontract<br>[ ]Drug/device donation<br>[ ]Departmental<br>[ ]Gift<br>[ ]Student project<br>[ ]Other: __<br><br>Have funds been awarded?<br>[X]Yes [ ]Pending [ ]No<br><br>Award Numbers:<br>1) 435299-86871-01 and<br>2) 556501-41743)<br>**Proposal Express** number(s): __	[ ]Federal Government<br>[ ]Other Gov. (e.g., State, local)<br>[ ]Industry<br>[X]Other Private<br>[ ]Campus/UC-Wide program<br>[ ]Departmental Funds<br>[ ]Other:<br><br>**Specify name of source designated above:** __<br><br>1) Mental Insight Foundation<br>2) The Pritzker Fund	Dept./ORU:<br>*Institution* — *Federal Wide Assurance (FWA) No.*<br>[X]UCSF .... 00000068<br>[ ]Blood Centers of the Pacific .... 00002111<br>[ ]Blood Systems Research Institute .... 00006454<br>[ ]Gallo Institute .... 00000304<br>[ ]Gladstone Institute .... 00000087<br>[ ]Institute on Aging .... 00002525<br>[ ]NCIRE .... 00000256<br>[ ]S.F. Dept. of Public Health .... 00000162<br>[ ]SFVAMC Research Office 00000280

**4.** UCSF (or affiliate) financial contact person for IRB review recharge:	Liana Hartano 415-353-7715
**5.** Grant Title and PI (if different from above):	

6. CHR Protocol/Federal Grant or Contract Comparison (New CHR Studies Only)
If this study is federally funded, please submit one copy of one of the following documents (unless there is more than one grant or contract involved; in that case, submit one copy for each associated grant or contract). Please indicate which document you have attached:

[ ] The Research Plan, including the Human Subjects, Section E of your NIH grant
[ ] For other federal proposals (contracts or grants), the section of the proposal describing human subjects work, or
[ ] The section of your progress report if it provides the most current information about your human subjects work.

7. If there are any significant discrepancies between this CHR application and the grant or contract or if this is a training grant please explain here:

8. Secondary sponsors: If there are multiple sources of funding for this study, please describe the additional funding:

C. Scientific or Scholarly Review:

Is this an investigator-initiated study? [X]Yes [ ]No

This study has received scientific or scholarly review from (check all that apply):
The results of this review must be communicated to the CHR as part of the CHR review process.

[ ]NIH* [ ]Cancer Center** [ ]CTSI CRC [ ]SFVAMC [ ]CHORI [ ]Kaiser Oakland DOR [ ]GESCR
[ ]Departmental Review*** [ ]Other _

* Specific to this study.
** Required prior to final CHR approval for oncology studies.
*** Submit a copy of the signed Departmental Scientific Review form.

D. Key Personnel: All key personnel including the PI and Co-PI must be listed below along with a brief statement of their qualifications and study role(s). *If the SF VAMC is a study site*, please identify the principal VAMC investigator, unless already listed as PI or Co-PI above. For questions regarding the VAMC application process, please contact the VA Clinical Research Office at 221-4810 ext.4655. ***Please note:*** All Key Personnel at UCSF or affiliated sites must complete the UCSF Collaborative Institutional Training Initiative (CITI) online module

Investigators and other personnel [and institution(s)]:	Qualifications:	Study role(s):
Sudha Prathikanti, MD	- Associate Clinical Professor, UCSF Dept of Psychiatry<br>- Experience as co-investigator and study physician for NIDA/NIAAA R01 clinical trials<br>- Experience with integrative medicine interventions as clinical faculty member at UCSF Osher Ctr for Integrative Medicine	Principal Investigator
Renee Rivera, RN, CYT	- Registered Nurse and Certified Yoga Therapist/ Instructor<br>- Experience as SFGH public health nurse for 20 years	Yoga instructor
Michael Acree, PhD	- Biostatistics Director, UCSF Osher Ctr for Integrative Medicine<br>- Experience with data analyses of clinical trials	Randomization & Data analysis

**E. Statement of Financial Interest:** Does the PI or any investigator have any financial interests related to this clinical study?<br>**If Yes, Attach Disclosure Of Investigators' Financial Interests Supplement**	[ ]Yes [X]No

F. Drugs, Devices and Biologics:

List any drugs and/or biologics under investigation, and IND status*:	name	IND#*, "pending" or "exempt"
List any devices under investigation and IDE status*:	name	IDE#*, "pending" or "exempt"
	[ ] Non-Significant Risk Determination Requested	**Attach - NSR Supplement**

* Verification of IND/IDE numbers: If the sponsor's protocol does not list the IND/IDE number, your application must include documentation from the sponsor or FDA identifying the IND/IDE number for this study.

Who holds the IND/IDE?	[ ]Sponsor [ ]Investigator:**

** Investigators who hold an IND/IDE are responsible for knowing and following FDA regulatory requirements for sponsors. See HRPP Guidance on Investigational New Drugs and Biologics or Investigational Devices. Consultation and advice are available through CTSI RKS, Regulatory Knowledge and Support Service.

Are investigational drugs or biologics controlled by a pharmacy? Contact information for investigational drug pharmacists is in HRPP IND guidance.	[ ]Yes [ ]No<br>If "Yes," identify the pharmacy/ies:<br>If "No," describe your plan for control of the test article:
Are investigational drugs, devices, or biologics (test articles) controlled by the Principal Investigator?<br>See IDE Guidance.	[ ]Yes [ ]No<br>If "Yes," describe your plan for control of the test article:
Are investigational drugs, devices, or biologics prepared or manufactured in UCSF research labs?	[ ]Yes [ ]No<br>If "Yes," identify the lab:

**G. Other Approvals/Regulated Materials:** Does this study require approval or authorization from any of the following regulatory committees, or involve the use of the regulated materials listed below? Follow the hyperlinks for more information. If "Yes," complete the applicable section(s) below.			[ ]Yes [X]No
[ ]	Biological Safety Committee		BUA #:
	[ ]	Human Gene Transfer/Recombinant DNA Research	**Attach - Human Gene Transfer / Recombinant DNA Research Supplement**
[ ]	Institutional Animal Care and Use Committee		IACUC #:
	[ ]	Xenotransplantation	
[ ]	Controlled Substances		
[ ]	Human Stem Cells		**Attach - Human Stem Cell Supplement**
[ ]	Radiation Safety Committee		RUA #:

H. Clinical Trial Registration:

Public Law 110-85 requires registration of clinical trials. The International Committee of Medical Journal Editors (ICMJE) also requires registration of clinical trials in order for results to be published in member biomedical journals. Additional information, including guidance on the UCSF registration process for ClinicalTrials.gov at UCSF, and the *definition of a clinical trial* for purposes of registration can be found at Office of Research News, Vol. 8, No. 2.

Please provide one of the following:

ClinicalTrials.gov "NCT" number for this trial: ___________, or: [X] Registration pending
[ ]This is not a clinical trial; registration not required

Clinical trials are required to be registered *before the enrollment of the first participant*, but not prior to CHR approval.

I. Principal Investigator's Certification:

- I certify that the information provided in this application is complete and correct.
- I accept ultimate responsibility for the conduct of this study, the ethical performance of the project, and the protection of the rights and welfare of the human subjects who are directly or indirectly involved in this project.
- I will comply with all policies and guidelines of UCSF and affiliated institutions where this study will be conducted, as well as with all applicable federal, state and local laws regarding the protection of human subjects in research.
- I will ensure that personnel performing this study are qualified, appropriately trained and will adhere to the provisions of the CHR-approved protocol.
- I will not modify this CHR-certified protocol or any attached materials without first obtaining CHR approval for an amendment to the previously approved protocol.
- I assure that the protected health information requested, if any, is the minimum necessary to meet the research objectives.
- I assure that the protected health information I obtain, if any, as part of this research will not be reused or disclosed to any parties other than those described in the CHR-approved protocol, except as required by law.
- I assure that adequate resources to protect participants (i.e., personnel, funding, time, equipment and space) are in place *before* implementing the research project, and that the research will *stop* if adequate resources become unavailable.

_______________________________________ ____________________

Principal Investigator's Signature Date

PART 2: STUDY DESIGN

Complete items A-E using clear, concise, non-technical, lay language (i.e., the type of language used in a newspaper article for the general public) wherever possible. Define all acronyms. Use caution when cutting and pasting from another application or protocol to ensure that information is complete, supplemented where necessary, is pasted in a logical order, and is relevant to the specific section. Space limits are recommendations and should be adjusted as needed, but the total length for sections A-E should not exceed 5 pages. For modifications and renewals, please highlight in *italics* all changes from previously approved version.

A. Synopsis (Briefly summarize the study.) Space limit: quarter page

This will be a single-center, single-blind, randomized, controlled, parallel group, pilot trial involving 40 participants (20 adults and 20 adolescents) with mild to moderate depression, as determined by structured psychiatric interview and by scores ranging from 14 to 28 on the Beck Depression Inventory (BDI-II). The purpose of this pilot trial will be to provide empirical data regarding the potential efficacy of yoga as an intervention for depression. This pilot trial will also provide feasibility data and estimation of effect size for a full-scale, larger, randomized, controlled trial of yoga for depression.

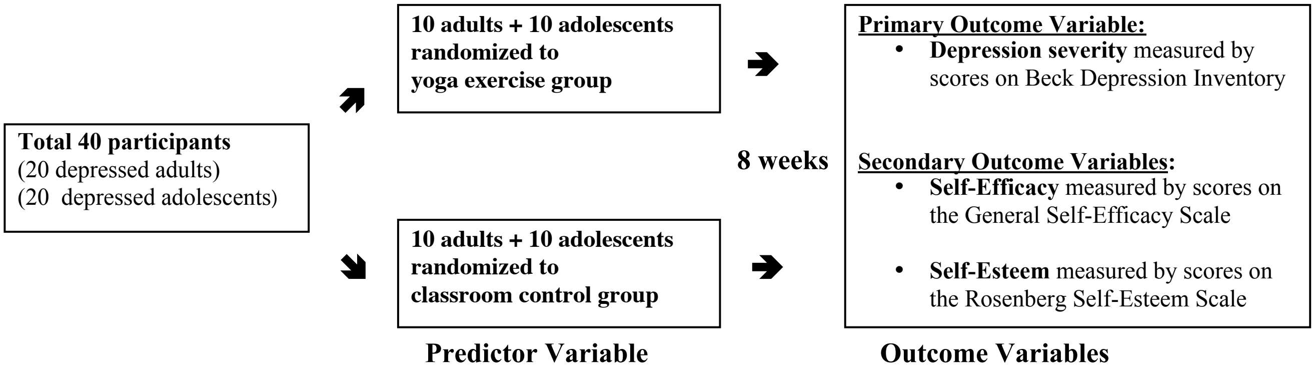

Individuals with mild to moderate depression choosing to enroll in this clinical trial will be opting for participation in an 8-week, yoga-based intervention for depression, rather than obtaining conventional mental health care during this period. Individuals with severe depression (per BDI-II scores greater than 28) will be excluded from this study. Individuals meeting all eligibility criteria will be randomized equally to one of two intervention groups: an active intervention group assigned to practice yoga exercises, versus a classroom control group assigned to attend a yoga philosophy course. Participants in both interventions groups will meet for 90-minute sessions 2 times per week for 8 weeks. Participants will undergo stratified block randomization to ensure that each intervention group has equal numbers of participants with mild depression (per BDI-II scores of 14 to 19) versus participants with moderate depression (per BDI-II scores of 20 to 28). Upon completion of the assigned study intervention, any participant randomized to the classroom control group will have the option of attending free yoga sessions at UCSF to learn and practice the yoga exercises used in the active intervention.

The primary outcome measure will be depression severity, as measured by BDI-II scores; the BDI-II will be administered at baseline, and again at 2 weeks, 4 weeks, 6 weeks, and 8 weeks. Secondary outcome measures will be participant self-efficacy and self-esteem, as measured by the General Self-Efficacy Scale (GSES) and the Rosenberg Self-Esteem Scale (RSES), respectively; the GSES and the RSES will be administered at baseline and again at 8 weeks. At the end of the study, blinded outcome assessors will evaluate whether participants in the yoga exercise group, in comparison to those in the classroom control group, achieve the following: (a) statistically significant reduction in depression severity, as measured by scores on the BDI-II, and (b) statistically significant increase in self-efficacy and self-esteem, as measured by scores on the GSES and the RSES, respectively. Investigators will also assess characteristics of all participants responding to recruitment efforts, and determine recruitment response rates, as well as rates of enrollment, adherence and attrition.

All participants will undergo continuous, close clinical monitoring for safety throughout this trial. The study PI is an experienced psychiatrist and will evaluate participants for immediate withdrawal from the study and for referral to appropriate psychiatric care should *any* of the following occur: a participant reports subjective worsening of depression at any point in the study; a participant develops suicidal ideation or psychosis at any point in the study; there is a clinically significant increase (4 points or more) between a participant's baseline BDI-II depression severity score and the BDI-II score at any subsequent assessment interval in the study.

B. Hypothesis(es): Briefly explain the hypothesis(es) to be tested. If the study is not designed to test a hypothesis, simply state "None."

Primary Hypothesis:

1) Compared to depressed participants randomized to the classroom control group, depressed participants randomized to the yoga exercise group will achieve a statistically significant reduction in depression severity, as measured by scores on the Beck Depression Inventory (BDI-II).

Secondary Hypotheses:

2) Compared to depressed participants randomized to the classroom control group, depressed participants randomized to the yoga exercise group will achieve a statistically significant increase in self-efficacy, as measured by scores on the General Self-Efficacy Scale (GSES).

3) Compared to depressed participants randomized to the classroom control group, depressed participants randomized to the yoga exercise group will achieve a statistically significant increase in self-esteem as measured by scores on the Rosenberg Self-Esteem Scale (RSES).

C. Specific Aims: List the specific aims.

Aim 1: Test the hypothesis that in comparison to depressed participants randomized to the classroom control group, depressed participants randomized to the yoga exercise group will achieve a statistically significant reduction in depression severity, as measured by scores on the Beck Depression Inventory (BDI-II).

Aim 2: Test the hypothesis that in comparison to depressed participants randomized to the classroom control group, depressed participants randomized to the yoga exercise group will achieve a statistically significant increase in self-efficacy, as measured by scores on the General Self-Efficacy Scale (GSES).

Aim 3: Test the hypothesis that in comparison to depressed participants randomized to the classroom control group, depressed participants randomized to the yoga exercise group will achieve a statistically significant increase in self-esteem, as measured by scores on the Rosenberg Self-Esteem Scale (RSES).

Aim 4: Evaluate participant characteristics, recruitment response rate, enrollment rate, adherence rate and attrition rate in a randomized controlled trial of yoga for depressed adults and adolescents in the United States.

D. Background and Significance: Briefly sketch the scientific background leading to the present proposal, critically evaluate existing knowledge (with references), and specifically identify the gaps the project is intended to fill. State concisely the importance and health relevance of the research described in this application by relating the specific aims to the broad, long-term objectives.

The World Health Organization identifies major depression as the single most disabling illness in the United States, accounting for more days of disability each year than heart disease, hypertension, diabetes or chronic back pain.[1-2] Major depression contributes not only to disability, but also to mortality. There are more than 29,000 deaths by suicide annually in the United States,[3] and it is estimated that major depression accounts for 20-35% of these deaths.[4] Major depression also increases the risk of death from medical conditions such as coronary artery disease[5,6] and diabetes mellitus.[7,8]

In both adults and adolescents, conventional treatments for major depression include several classes of antidepressant medication and a variety of psychological interventions such cognitive-behavioral therapy, interpersonal therapy, supportive therapy and group therapy. Unfortunately, these conventional treatments for depression have significant drawbacks: both pharmacological and psychological treatments are limited by low remission rates [9-16] ranging from 25-45%, and high dropout rates [17-22] approaching 50% due to cost, protracted duration of treatment, and/or social stigma. Pharmacological and psychological interventions for depression each have additional modality-specific limitations:

- Pharmacological treatments often are associated with significant side effects, which may range from life-threatening cardiac arrhythmias or hypertensive crises to less dire, but often intolerable, problems such as sexual dysfunction, weight gain, blurry vision, headaches, agitation or insomnia. The safety of pharmacological treatment has been particularly questioned in pediatric and adolescent depression; in 2005, the FDA issued a black box warning on all anti-depressant medication use in individuals under the age of 18 yrs, citing meta-analyses[23] that described an increased risk of suicide associated with antidepressant use in this age group. In 2007, the FDA black box warning was extended[24] to include all anti-depressant medication use in individuals under the age of 25 yrs.

- With psychological treatments, potential mood benefits may be strongly limited by the expertise and individual characteristics of the therapist, rather than the specific course of psychotherapy provided.[25-27] There is significant risk of premature discontinuation of psychotherapy due to inadequate/problematic alliance with the therapist or cultural dissonance with the psychotherapeutic process.[28-33]

Some data[34-36] suggest that the combination of pharmacotherapy and psychotherapy may yield higher remission rates and lower relapse rates than either modality alone, especially in severe, recurrent, or chronic depression. However, in treating most adults with depression, there is no strong empirical basis[37] to recommend routine treatment with both modalities, and such combination therapy may be cost-prohibitive for many. In adolescents, efficacy data regarding the combination of pharmacotherapy and psychotherapy in acute depression have been ambiguous. Analyses from the large, multi-center Treatment for Adolescents with Depression Study (TADS) indicate that mono-therapy with cognitive behavioral therapy does not produce significantly better results than placebo medication, but suggest that combination therapy with fluoxetine and cognitive behavioral therapy is superior to placebo, and superior to mono-therapy with either fluoxetine or cognitive behavioral therapy alone.[38] However, in another large clinical study, the Adolescent Depression and Psychotherapy Trial (ADAPT), the findings demonstrate no difference in treatment effectiveness of fluoxetine plus cognitive behavioral therapy over fluoxetine alone.[39] In a third large clinical trial entitled the Treatment of Resistant Depression in Adolescence (TORDIA), depressed adolescents unresponsive to a first trial of medication mono-therapy benefit more from the subsequent combination of cognitive behavioral therapy and a medication change than from a medication change alone.[40]

Given the limitations of conventional therapies for depression, as well as ongoing public health challenges in delivering appropriate care to depression sufferers,[41-43] it is not surprising that the general public in the United States has shown growing interest in the use of complementary and alternative therapies, such as yoga, in the treatment of depressive disorders.[44-47] With the rising popularity of yoga in western countries,[48] yoga-based interventions hold the potential for offering a non-conventional form of depression treatment associated with low cost, high accessibility, high social acceptance, and few side effects. Additionally, gentle yoga interventions may be practiced by individuals with medical conditions that may prevent more rigorous physical exertion. However, randomized controlled trials examining antidepressant effects of yoga are extremely few in number. In a recent review,[49] investigators identified 5 randomized controlled trials over the past two decades which have examined yoga as the primary intervention for depression:

1) **Woolery et al. (2004)** [50] recruited 28 subjects who completed the Beck Depression Inventory, and scored in the range suggestive of mild depression; subjects were randomized to one of two interventions: Iyengar yoga course with back bends/standing poses/inversions or a wait-list control. The interventions were carried out over 5 weeks, with the yoga group practicing in 60-minute sessions twice weekly. Attrition rate was 18% and adherence rate was not reported. **Results:** Compared to wait-listed subjects, those in the yoga group showing significant reduction in scores on the Beck Depression Inventory. **Study Limitations:** Lack of an accepted method for diagnosing depression in subjects; lack of active control group; small sample size; short duration of trial; lack of information about randomization methods/blinding of assessors/adherence rates.

2) **Janakiramaiah et al. (2000)** [51] undertook a controlled open trial in 45 hospitalized but medication-free subjects with a DSM-IV diagnosis of major depressive disorder with melancholic features. Subjects were randomly but equally assigned to receive ECT, imipramine or Sudarshan Kriya Yoga (SKY) for 4 weeks. Bilateral ECT on alternate days was used under anesthesia; ECT was withheld if patients reached a HRSD score less than 8 on two consecutive weekly assessments. Imipramine was used in a fixed dose of 150 mg per day orally at bedtime. SKY was practiced under supervision in the hospital for 45 minutes a day, six days a week. Depression severity was assessed via Hamilton Rating Scale for Depression (HRSD) before starting treatment and weekly thereafter for four weeks; remission was defined as a HRSD score less than 8. There were no dropouts, and the average adherence rate for SKY was 84%. **Results:** Per HRSD scores, the ECT group showed a 93% remission rate, but the imipramine and SKY groups were comparable to one another with respective remission rates of 73% and 67%. **Study Limitations:** Unblinded trial with potential for rater bias; small sample size; short duration of trial; active control, but no sham/placebo control group against which to compare effect size in each intervention group.

3) **Rohini et al. (2000)** [52] recruited 30 inpatients having a DSM-IV diagnosis of major depressive disorder. Subjects were randomized into two equal groups for 4 weeks. One group practiced SKY for 45 minutes a day, six days a week. The other group received 'partial' SKY; this latter was similar to SKY except that the longest sequence of yoga breathing exercises was replaced by normal breathing. Depression symptoms were assessed with Beck Depression Inventory. Response was defined as 50% reduction in baseline Beck depression scores. Subjects and questionnaire administrators were blind to treatment assignments. There were no dropouts and adherence rates to both full and partial SKY were

greater than 80%. **Results:** At the end of 4 weeks, both groups showed significant reduction in depression scores, with an 80% response rate in the full SKY group and 58% response rate in the partial SKY group; the difference in response rate narrowly missed statistical significance. **Study Limitations:** Small sample size; short duration of trial; control intervention had significant overlap with the SKY intervention.

4) **Khumar et al. (1993)** [53] recruited 50 outpatients diagnosed with severe depression per clinical interview and Zung Depression Self Rating Scale. Subjects were randomized to one of two interventions: Shavasana yoga pose with rhythmic breathing or no intervention. Those in the Shavasana group practiced for 30-minute each day for 30 days. Neither attrition nor adherence rates were reported. **Results:** Compared to subjects in the no intervention group, subjects in the Shavasana group showed significant reduction in scores on the Zung Depression Self Rating Scale. **Study Limitations:** Small sample size; short duration of trial; lack of active control group; lack of information re: cutoff scores for determining severe depression; lack of information about randomization methods/ blinding of assessors/attrition and adherence rates.

5) **Broota and Dhir (1990)** [54] recruited 30 outpatients with clinically diagnosed depression and randomized them to one of 3 interventions: yoga-based relaxation involving deep breathing/spine stretching/leg raising, Jacobsen's Progressive Relaxation, or a control intervention in which subjects narrated their states of mind. The interventions occurred in 20-minute sessions on 3 consecutive days. Attrition rates were low (10%) in each group; adherence rates were not reported. **Results:** Compared to subjects in the control group, subjects in both relaxation groups reported significant reduction in depression symptoms, as measured by a self-administered symptom checklist. **Study Limitations:** The use of a non-validated instrument as the main outcome measure; small sample size; very short duration of trial; lack of information about randomization methods/concealment of allocation/blinding of assessors/adherence rates.

While all 5 clinical trials above suggested significant mood benefits from yoga, the studies tended to be hampered by methodological concerns. Four of the five trials were conducted in India, raising questions as to whether study results obtained in an Indian population might differ significantly from those obtained in a western population due to differences in cultural expectations. None of the trials examined yoga as an intervention for adolescent depression, a condition for which mono-therapy with psychotherapy may be of limited benefit,[38] while pharmacotherapy may increase suicide risk.[23] Two trials [50,53] provided inadequate information regarding the methods for diagnosing depression in subjects, and also lacked an active control group. Other limitations include small sample size, short duration of trial (3 days to 5 weeks), and missing information re: key procedures such as methods for randomization, for concealment of allocation, and for blinding of assessors. Finally, none of these trials attempted to correlate depression outcomes with the psychological variables of self-efficacy and self-esteem, despite existing data suggesting that self-efficacy and self-esteem tend to improve with physical exercise and movement[55-59] and may help to mediate mood benefits of exercise interventions.[60-63]

In this proposed research project, the total sample size of 40 participants remains relatively small, given that this is pilot study intended to provide data as to whether a larger-scale study is warranted. However, as outlined below, we aim to eliminate other methodological limitations described in previous yoga studies:

- This study will be among the first randomized, controlled trials to examine yoga as an intervention for a depressed U.S. population, helping address the question of whether mood benefits of yoga can be replicated outside India.
- This study will include *both* adults and adolescents with depression to assess for potential mood benefits of yoga.
- Participants will be diagnosed with depression using a validated method (Mini Int'l Neuropsychiatric Interview).
- PI will ensure that appropriate randomization methods and blinding of assessors will be implemented and documented in this clinical trial.
- Study design will include an intervention period of 8 weeks, considerably longer than previous yoga trials, to better assess mood effects over time and capacity of depressed participants to adhere to an exercise intervention.
- Study design will include an active classroom control group, controlling for non-specific mood effects potentially associated with the yoga exercise group (e.g. instructor attention, peer interaction, shift from usual routine).
- Primary outcome of depression severity will be measured by a validated instrument (Beck Depression Inventory).
- This trial will examine how self-efficacy and self-esteem, as measured by validated instruments (the General Self-Efficacy Scale and the Rosenberg Self-Esteem Scale) may correlate with changes in depression severity.

If this pilot trial validates antidepressant effects of yoga, it would provide justification and valuable feasibility data for investigators at UCSF, as well as at other academic centers, to pursue larger-scale, high-quality studies of yoga interventions for depressed U.S. populations. If antidepressant effects of yoga continue to be validated through such larger-scale trials, a non-conventional treatment for major depression may emerge that is associated with wide accessibility and little expense, social stigma or risk of side effects; clearly, this would be of enormous relevance to public health.

E. Preliminary Studies: Preliminary data often aid reviewers in assessing how valuable the project is likely to be. If graphs or tables are used to convey information, please maintain a consistent style and make sure that fonts are no less than 11-point in size. If no preliminary data are available, it may be helpful to briefly indicate why this proposed study is a reasonable starting point.

The PI of the proposed research project previously helped conduct a small, open-label UCSF pilot trial of yoga for depression.[64] As summarized below, this trial recruited 12 participants diagnosed with major depression via psychiatric interview; all participants had baseline scores of 14 or greater on the Hamilton Rating Scale for Depression (HRSD) and on the Beck Depression Inventory (BDI). Participants attended a 60-minute yoga class once weekly at UCSF for 4 weeks and were also instructed to practice yoga breathing exercises at home for 30 minutes daily during this same period. Participants continued any pre-existing care with psychotherapy or psychotropic medication, but had to be stable on conventional treatment for at least 2 months prior to study enrollment. During the study, the attrition rate was 25%. Adherence rates for the group sessions and home practice sessions were 68% and 63%, respectively. At the end of 4 wks, participants exhibited clinically significant reductions in HRSD scores (p value=.03) as well as in BDI scores (p value=.01). The majority of participants were willing to continue the study for an additional 4 wks if given the opportunity, and to attend more group practices per week. Results from this open-label trial helped to refine our methodology for the current research project.

Treating Depression with Yoga Breathing: An open-label feasibility trial conducted Aug/Sept 2005 (Unpublished data; Prathikanti, Wolkowitz and Fernandes, 2005)

**Aims**	Assess recruitment response rate & enrollment rate of U.S. subjects in a study of yoga breathing for depression<br>Assess attrition & adherence rates of depressed U.S. subjects in a time-intensive pranayama intervention<br>Assess whether subjects practicing pranayama exhibit reduction in depressive symptoms
**Subjects**	Adults 18 and older interested in learning a yoga practice that may help reduce depressive symptoms<br>Consecutive sampling of outpatients recruited thru two UCSF outpatient clinics via flyers<br>Major depression diagnosed with Mini International Neuropsychiatric Interview<br>Baseline HRSD score >13 , baseline BDI score >13<br>Patients either not taking antidepressant medication or<br>taking stable dose of antidepressant medication for > 2 months<br>No participation in any other yoga practice during the study<br>No acute psychosis or suicidal ideation
**Intervention**	Daily 30-minute home practice of 3 yoga breathing exercises (with audiotape) &<br>Weekly 60-minute instructor-led group practice of 3 yoga breathing exercises
**Duration**	Total duration of study: 4 weeks
**Outcome Measures**	Total number of people responding to recruitment ads over a one month period<br>Total number of subjects enrolled in the study over a one month period,<br>Percentage of assigned exercise sessions completed by subjects,<br>Change in mean score on Beck Depression Inventory (BDI) pre-and post-intervention<br>Change in mean score on Hamilton Rating Scale for Depression (HRSD) pre-and post-intervention
**Results**	Recruitment response rate: 42 subjects/month<br>Enrollment rate: 12 subjects/month (9 were taking antidepressants)<br>Total Attrition rate: 3 subjects (25%)<br>Total Group Practice sessions completed: 68%<br>Total Home Practice sessions completed: 63% (home data was missing on 3 subjects)<br><br>At conclusion of study, 75% of subjects indicated willingness to attend 3 group practices/wk at research site

	**Pre-Intervention N=12**	**Post-intervention N=10**
Mean BDI	28.67	17.90
SD	12.61	13.82
SEM	3.64	4.37

Change in BDI scores = 11.10 (intent to treat analysis)
95% confidence interval for change= 1.41 - 20.79;
P value = 0.0292

	**Pre-Intervention N=12**	**Post-Intervention N=9**
Mean HRSD	25.25	13.67
SD	6.00	6.40
SEM	1.73	2.13

Change in HRSD score = 11.22 (intent to treat analysis)
95% confidence interval for change= 4.46 - 17.99
P value= 0.0050

F. Design

1. (Check all that apply):

[X]Randomized [X]Blinded (single) [ ]Investigational intervention without random assignment [X]Behavioral

If this study has any of the formal designations below, please indicate:
[ ]Phase I [X]Phase II [ ]Phase III [ ]Phase IV
[ ]Open Label Extension: If so, specify CHR Approval Number for original study: __

[ ] Multicenter: If so, is the UCSF PI the lead investigator, coordinating center or the prime grant holder? If yes, please address how the following information will be recorded and shared among sites:	[ ]Yes [X]No

a. How will safety updates, interim results, or other information that may impact risks to the participant or others be communicated among sites?

b. How will any modification(s) to the protocol or consent document(s) be shared among sites?

Note: It is the responsibility of the coordinating center and/or prime grant holder to maintain the IRB Approvals for all study sites.

2. Community Engagement – The following questions are designed to gather information about the extent to which UCSF researchers are seeking to create or have created ongoing clinical and translational research partnerships with community members. *A community-based clinical and translational research partnership is defined as a research partnership that actively engages community participants in aspects of the research that extend beyond volunteering to be a participant in a clinical trial.*

**a. Community-Based Research:** Does this project involve the development or continuation of community-based research partnerships between UCSF and local members of community-based advocacy groups; religious, ethnic, or neighborhood organizations; schools; local government agencies; local businesses; or other local organizations?	[ ]Yes [X]No
**b. Practice-Based Research:** Does this project involve the development or continuation of research partnerships between UCSF and local community-based clinicians (such as dentists, nurses, pharmacists, physicians, or other health professionals), organizations that provide healthcare (such as local clinics, medical groups, pharmacies, or insurance providers), or other health-care organizations (such as professional membership societies)?	[ ]Yes [X]No

Note: if other sites are engaged in the research they must be listed in Part 1 under Non-UCSF Affiliated Sites

3. Additional description of *general* study design. Attach flow diagram if appropriate. Space limit: half page

RECRUITMENT OF POTENTIAL PARTICIPANTS

- Self-referred individuals responding to flyers posted at UCSF clinics & hospitals
- Colleague-referred patients responding to information sheet about the study
- San Francisco community residents responding to media ads

↓

SCREENING PHONE CALL

↓

SCREENING VISIT

STEP I: Informed Consent Procedures

STEP 2: Major Depression assessed via Mini International Neuropsychiatric Interview
Beck Depression Inventory (BDI-II)
Other Inclusion/ Exclusion Criteria Evaluated

STEP 3: Eligible participants undergo stratified block randomization
to yoga exercise group vs. classroom control group

↓ ↓

YOGA EXERCISE GROUP: **20** PARTICIPANTS
(10 Adults and 10 Adolescents)

- Undergo BDI-II, GSES, RSES at Baseline Visit
- Attend 2 group practices per week for 8 weeks
- Receive reminder calls to maximize adherence
- Undergo BDI-II at 2wks, 4wks, 6 wks
- Undergo BDI-II, GSES, RSES at 8 wks

CLASSROOM CONTROL GROUP: **20** PARTICIPANTS
(10 Adults and 10 Adolescents)

- Undergo BDI-II, GSES, RSES at Baseline Visit
- Attend 2 group classes per week for 8 wks
- Receive reminder calls to maximize adherence
- Undergo BDI-II at 2wks, 4wks, 6 wks
- Undergo BDI-II, GSES, RSES at 8 wks

↓ ↓

COMPLETION OF STUDY ASSIGNMENT

Participants in classroom control group offered yoga exercise sessions at no charge

G. Statistical Analysis: Briefly describe what statistical analysis(es) of which outcome will be applied in order to address each primary aim. Examples of statistical analyses include:

Calculation of descriptive statistics such as mean, median, SD, range, tallies.
Examination of graphs such as outcome vs. time, scatterplots of two variables, Kaplan-Meier curves.
Estimation of differences between two groups with comparison by t-test or Mann-Whitney test.
Estimation and testing of within-person changes by matched t-test or Wilcoxon signed-rank test.
Multiple linear regression, logistic regression, or Cox proportional hazards regression.
Repeated measures models (usually requires the help of a statistician).

For qualitative research, briefly describe how qualitative data will be analyzed.

Overall Analysis Plan

In this study of yoga as an intervention for depression, all statistical analyses will be conducted under the direction of UCSF Senior Statistician Michael Acree, PhD, in close collaboration with the PI. The first step in the analysis will consist of describing in detail the collected data. The data will be screened for the presence of outliers and abnormal values, using graphical analysis and descriptive summaries to ensure that all data fall within expected ranges and to ascertain that the distributions of measures meet the assumptions of the statistical tests to be used.

Primary analysis of all outcomes will be performed using an intention-to-treat approach. Trends in outcome data across multiple assessment points will be modeled for each intervention group. Outcome data will be modeled as correlated within participants, but independent between participants. Covariates including age, gender, ethnicity, socioeconomic status, prior yoga exposure, presence of medical co-morbidities, degree of social support, and depression severity at baseline will be included in the preliminary statistical model as controls, since they may affect both adherence and depression outcomes. Covariates that prove to be non-statistically significant will be omitted in the final model.

A secondary analysis will include as a key predictor the time-varying covariate of percentage of intervention sessions completed by randomized participants between assessment points, and will examine the interaction of this variable with intervention group and with depression outcomes. This will test several ideas: that patterns of adherence will be similar in both intervention groups, that there will be little or no treatment effect in those not adhering to the study protocols, and that adherent participants in the yoga group will show more treatment effect than adherent participants in the control group.

Tests of Primary and Secondary Hypotheses

For Aim 1, which tests the primary hypothesis, the outcome measure will be BDI-II scores. We hypothesize that participants in the yoga exercise group will achieve a statistically significant reduction in BDI-II scores compared to participants in the classroom control group. We will estimate and test a mixed-effects linear regression model of depression symptoms from BDI-II scores obtained at baseline, 2 wks, 4 wks, 6wks, and 8 wks. Unbalanced data will be accommodated by using maximum likelihood estimation, which will allow us to incorporate all collected data in the estimation of the model. In the event that a preliminary analysis indicates non-normal distribution of BDI-II scores, we will pursue techniques for handling non-normally distributed data through generalized estimating equations. The model will include effects for both intervention assignment, which tests whether the mean BDI-II scores differ between the yoga exercise group and the control group, and for intervention-by-time, which tests whether change in mean BDI-II scores differ between measurement intervals.

For Aim 2, which tests a secondary hypothesis, the outcome measure will be scores on the General Self-Efficacy Scale (GSES). We hypothesize that participants in the yoga exercise group will achieve a statistically significant increase in GSES scores compared to participants in the classroom control group. We will estimate and test a mixed-effects linear regression model of GSES scores at baseline and at 8 wks. Unbalanced data will be accommodated by using maximum likelihood estimation, which will allow us to incorporate all collected data in the estimation of the model. Should the preliminary analysis indicate non-normal distribution of GSES scores, we will pursue techniques for handling non-normally distributed data through generalized estimating equations. The model will include effects for both intervention assignment, which tests whether the mean GSES scores differ between the yoga exercise group and the control group, and for intervention-by-time, which tests whether changes in mean GSES scores differ between measurement intervals.

For Aim 3, which also tests a secondary hypothesis, the outcome measure will be scores on the Rosenberg Self-Esteem Scale (RSES). We hypothesize that participants in the yoga exericse group will achieve a statistically significant increase in RSES scores compared to participants in the classroom control group. We will estimate and test a mixed-effects linear

regression model of RSES scores at baseline and at 8 wks. Unbalanced data will be accommodated by using maximum likelihood estimation, which will allow us to incorporate all collected data in the estimation of the model. Should the preliminary analysis indicate non-normal distribution of RSES scores, we will pursue techniques for handling non-normally distributed data through generalized estimating equations. The model will include effects for both intervention assignment, which tests whether the mean RSES scores differ between the yoga exercise group and the control group, and for intervention-by-time, which tests whether changes in mean RSES scores differ between measurement intervals.

Data Analysis for Aim 4

For Aim 4, recruitment response rate, enrollment rate, adherence rate per intervention, attrition rate per intervention, and participant characteristics will be evaluated. The number of participants responding to recruitment ads will be recorded on a daily basis over the course of the entire recruitment period; the sum of all such participants in a given month will be used to derive a monthly recruitment response rate. Similarly, the number of eligible participants who choose to enroll in the study will be recorded on a daily basis over the course of the entire recruitment period; the sum of all such participants in a given month will be used to derive a monthly enrollment rate. Adherence rates in each intervention group will be calculated as the percentage of total assigned sessions completed per protocol by each participant by the last day of the intervention. The number of enrolled participants who drop out of the study and cannot be contacted for the final 8-week assessment will be tallied over the course of the entire recruitment period, and the total number recorded by the last day of the last intervention will be used to derive an overall attrition rate. Evaluating the demographic and other salient characteristics of participants will require extensive use of descriptive statistics, including confidence intervals to describe the data collected. Much of this will derive directly from the preliminary data analysis. Descriptive measures will be constructed for the sample as a whole and also for sub-groupings based on factors such as gender and depression severity.

H. Sample Size: Indicate how many subjects will be studied and why this number was chosen.

As this is a pilot study, the sample size is based primarily on participant and resource availability during the study timeline. One of the main objectives is to provide empirical data for effect size estimation for a future larger randomized trial. A sample size of 40 participants, even with a conservatively estimated attrition rate of 25%, will yield a final sample of 30 participants at the 8-wk assessment point, enabling us to meet the aims of this study using the data analysis methods above.

PART 3: PROCEDURES

A. Check all that apply.

[ ] Human Biological Specimen Banking **Attach - Banking Supplement**
[ ] Genetic Testing [ ] HIV Testing

B. Please list, in sequence, all study procedures, tests, and treatments required for the study. Indicate which would be done even if a participant does not enroll in the study. Include a detailed explanation of any experimental procedures. Attach table if available.

Phone Screen (Done with all respondents to flyers and media ads even if participant does not enroll in study):

Individuals responding to recruitment flyers and ads will undergo a brief telephone screen by study personnel. During this phone screen, respondents will be given a brief description of the study to gauge their interest and ability to participate in the study interventions. Respondents will be asked some basic questions to gauge whether they are likely to meet eligibility criteria; **respondents reporting use of antidepressant medication or psychotherapy at the time of the phone screen will *not* be asked to discontinue these treatments; instead, these respondents will be excluded from further study participation.** Respondents not getting conventional care for depression at the time of the phone screen will be given information about accessing such conventional care as an alternative to study participation. Individuals who appear likely to meet eligibility criteria and who express interest in study participation will be scheduled for a formal screening visit.

Screening Visit, Step 1: Informed Consent Procedure

At the time of the screening visit, the PI will engage participants in a detailed informed consent procedure, using Informed Consent forms approved by the CHR. Participants will be informed that those who complete the screening process and meet all eligibility criteria may choose whether or not to enroll in this study. It will be explained that the main purpose of this study is to determine whether specific yoga practices may improve depression symptoms. Participants will be provided a brief overview of yoga, with explanations of the specific interventions and time commitments related to study participation. Participants will be told that if they choose to enroll in the study, there is a 50% chance that they will get

randomized to the classroom control group instead of the yoga exercise group. However, it will be explained that once the assigned intervention is finished, anyone randomized to the classroom control group will be offered 16 free yoga sessions at UCSF, where they can learn and practice the same yoga exercises assigned to the yoga exercise group.

During the informed consent procedure, it will be explained that antidepressant medications and/or psychotherapy are the usual first-line treatments for major depression. All participants will be offered information to access conventional treatment with antidepressant medications and/or psychotherapy. **Participants reporting use of antidepressant medication or psychotherapy at the time of the informed consent procedure will *not* be asked to discontinue these treatments; instead, these individuals will be excluded from the study.** Participants will be informed that if they choose to enroll in the study, they are opting for an 8-week controlled trial of yoga for depression, instead of obtaining conventional treatment for depression. It will be explained that enrolled participants may not use any antidepressant medication or psychotherapy during the study period. However, participants will be informed that any individuals enrolled in the study may withdraw from the study at any time should they change their minds and opt for conventional care.

Participants will be informed of the risk that their depressive symptoms may worsen over the 8-week course of the study. It will be explained that everyone enrolled in the study will be monitored very closely, and if there are signs of worsening depression, an enrolled participant will be withdrawn from the study and referred for conventional psychiatric care. Participants will also be informed of potential physical risks related to practicing yoga exercises, including risks of mild musculoskeletal discomfort or more serious musculoskeletal injuries. They will be informed that the yoga instructor for the study is a licensed, registered nurse who will take care to teach the poses in such a way that these physical risks are minimized; however, should any injury occur during the yoga practice, the nurse-instructor will be available to provide immediate medical assistance as necessary.

Girls and women of child-bearing age will be told that to protect them from very rare but potentially serious physical risks related to mild hyperventilation in the practice of some yoga breathing exercises, they will be tested for pregnancy and excluded from the study if they are pregnant. Girls and women of child-bearing age who are not pregnant will be advised to use birth control or avoid pregnancy before and during the study.

Those individuals who choose to sign the Informed Consent forms will receive a unique, randomly-generated identification number, and will proceed to Step 2 of the screening. The identification number will be used throughout the entire study on all study documentation related to that participant.

<u>Screening Visit, Step 2: Physician Evaluation</u>
Individuals signing the Informed Consent forms will next undergo a medical and psychiatric evaluation by the screening physician, who is the PI. The physician will meet individually with each participant to ascertain study eligibility using the following procedures:

- Diagnosis of major depression via the validated, structured Mini International Neuropsychiatric Interview (MINI).
- Rating of depression severity via the validated Beck Depression Inventory (BDI-II).
- Assessment of cognitive status via the validated Folstein Mini-Mental Status Exam (MMSE).
- Assessment of co-morbid medical and psychiatric conditions via detailed medical and psychiatric history.
- Focused physical exam to ascertain sufficient physical fitness to perform all yoga exercises.
- Pregnancy status of all girls and women of child-bearing age, using a urine pregnancy test.

Participants reporting use of antidepressant medication or psychotherapy at the time of the physician evaluation will *not* be asked to discontinue these treatments; instead, these individuals will be excluded from the study. Everyone completing the physician evaluation will receive a voucher to attend one free meditation class at UCSF, regardless of whether he or she meets the eligibility criteria. Consenting individuals meeting all eligibility criteria will proceed with Step 3 of the screening visit.

<u>Screening Visit, Step 3: Randomization to Study Intervention</u>
During this third and final step of the screening process, all participants meeting eligibility criteria will be assigned to a specific study intervention by the statistician, who will use a block randomization method to ensure that each intervention group has equal numbers of participants with mild depression (per BDI-II scores of 14 to 19) and those with moderate depression (per BDI-II scores of 20 to 28).

Study assignment will occur as follows: upon completion of the physician evaluation, a study assistant will contact the statistician for an intervention assignment for each eligible participant. The statistician will be faxed a summary

eligibility page from the physician screening records, indicating whether a given participant (referenced only by the Study ID number) has either mild depression or moderate depression. Reviewing these data and using a pre-determined, computer-generated SAS algorithm for block randomization to ensure that each intervention group has equal numbers of participants with mild versus moderate depression, the statistician will fax back to the study assistant a specific study assignment for the participant; the study assignment will simply be labeled "Group A" or "Group B." Except for the statistician, no study personnel will have access to the randomization algorithm before all interventions are completed. The study assistant will communicate the intervention assignment of either Group A or Group B to the participant, indicating when and where that group will be meeting. Through this process, allocation of study assignment will occur independently of the screening physician, and remain concealed from the screening physician.

Baseline Visit

Just prior to the first session of the assigned intervention, each participant will be asked to complete a baseline Beck Depression Inventory (BDI-II), baseline Rosenberg Self-Esteem Scale, and baseline Generalized Self-Efficacy Scale.

Study Intervention (Classroom Control Group versus Yoga Exercise Group)

Within one week of completing all screening procedures, enrolled participants will begin attending their assigned intervention group 2 times per week for 8 weeks total. Intervention sessions for both the classroom control group and the yoga exercise group will be available twice-weekly on a rolling basis throughout the recruitment period until the last enrolled participant has completed the study protocol. The instructor will take attendance at each session, identifying participants only by their unique study identification numbers.

The control intervention will consist of 90-minute sessions of engaging classroom instruction covering the history of yoga, the main branches of yoga currently practiced in India and the United States, and the corresponding yoga philosophy associated with each branch. Documentary film clips will be used to enhance the classroom lectures and engage participants. The classroom instruction is designed to control for potential non-specific treatment effects (such as instructor attention, peer interaction, and shift from routine activities) associated with the active yoga intervention. Participants in the classroom control group will be asked to refrain from practicing yoga exercises of any kind during the 8 wks of the study.

The active yoga intervention will consist of 90-minute sessions of specific, gentle yoga stretches and postures combined with specific yoga breathing exercises. The yoga instructor for the study is a licensed, registered nurse who will teach participants to remain within their usual range of motion or comfort in performing the yoga exercises. The nurse-instructor will make appropriate accommodations for any participants with limitations in range of motion or limited tolerance for yoga exercises. Should an injury occur during the practice of yoga exercises, the nurse-instructor has the capacity to provide immediate medical evaluation and assistance as necessary. Participants in the yoga exercise group will be asked to refrain from practicing any yoga exercises outside of the assigned study sessions during the 8 weeks of the study.

The yoga postures and breathing exercises used in this study are described in more detail below, and are adapted from the yoga practices for depression compiled by the Swami Vivekananda Yoga Research Foundation.[65]

YOGA POSTURES:

- The first yoga posture is called Ardhakati Chakrasana (Half Waist Wheel Pose). Participants stand upright and then gently bend from the waist sideways to the right as far as comfortable, while stretching the left arm upward. Returning to an upright stance, they repeat the exercise, but bending to the left while stretching the right arm up.
- The second yoga posture is called Ardha Chakrasana (Half Wheel Pose). Participants stand upright with their hands supporting the lower back. They gently bend backwards from the lower lumbar region as far as comfortable, allowing the neck to drop slowly while their hands continue to support the lower back.
- The third yoga posture is called Pada Hastasana (Hands to Feet Pose). Participants will stand upright with arms raised, and then gently bend forward until the trunk is parallel to the ground; those participants flexible enough will then continue to bend forward until the hands are touching the feet and the head is touching the knees.
- The fourth yoga posture is called Bhujungasana (Cobra Pose). Participants will lie on the ground in a prone position. They will gently arch the head and spine backwards as far as comfortable, supporting the torso with arms bent at the elbow and palms firmly on the ground.
- The fifth yoga posture is called Dhanurasana (Bow Pose). Participants will lie on the ground in a prone position. Knees will be bent toward the buttocks as far as possible and the neck will gently arch backward. Participants flexible enough will gently grasp the ankles with their hands.
- The sixth yoga posture is called Sarvangasana (Shoulder Stand). Participants will lie on the ground in a supine

position. Participants with sufficient strength and flexibility will raise the legs slowly until the legs are perpendicular to the ground; other participants will position their legs against a wall so that the legs are as perpendicular to the ground as possible. Those with sufficient strength and flexibility will then raise the buttocks and trunk off the ground, keeping the shoulders and elbows firmly on the ground and supporting the back with both palms while the legs remain perpendicular to the ground.

- The seventh yoga posture is called Matsyasana (Fish Pose). Participants will lie on the ground in a supine position. Hands are placed palm down underneath the back, with the fingers pointing toward the spine. Pressing down on the palms, participants will slowly raise the chest and arch the back as far as comfortable, with the weight of the torso supported on the elbows.
- The eighth yoga posture is called Setu Bandhasana (Bridge Pose). Participants will lie on the ground in a supine position. Then the knees are bent while keeping the soles of the feet on the ground. Arms are kept alongside the torso, but with palms on the floor. The hips are lifted off the floor towards the ceiling as far as comfortable, keeping the feet and palms flat on the floor.
- The ninth yoga posture is called Balasana (Child Pose). Participants kneel on the floor with feet tucked under the buttocks if possible. Participants then bend forward at the waist, bringing the chest as close to the knees as possible, and resting the forehead on the floor, if flexible enough. Arms are extended on the ground, palms down.
- The tenth yoga posture is called Vakrasana (Twisted Pose). Participants sit on the floor with both legs outstretched. Then the left leg is bent at the knee with sole of the left foot placed on the ground alongside the right knee. The upper part of the body is twisted to the left as much as possible, keeping the shoulders in plane with the chest and supporting the torso on the left arm, with left hand firmly planted palm-down on the ground. The elbow of the right arm should be straight and should be pressed against the lateral surface of the bent left knee. After holding this pose, participants return the torso, arms and legs to the beginning position. Then participants repeat this pose so that the right leg is bent and the upper body twists to the right side.
- The eleventh yoga posture is called Ustrasana (Camel Pose). Participants kneel on the floor, keeping the neck and spine perpendicular to the ground. Then the spine is arched back gently so that the chest puffs out. Participants reach the hands back one at a time to grasp the heels if they are flexible enough. Others can reach back to yoga blocks placed on either side of the feet.
- The twelfth yoga posture is called Shavasana (Corpse Pose). Participants lie on the floor in a supine position, with eyes closed. Arms are stretched away from the torso, palms up. Legs are separted about a shoulder width apart, and the feet rotate out slightly.

YOGA BREATHING EXERCISES:

- The first set of breathing exercises is called Nadi Shodana (Alternate Nostril Breathing). Participants will receive instruction in a relaxed yoga breathing pattern where inhalation and exhalation occurs only through one nostril at a time. Breathing will take place at a normal rate of about 12 cycles/minute.
- The second set of breathing exercises is called Ujjayi (Victorious Breath), and this consists of deep, slow breathing completed at the rate of 4 cycles/minute against mild airway resistance. Participants will be instructed on techniques to slightly contract laryngeal muscles and partially close the glottis in order to maintain mild airway resistance throughout inspiration and expiration.
- The third set of breathing exercises is called Bhastrika (Bellows Breath), a technique in which the breath is forcefully inhaled and then exhaled through nostrils at a rate of about 20-30 cycles/minute while using strong abdominal muscle contractions.
- The fourth set of breathing exercises is called Brahmari (Bumblebee Breath). In this slow breathing technique, participants will close their eyes and ears as they inhale slowly and then make a gentle humming sound with each exhalation. Breathing will occur at a rate of about 6 cycles/minute.

<u>Week 2 Visit</u>
After finishing the second week of the assigned intervention sessions, each participant will be asked to complete a Beck Depression Inventory (BDI-II) to assess depression symptoms.

<u>Week 4 Visit</u>
After finishing the fourth week of the assigned intervention sessions, each participant will be asked to complete a Beck Depression Inventory (BDI-II) to assess depression symptoms.

<u>Week 6 Visit</u>

After finishing the sixth week of the assigned intervention sessions, each participant will be asked to complete a Beck Depression Inventory (BDI-II) to assess depression symptoms.

Week 8 Visit

After finishing the last intervention session on the eighth week, participants will be asked to complete a Beck Depression Inventory (BDI-II) to assess depression symptoms. They will also complete the General Self-Efficacy Scale, the Rosenberg Self-Esteem Scale, and a feedback questionnaire related to their general experience with the study protocol. Even if participants do not choose to complete all 8 weeks of the intervention, they will be contacted and requested to complete the Week 8 outcome measures and final feedback questionnaire.

SUMMARY CHART OF STUDY PROCEDURES

	**Phone Screen**<br><br>(All respondent to flyers and media ads)	**Screening Visit**<br><br>(Respondents appearing eligible after phone screen)	**Baseline Visit**<br><br>(Only enrolled participants)	**Week 2 Visit**<br><br>(Only enrolled participants)	**Week 4 Visit**<br><br>(Only enrolled participants)	**Week 6 Visit**<br><br>Only enrolled participants)	**Week 8 Visit**<br><br>(Only enrolled participants)
Telephone Screening Form	X						
Consent Forms		X					
MINI		X					
BDI-II		X	X	X	X	X	X
Folstein MMSE		X					
Physican Evaluation Form		X					
Self-Esteem Scale			X				X
Self-Efficacy Scale			X				X
Study Intervention			2 sessions per week for 8 weeks ;<br>Sessions begin after Baseline Visit & end at Week 8 Visit				
Final Feedback Questionnaire							X

C. Time Commitment: Indicate how much time will be required of the subjects, per visit and in total for the study.

Phone Screen: 10 minutes
Screening Visit: 2 hours
Baseline Visit: 15 minutes
Study Intervention: 24 hours (16 total sessions with 90 minutes per session)
Week 2 Visit: 5 minutes
Week 4 Visit: 5 minutes
Week 6 Visit 5 minutes
Week 8 Visit: 30 minutes

TOTAL FOR STUDY: 27 hours and 10 minutes

D. Facilities: List the clinics and/or other specific locations where study procedures will be performed. Please provide a description of the facility if appropriate. For example, if study procedures involving more than minimal risk take place in a research facility, a description of the equipment on hand needed to protect participants would be appropriate. Attach letters of support indicating knowledge and endorsement of this study from any involved units or name investigators from those units under Key Personnel.

All study procedures will be performed at the UCSF Osher Center for Integrative Medicine, where the PI of this proposed study is a core faculty member with full access to the facilities required for this project.

The Osher Center for Integrative Medicine at UCSF is a campus-wide multidisciplinary program established in 1997 with

$2.2 million in seed money from the School of Medicine and UCSF Medical Center, and a $10 million endowment from the Bernard Osher Foundation. Operating within the School of Medicine, the Osher Center has developed strong ties to the community, public health agencies, and professional organizations related to integrative medicine. The missions of the Osher Center are: 1) to scientifically research the effectiveness and value of integrative medicine; 2) to bring to the patient integrative care of proven value; 3) to bring to students and practitioners in the health professions a new paradigm of relationship-centered medicine; and 4) to disseminate information on the efficacy of integrative medicine to the public and the medical community.

Clinical Facilities

The Clinical Programs at the Osher Center for Integrative Medicine are located at 1701 Divisadero Street, San Francisco, CA, and opened in the late spring of 2002. The specially designed clinic areas occupy approximately 2800 sq. ft. and include an interactive waiting area, a classroom equipped with audio-visual presentation equipment, five exam rooms, a confidential interview room, and a large 720 sq. ft. studio for yoga and other group programs. The exam rooms are located on the 1st floor, and each occupies approximately 200 square feet. The yoga studio has the benefit of natural light as well as adjustable lighting and temperature control; built-in shelving is stocked with supplies for yoga and meditation classes (mats, blankets, bolsters, belts, and cushions of various shapes to meet the needs of people with special health requirements.) The clinic serves approximately 600 patients annually, offering a variety of services including acupuncture, psychotherapy, mindfulness based stress reduction, support groups and yoga.

Office and Computer Resources:

The Osher Center for Integrative Medicine includes fully equipped private office space for research staff. It has an off-site Windows 2000 file and print server that is located in a secure environment with a back-up power supply. All files are backed up daily and each week; full-system backup tapes are removed to an off-site location to ensure the safety of the data. Individual desktop computers operate under Windows 2000 Professional and are password protected. This fully supported network system includes virus protection software on all servers and workstations that is updated regularly. The building is connected to the rest of the university via a high-speed Internet connection using T-1 data line for email, Internet browsing and file transfers.

Emergency Resources:

If necessary, exam rooms and medical equipment for assessing urgent health concerns are available on-site at the Osher Center for Integrative Medicine. All campus sites on UCSF, including the Mt Zion Campus where the Osher Center for Integrative Medicine is located, have centralized mechanical and electrical support for emergencies or special needs. Custodial support, regulatory oversight, emergency power, and occupational safety guidelines are all in place and adherence to these guidelines is met. The UCSF Mt Zion campus is connected to the main UCSF Medical Center and Emergency Dept on Parnassus by a free shuttle bus departing every 15 minutes from 6:45 am to 6:30 pm Monday- Friday.

**D.** Will any interviews, questionnaires, surveys or focus groups be conducted for the study? If "Yes," please list any standard instruments used for this study and attach any non-standard instruments.	[X]Yes [ ]No

This study will use the following standard instruments:

1) Mini International Neuropsychiatric Interview (MINI)

During the screening visit (Step 2), the Mini International Neuropsychiatric Interview will be used to diagnose major depression and exclude co-morbid psychiatric disorders in potential participants. The MINI is a short structured diagnostic interview developed jointly by psychiatrists and mental health clinicians in the U.S. and Europe. Diagnostic questions on the MINI parallel symptoms listed in the American Psychiatric Association's Diagnostic and Statistical Manual of Mental Disorders, 4th Edition. The MINI can be administered in approximately 15 minutes by trained clinicians, meeting the need for a short but accurate diagnostic instrument for clinical trials in psychiatry. It has acceptably high reliability in diagnosis compared with highly validated but more time-consuming instruments developed by the World Health Organization.

2) Beck Depression Inventory (BDI-II)

Depression severity will be assessed with the Beck Depression Inventory-II BDI-II, administered to study participants at the Screening Visit (Step 2), and again at the Baseline Visit, at the 2 week Visit, at the 4 week Visit and at the 8 week Visit. The BDI-II is one of the most commonly used instruments in psychiatric research; it has been translated and validated in many different languages, appearing in hundreds of studies worldwide. The BDI-II is a 21-item validated

instrument for the self-report of depressive symptoms; it can typically be completed in 5 minutes. Each item on the BDI-II can be scored from 0 to 3, with the total score derived by summing the individual item scores. A total score of 14-19 suggests mild depression, 20-28 suggests moderate depression, and 29-63 suggests severe depression.

3) Folstein Mini Mental Status Exam (MMSE)
During the Screening visit (Step 2), basic cognitive capacity of potential participants will be measured with the Folstein Mini Mental State Examination (143, 144). The MMSE is a widely used instrument for assessing cognition in both clinical and research settings. It assesses orientation, attention, immediate and short-term recall, language, and the ability to follow simple verbal and written commands, providing a total score than can range from 0-30. Cognitive performance as measured by the MMSE varies within the population by age and educational level. In general, scores below 23 may be suggestive of cognitive impairment.

4) General Self-Efficacy Scale
Participants will be administered the General Self-Efficacy Scale (GES) at the Baseline Visit and again at the Week 8 Visit. The GSES is a 10-item psychometric scale that is designed to assess self-efficacy, i.e., the belief that one's actions are responsible for successful outcomes in coping with a variety of difficult life demands. The scale was originally developed in 1979, and has been validated since then in many international studies. The scale was designed for individuals age 12 years and up. The scaled score for each question ranges from 1 to 4, with the total score derived by summing the individual question scores. Higher total scores indicate stronger belief in one's self-efficacy.

5) Rosenberg Self-Esteem Scale
Participants will be administered the Rosenberg Self-Esteem Scale (RSES) at the Baseline Visit and again at the Week 8 Visit. The RSES is a 10-item scale developed in 1965, and is a widely-used measure of self-esteem in social science and psychiatric research with adolescents and adults. It is designed to assess overall feelings of self-worth and self-acceptance. Validated studies have demonstrated both a uni-dimensional and a two-factor (self-confidence and self-deprecation) structure to the scale. The scaled score for each question ranges from 0 to 3, with the total score derived by summing the individual question scores. Higher total scores indicate self-esteem.

This study will also use the following non-standard questionnaires and forms:

6) Telephone Screening Form (see attached Appendix B)
7) Physician Evaluation Form (see attached Appendix C)
8) Final Feedback Questionnaire (see attached Appendix D)

**E.** Will subjects undergo any study procedures or tests off-site by non-UCSF personnel? If "Yes," please explain.	[ ]Yes [X]No

**F.** Will subjects or their health care provider be given the results of any experimental tests that are performed for the study? If "Yes," please describe the tests, provide a rationale for providing subjects with the experimental test results and explain what, how and by whom subjects and their health care provider will be told about the meaning, reliability, and applicability of the test results for health care decisions.	[ ]Yes [X]No

PART 4: ALTERNATIVES

A. Describe the standard or usual care or activities at UCSF (or study site) that are available to prospective subjects who do not enroll in this study.

Prospective participants who do not enroll in this study may obtain conventional mental health treatments at UCSF to address their depression symptoms. Conventional mental health treatments available at UCSF include outpatient pharmacotherapy and/or psychotherapy in primary care clinics, as well as outpatient care at Langley Porter Psychiatric Institute using anti-depressant medications, individual psychotherapy, and/or group psychotherapy. Other, more intensive

conventional mental health treatment available at UCSF includes Partial Psychiatric Hospitalization as well as inpatient psychiatric hospitalization at Langley Porter. During the screening process, all prospective participants will be offered information to access these conventional treatment options at UCSF.

B. Describe other alternatives to study participation that are available to prospective subjects.

Individuals who choose not to participate in this study may alternatively seek yoga classes at community health clubs or yoga studios, and if they wish, combine these yoga classes with conventional mental health care.

**C.** Is study drug or treatment available off-study? If "Yes," discuss this in the consent form.	[X]Yes [ ]No [ ]N/A Community yoga classes

PART 5: RISKS AND BENEFITS

A. Risks and Discomforts:

1. Describe the risks and discomforts of any investigational or approved drugs, devices and procedures being used or assigned for study purposes. Describe the expected frequency of particular side effects. If subjects are restricted from receiving standard therapies during the study, please also describe the risks of those restrictions.

Potential Psychological Risks of Study Participation
Individuals with mild to moderate depression choosing to enroll in this study are opting for participation in an 8-week controlled trial of yoga for depression rather than obtaining conventional treatment with antidepressant medication or psychotherapy. Individuals with mild to moderate depression enrolled in this study face some risk that their depression symptoms may increase in severity over the 8-week course of study participation. Worsening of depressive symptoms may have adverse social and vocational consequences for the participant; in a very small number of severe cases, worsening of depressive symptoms may increase suicide risk.

It is difficult to quantify the potential risk of suicide that may be associated with study participation. However, we anticipate that individuals choosing to enroll in this clinical trial of yoga for depression are likely to have some positive expectations of mood benefit, similar to placebo drug recipients enrolled in an antidepressant medication trial. Thus, in considering suicide risk for depressed participants in our proposed study, it may be useful to review data on the incidence of suicide among the many thousands of participants randomized to the placebo arm of placebo-controlled antidepressant medication trials over the past few decades:

- Several large meta-analyses[66-68] of placebo-controlled antidepressant medication trials have demonstrated that the overall incidence of suicide among depressed adults is quite small (ranging from 0.02% to 0.20% in these pooled meta-analyses), and *the incidence of suicide is statistically comparable between the cohort treated with the placebo drug and the cohort treated with the active antidepressant medication*. In one very large, recent meta-analysis,[69] young adults 18-24 years of age who were assigned to placebo actually had a slightly *decreased* incidence of suicidal ideation or behavior in comparison to age peers assigned to active antidepressant medicine.

- Similarly, in a large meta-analysis[23] of pediatric placebo-controlled antidepressant medication trials, children under 18 years of age assigned to placebo demonstrated a *decreased* incidence of suicidal ideation or behavior in comparison to age peers assigned to the active antidepressant medication.

Based on the above data, we anticipate that the potential risk of suicide associated with participation in this study is likely to be quite low, and comparable to the low overall incidence of suicide previously observed among depressed participants randomized to the placebo arm of placebo-controlled antidepressant medication trials.

Potential Physical Risks of Study Participation
We anticipate only minimal physical risks associated with the yoga exercises in this study, as all our yoga exercises involve low-impact, highly controlled motions restricted to each individual's own range of motion, and will be taught and supervised by a skilled nurse-instructor. In this context, we anticipate that minor, transient muscle discomfort will be the most likely physical risk of study participation. No adverse physical events occurred in the preliminary open trial[64] of

yoga previously conducted by the Dr. Prathikanti at UCSF. Similarly, no adverse physical events were reported in any of the yoga studies[50-54] reviewed above in Section D of this CHR application.

There are very small *theoretical* risks that some yoga breathing exercises involving a mildly increased respiratory rate may exacerbate certain pre-existing medical conditions. There is the risk that increased respiratory rate may contribute to cerebral hypo-perfusion in people with carotid artery stenosis or uncontrolled severe hypertension, to lowering of seizure threshold in people with epilepsy, or to exacerbation of breathing difficulties in individuals with chronic obstructive pulmonary diseases (COPD). To protect against these possible physical risks, individuals identified in the screening process as having any of the above pre-existing medical conditions will be excluded from the study.

<u>Potential Reproductive Risks of Study Participation</u>:
There is a very small *theoretical* risk that some yoga breathing exercises involving a mildly increased respiratory rate may exacerbate a resting hyperventilation syndrome observed in some pregnant women due to pregnancy-related physiological changes. Exacerbation of resting hyperventilation may increase risk of cerebral hypo-perfusion in pregnant women and their developing babies. To protect against this risk, pregnant women will be excluded from this study.

2. Describe the steps you have taken to minimize the risks/discomforts to subjects. Examples include: designing the study to make use of procedures involving less risk when appropriate; minimizing study procedures by taking advantage of clinical procedures conducted on the study participants; mitigating risks by planning special monitoring or conducting supportive interventions for the study. If appropriate, provide a rationale for risky procedures.

<u>Minimizing Psychological Risks</u>
The PI of this project has implemented a variety of steps to decrease psychological risk to participants:

1) Several distinct opportunities during the screening process have been created for potential participants to learn about conventional treatment options for depression, and to receive referral for conventional care rather than to enroll in the study. These distinct opportunities will occur during the telephone screen, during the informed consent procedure, and during the physician screening evaluation. At each of these junctures, participants will be informed that antidepressant medications and/or psychotherapy are standard treatments for depression, and all participants will be offered information to access these conventional treatments. Bearing in mind that only one-third to one-half of Americans with depression typically obtain referrals for conventional mental health treatment,[70-72] our screening process may offer many depressed respondents more opportunities for referral to conventional care than they might otherwise obtain. Participants reporting use of antidepressant medication or psychotherapy at the time of screening will *not* be asked to discontinue these treatments; instead, to minimize risk, these individuals will be excluded from study participation.

2) No direct monetary payment will be provided to study participants. Therefore, depressed individuals will have little financial incentive to avoid or discontinue conventional mental health therapies in order to take part in this study.

3) This study is designed to enroll only individuals with mild to moderate depression, and will explicitly exclude those with suicidal ideation, psychotic symptoms, or depression symptoms considered to be of severe intensity as measured by scores above 28 on the screening Beck Depression Inventory. Recent rigorous meta-analyses[73,74] of placebo-controlled anti-depressant medication trials compared mood benefits of active medication versus placebo in hundreds of depressed adult participants; authors of these meta-analyses concluded that while the benefit of active medication over placebo may be significant for people with severe depression, any such benefit appears to be minimal or nonexistent for people with mild or moderate depression. Thus, by limiting our proposed study sample to people with mild to moderate depression, we are recruiting only those depressed individuals for whom a conventional treatment is of uncertain benefit.

4) Instead of randomizing some participants to a waitlist control group with little to no intervention, the design of this clinical trial offers participants in *both* the control group and the active yoga group significant amounts of staff attention, peer interaction, and cognitive engagement. These factors are likely to have positive mood effects in both groups, helping to mitigate risks of clinical decline. This design also provides many opportunities for study personnel to monitor participants in *both* groups for any evidence of clinical decline.

5) Following enrollment, all participants will be closely monitored by study personnel for any intensification of depressive symptoms over the study period. Clinical monitoring will occur as follows:

- All participants will be given contact information to reach the study psychiatrist (the PI) by telephone 24 hours a

day and 7 days a week should they feel that their mental health is deteriorating in any way during the study period. Any participant telephoning the study psychiatrist will be assessed for immediate withdrawal from the study and for referral to conventional psychiatric care as appropriate.

- For both the control group and the active yoga practice group, the instructor will be an experienced registered nurse with training in the clinical monitoring of major depression. During each intervention session, the nurse-instructor will invite any participants who feel their depression is worsening to meet with her individually. During these individual meetings with the nurse-instructor, participants specifically will be asked about suicidal ideation, psychosis, difficulty with self-care, or other signs of intensifying depression. Participants endorsing such symptoms of clinical decline will be directed to immediate evaluation by the study psychiatrist, who can withdraw the participant from the study and coordinate appropriate referral to conventional psychiatric care.
- Every two weeks, a formal Beck Depression Inventory (BDI-II) will be administered to each participant to follow the severity of depression symptoms. A blinded assessor will compare the participant's baseline BDI-II score with the BDI-II score at each 2-week assessment interval. Should the participant's score at any 2-week assessment interval show an increase of more than 3 points from his or her baseline score, or should the participant endorse suicidal ideation or psychotic symptoms on the BDI-II, this will automatically trigger a face-to-face evaluation with the study psychiatrist, who can withdraw the participant from the study and coordinate appropriate referral to conventional psychiatric care.
- Throughout the study period, participants will receive phone calls twice a week, reminding them to attend each upcoming intervention session. Any participant who fails to attend a scheduled session will be telephoned by the nurse-instructor within 24 hours to ascertain reasons for absence; the nurse-instructor will direct any participant who discloses suicidal ideation, psychosis or subjective worsening of depression to an immediate evaluation by the study psychiatrist for withdrawal from the study and for referral to conventional psychiatric care. If a participant misses a scheduled study session and cannot be reached within 2 working days to ascertain the reason for absence, a home visit will be made to check on the participant's safety and to take further action as needed.
- Prior to contact with any study participants, all study personnel (including study assistants and the nurse-instructor who teaches the yoga classes) will undergo an intensive two-day training workshop led by the PI to learn about the signs and symptoms of major depression and to recognize possible clinical decline in participants.

Minimizing Physical Risks

We anticipate that minor, transient muscle discomfort will be the most likely physical risk of study participation. To minimize this risk and to protect against more serious injury during yoga classes, all the yoga exercises selected for this study are low-impact and involve highly controlled movements. All yoga exercises will be taught and supervised by a skilled nurse-instructor. The nurse-instructor will take great care to emphasize to participants that they should not go beyond their usual range of motion/comfort for any of the yoga exercises. All yoga sessions will be held at the UCSF Osher Center for Integrative Medicine, where exam rooms, medical equipment, and physician back-up will be readily available to the nurse-instructor should any unexpected medical problems arise in the course of these sessions.

During the physician screening evaluation, individuals vulnerable to more serious physical risks due to pre-existing medical conditions will be excluded from the study. Individuals with significant musculoskeletal problems will be excluded from the study to protect against physical distress or injury from participation in the yoga exercises. Individuals with history of seizure disorder will be excluded from the study to protect against the risk that some yoga breathing exercises may contribute to a lower seizure threshold. Those with carotid artery stenosis or uncontrolled hypertension will be excluded from the study to protect against the risk that some yoga poses or breathing exercises may contribute to transient cerebral hypo-perfusion. Individuals with severe pulmonary problems will be excluded from the study to protect against the risks of exacerbating respiratory distress during any of the breathing exercises.

Minimizing Potential Reproductive Risks

During the physician screening evaluation, pregnant girls and women will be excluded from study participation via a urine pregnancy test. This is to minimize the very small but serious theoretical risk that some of the yoga breathing exercises in this study may exacerbate a mild resting hyperventilation syndrome sometimes accompanying pregnancy. Exacerbation of resting hyperventilation may increase risk of cerebral hypo-perfusion in pregnant women and their developing babies. To protect against this risk, pregnant girls and women will be excluded from this study. Girls and women of child-bearing age who are not pregnant will be advised to use birth control or avoid pregnancy before and during the study.

B. Data and Safety Monitoring Plan (DSMP): ***All interventional studies involving more than minimal risk must include a DSMP.*** A DSMP is a plan established to assure that each research study has a system for appropriate oversight and monitoring of the conduct of the study to ensure the safety of participants and the validity and integrity of the data. The DSMP should indicate specifically whether or not there will be a formal Data Safety Monitoring Board (DSMB) or Data Monitoring Committee (DMC).
Note: Most, but not all studies (i.e., non-interventional studies) undergoing full committee review will require a DSMP.
For *what to include in a DSMP* see DSMP Information Sheet for Principal Investigators.

Overview of Data and Safety Monitoring Plan
The current research project is an interventional study involving greater-than-minimal risk, but is not an NIH-defined phase III clinical trial. Therefore, neither a Data and Safety Monitoring Board (DSMB) nor Data Monitoring Committee (DMC) is required. Rather, a Data and Safety Monitoring plan (DSMP) will be put into effect, as described below, to ensure the safety of participants and the validity and integrity of the dataset. Given the relatively small size and scope of this single-center research project, continuous, close monitoring by Dr. Prathikanti, the Principal Investigator, comprises an adequate and appropriate format for data and safety monitoring, with prompt reporting of any adverse events to the UCSF CHR and to study sponsors.

All participants will be encouraged to contact the Principal Investigator and/or the UCSF CHR to report complaints or adverse events. Instructions for reporting adverse events and complaints, as well as for contacting the PI, are included in the consent documents and on emergency contact cards provided to participants at the time of enrollment. If a participant with a complaint or concern does not contact the PI, but instead contacts another member of the research team (i.e., yoga instructor or research assistant), the participant will be immediately directed to the PI. The PI is an experienced faculty physician in the UCSF Department of Psychiatry, working with high-risk, medically ill psychiatric populations over the past 15 years; the PI is competent to evaluate any adverse psychiatric or medical events that may arise in the course of this research. The PI will assess whether any serious adverse event or serious problem in the conduct of the study has occurred, and if so, take immediate and appropriate action to address the situation. The PI will also report any adverse event/incident in writing within 10 working days to the UCSF CHR and to the study sponsors. Additionally, all adverse events will be reported to study sponsors and the UCSF CHR on the annual progress report.

Study Performance Review
The Principal Investigator will generate monthly administrative reports that describe the progress of the study, including the following information:

- Total accrual for the study
- Participant status in study (e.g. screened, enrolled, dropped out)
- Demographics of participants as a group
- Errors regarding adherence to the eligibility criteria or the study protocol
- Number and type of any serious adverse events

These administrative reports will be reviewed internally by the statistician and by the principal investigator for ongoing quality control. The reports will summarize whether accrual projections are being met, whether any serious adverse events have occurred, and whether the trial should be continued. The PI will use data from these reports to generate annual progress reports submitted to the CHR and to study sponsors.

Safety Reports
If any serious adverse event occurs or if any problems arise in the conduct of the study or with an individual's participation in the study, the PI will report this in writing to the UCSF CHR within 10 working days. The PI's report will provide a relevant discussion of the adverse event or the specific problems related to the conduct of the study or an individual's participation in the study. All information and discussion related to the Adverse Event Report/Incident Report will be kept confidential, and the participant will be referenced only by his or her Study Identification Number.

Additionally, the PI will provide an annual safety report to the CHR and to study sponsors. The annual report will discuss and summarize all adverse events or study problems noticed over the course of the year, including:

- the most frequent and most serious adverse events

- a summary of all Adverse Event Reports/Incident Reports submitted during the past year
- a list of any participants who may have died during the study, with the cause of death for each participant
- a list of participants who dropped out of the study in association with an adverse event, whether or not the adverse event was thought to be study-related

Steps Emanating from Data Review
In reviewing safety and accrual data from the monthly study performance reports, the PI and study statistician will consider potential amendments to the study protocol, any changes to the data collection plan or study forms, and risks/benefits of continuing the trial. If an amendment to the study protocol is considered, the CHR will be notified, and CHR approval will be obtained prior to implementing the amendment. However, in rare cases, the amendment may be implemented at once to protect the safety of the study participants; in such rare cases, the UCSF CHR will be notified immediately after the protocol amendment has been implemented. If a change is considered to the data collection plan or study forms, the UCSF CHR will be notified, and CHR approval obtained prior to implementing any such changes.

C. Adequacy of Resources:
Principal Investigators must have the necessary resources required to conduct the proposed research in a way that assures the rights and welfare of participants are adequately protected. Depending on the nature of the study, investigators should consider the proximity or availability of critical resources that may be essential to the safety and welfare of participants. For example, the proximity of an emergency facility for care of participant injury, or availability of psychological support after participation, or resources for participant communication, such as language translation services.

Have you or will you undergo formal resource review (e.g., VAMC, SFGH, CCRC) prior to study implementation?	[X] Yes [ ] No

If yes, please specify entity providing review:
In late March 2010, this project will undergo formal resource review by Rick Hecht, MD, Director of Research at the UCSF Osher Center for Integrative Medicine. However, in Part 3, Section D, please note the specific resources for this proposed project that are available to the PI as a core faculty member at the UCSF Osher Center. These resources will help ensure that this research project can be conducted in a way that protects the rights and welfare of study participants.

If no, please describe below the resources you have in place to conduct this study in a way that assures protection of the rights and welfare of participants:

D. Confidentiality and Privacy: Privacy concerns people, whereas confidentiality concerns data. Specifically, confidentiality refers to the researcher's plan to handle, manage and disseminate the participant's identifiable private information. Privacy refers to a person's wish to control the access of others to themselves. **Address each of the following privacy issues questions 1-3 below:**

1. How will the investigator access information from or about participants?

All information will be gathered directly from participants in the form of self-report during the screening process.

2. How will the investigator maintain privacy in the research setting(s)?

The principal investigator and key personnel have completed education on HIPAA compliance and on the protection of human subjects in research. They will take every measure to protect participants' privacy and confidentiality. Only relevant research personnel, authorized by the PI, will have access to any participant data. Any performance reports, safety reports, or scholarly publications related to this study will omit participants' names or any other identifying information, and data will be submitted and described in consolidated/coded form.

All interviews with participants will be conducted by study personnel in a secure, private office at the UCSF Osher Center for Integrative Medicine. To further protect against loss of privacy, each individual who chooses to enroll in the study will be assigned a unique, randomly-generated identification number, and this number will be used on all documentation related to that participant throughout the entire study. The data key linking each participant with a unique study identification number will be kept in a separate, locked file cabinet, along with signed consent forms, next-of-kin contact information, and other protected health information. All participant data collected on paper forms during the study will be stored in a locked file cabinet within a locked office. Any electronic data collected during the study will maintained only on secure

office computers at the UCSF Osher Center for Integrative Medicine; both device-level encryption and file-level encryption will be applied to ensure electronic data security. Computers containing protected health information are physically locked to their workstations, and these workstations exist only in private, locked offices inaccessible to passersby. Furthermore, all computers containing protected health information have a password-protected screen lock, activated automatically during initial boot-up and during any periods when the computer is idle for more than 10 minutes.

3. What are the consequences to participants of a loss of privacy (e.g., risks to reputation, insurability, other social risks)?

Loss of privacy related to a participant's diagnosis of major depression may result in stigmatization in some social and vocational settings, as well as difficulty obtaining health insurance. To reduce the risk that participation in this research will result in such consequences, great care will be taken to protect the privacy of each participant, as outlined above.

The following questions address confidentiality issues:

4. Identifiers: Please indicate all identifiers that may be included in the research records for the study. Check all that apply.

[X] Names
[X] Dates
[X] Postal address
[X] Phone numbers
[ ] Fax numbers
[X] Email address
[X] Social Security Numbers*
[ ] Medical record numbers
[ ] Health plan numbers
[ ] Account numbers
[ ] License/Certificate numbers
[ ] Vehicle id numbers
[ ] Device identifiers/Serial numbers
[ ] Web URLs
[ ] IP address numbers
[ ] Biometric identifiers
[ ] Facial Photos/Images
[X] Any other unique identifier: next-of-kin contact for emergencies
[ ] None of the 18 identifiers listed above
*Required for studies conducted at the VA

5. Determining Whether HIPAA Regulations Apply to This Study: Please answer the questions below for the identifiers marked in the above section. Check all that apply:

Are study data:<br>[ ] Derived from a medical record? ***Please identify source***:<br>[ ] Added to the hospital or clinical medical record?<br>[ ] Created or collected as part of health care?<br>[ ] Used to make health care decisions?	**HIPAA regulations apply.**<br>The identifiers marked in section D.4 are PHI.
[X] Obtained from the participant, including interviews, questionnaires?<br>[ ] Obtained from a foreign country or countries only?<br>[ ] Obtained from records open to the public?<br>[ ] Obtained from existing research records?<br>[ ] None of the above.	**HIPAA regulations do not apply.**<br>The identifiers marked section D.4 are not PHI.

If HIPAA regulations apply, you are required to obtain individual participant authorization or a CHR-approved waiver of authorization, or both, to be allowed access to medical records. For the VA, use the SFVAMC authorization. (The one exception to these requirements is the use of a Limited Data Set along with a Data Use Agreement.)

6. Use and Disclosure of Personal Health Information: Please indicate to whom or where you may disclose any of the identifiers listed above as part of the study process. Check all that apply:

[X] We do not plan to share any of the personally identifying information listed above outside the research team.
[ ] The participant's medical record
[ ] The study sponsor: ***please indicate***:
[ ] The US Food & Drug Administration (FDA)
[ ] Others: ***please indicate***:
[ ] A Foreign Country or Countries

7. Data Security: Identifiable data should not be stored on laptops, PDA's or other portable devices. Please indicate how study data are kept secure. Check all that apply:

[X] Data are coded; data key is destroyed at end of study or ***provide date***:
[X] Data are coded; data key is kept separately and securely
[X] Data are kept in locked file cabinet
[X] Data are kept in locked office or suite
[X] Electronic data are protected with a password
[X] Data are stored on a secure network

8. Describe any additional steps taken to assure that identities of subjects and any of their health information which is protected under the law is kept confidential. If video or audio recordings will be made as part of the study, disposition of these recordings should be addressed here and in the consent form.

After signing informed consent, each participant who chooses to enroll in the study will be assigned a unique, randomly-generated identification number, and this number will be used on all documentation related to that participant throughout the entire study. All participant data collected on paper forms during the study will be stored in a locked file cabinet within a locked office. The data key linking each participant with a unique study identification number will be kept in a separate, locked file cabinet, along with signed consent forms, next-of-kin contact information, and other protected health information. Any electronic data collected during the study will maintained only on secure office computers at the UCSF Osher Center for Integrative Medicine; both device-level encryption and file-level encryption will be applied to ensure electronic data security. Computers containing protected health information are physically locked to their workstations, and these workstations exist only in private, locked offices inaccessible to passersby. Furthermore, all computers containing protected health information have a password-protected screen lock, activated automatically during initial boot-up and during any periods when the computer is idle for more than 10 minutes. Any performance reports, safety reports, or scholarly publications related to this study will omit participants' names or other identifying information, and data will be analyzed and submitted in consolidated/coded form.

**9.** Reportable Information: Is it reasonably foreseeable that the study will collect information that State or Federal law requires to be reported to other officials (e.g., child or elder abuse) or ethically requires action (e.g., suicidal ideation)? If "Yes," please explain below and include a discussion of the reporting requirements in the consent form.	[X]Yes [ ]No

All participants in this study will have a diagnosis of mild to moderate depression, and will be specifically asked about suicidal ideation or psychosis at several time-points throughout the study, including

- the physician evaluation conducted by the PI during the screening visit;
- the completion of the Beck Depression Inventory at the screening visit, at the baseline visit, and at assessment visits on Week 2, Week 4, Week 6 and Week 8;
- discussions or phone conversations that participants may have with the study physician concerning their mental health at any point in the study;
- discussions or phone conversations that participants may have with the yoga instructor concerning their mental health at any point in the study.

If, at any of these points, an individual participant reports suicidal ideation or psychotic symptoms, he or she will be evaluated by the study psychiatrist (the PI) for immediate withdrawal from the study and for referral to emergency psychiatric care as appropriate, including inpatient psychiatric hospitalization.

**E. Benefits:**<br>**1.** Are there potential direct benefits to study subjects? If "Yes," please describe below.	[X]Yes [ ]No

Based on previous short-term clinical trials suggesting significant anti-depressant effects of yoga (see Section D), potential direct benefits to participants in this proposed study include reduction of depression symptoms, although this is not guaranteed. In addition, all participants in this proposed study will have the opportunity to learn and practice classical yoga exercises in instructor-led group classes at no cost. Participants randomized to the classroom control will also have the opportunity to attend an in-depth educational course on the history and philosophy of yoga at no cost.

2. What are the potential benefits to society?

Major depression is the most disabling illness in the United States. Existing conventional treatments have significant drawbacks, including accessibility problems and disappointingly low remission rates coupled with high dropout rates. Patients often cannot complete treatment due to expense, duration of treatment, social/cultural stigma, or intolerable

medication side effects. If this proposed study supports the safety and efficacy of yoga in reducing symptoms of major depression, it offers intriguing possibilities for a non-pharmacological depression treatment associated with low cost, high accessibility, high social acceptance, and few risks. Such an intervention for major depression would be of enormous benefit to public health.

F. Risk/Benefit Analysis: How do the benefits of the study outweigh the risks to subjects?

Our proposed research project will recruit participants with mild to moderate depression, for whom conventional treatments for depression may be of uncertain benefit, and explore the effects of a yoga-based intervention for depression. Based on previous clinical trials suggesting anti-depressant effects of yoga, potential direct benefits to participants in this study include reduction of depression symptoms, although this is not guaranteed.

Effective physician screening will exclude participants who may be placed at serious psychological, physical, or reproductive risk due to pre-existing medical/psychiatric conditions or pregnancy. Effective physician screening will include a detailed medical and psychiatric evaluation, physical examination, and relevant urine pregnancy testing conducted with each participant prior to beginning the research protocol.

Participants in this study face some physical risks from performing the yoga exercises; these risks range from minor, transient muscle discomfort to more serious musculoskeletal injuries, as well as potential side effects of mild hyperventilation. To minimize these physical risks, all the yoga exercises selected for this study are low-impact and involve highly controlled movements. All yoga exercises will be taught and supervised by a skilled nurse-instructor. The nurse-instructor will take great care to emphasize to participants that they should not go beyond their usual range of motion/comfort for any of the yoga exercises. All yoga sessions will be held at the UCSF Osher Center for Integrative Medicine, where exam rooms, medical equipment, and physician back-up will be readily available to the nurse-instructor should any unexpected medical problems arise in the course of these sessions.

All participants with mild to moderate depression enrolled in this study face some risk that their depression may increase in severity over the 8-week course of the study. However, to protect against any adverse consequences arising from this risk, all participants will be closely monitored for any evidence of clinical decline over the study period. Clinical monitoring will include all of the following: (a) close observation and monitoring by the nurse-instructor during the intervention sessions with each participant, (b) formal assessment of depression severity every 2 weeks via the Beck Depression Inventory (BDI-II), (c) vigorous follow-up and assessment of any participant absenteeism, and (d) direct psychiatric assessment by the PI should a participant report subjective worsening of depression at any point in the study, should a participant endorse suicidal ideation or psychosis at any point in the study, or should a clinically significant increase occur between a participant's baseline BDI-II score and the BDI-II score at any subsequent assessment point. All participants will have direct phone access to the PI 24 hours a day, 7 days a week, to discuss any concerns about their depression. The PI is an experienced board-certified psychiatrist and will evaluate participants for immediate withdrawal from the study and for referral to appropriate, standard psychiatric care in the event of clinical decline from depression.

Given the overall safety measures in place for this study, we believe the risk/benefit ratio is positive.

PART 6: PARTICIPANT INFORMATION

**A. Number of Subjects:**	
**1.** How many subjects will be enrolled at UCSF and affiliated institutions?	40
**2.** How many subjects will be enrolled at all sites (i.e., if multicenter study)?	N/A
**3.** How many people do you estimate you will need to consent and screen here (but not necessarily enroll) to get the needed subjects?	Up to 150

**B. Types of Subjects**: Check all that apply. Click on links for additional instructions.	
[X]	Minors **Attach - Inclusion of Minors Supplement**
[ ]	Subjects unable to consent **Attach - Surrogate Consent or Emergency Waiver of Consent Supplement**
[ ]	Subjects with Diminished Capacity to Consent
[ ]	Subjects Unable to Read, Speak, or Understand English – **Complete Part 8.D of this application**
[ ]	Pregnant Women – **Complete Part 6.G of this application**
[ ]	Fetuses
[ ]	Neonates

[ ]	Prisoners **Attach - Inclusion of Prisoners Supplement**
[ ]	Inpatients
[X]	Outpatients
[ ]	Healthy Volunteers
[ ]	Staff of UCSF/affiliated institution

C. Eligibility Criteria:

1. General description of participant population(s):

For this research project, we aim to recruit a total of 40 Bay Area participants diagnosed with mild to moderate depression via structured psychiatric interview; 20 of these participants will be adolescents 14 to 17 years of age, while another 20 participants will be adults 18 years of age and older.

Based on epidemiological data showing that after age 13, major depression is more prevalent in girls and women, and that females are also more likely than males to seek help for depressive symptoms, we anticipate a predominance of girls and women in our study, who may represent about 60% of our participants. Since peak incidence of major depression among adolescents is between the ages of 15-18 years, we expect this adolescent age group to be most represented in our study. Since peak incidence of major depression among adults is between ages of 55-65 years, we expect this adult age group to be most represented in our study.

We also expect a wide range of ethnicities in our study population, since we will be recruiting participants from the ethnically diverse San Francisco Bay Area community. Based on clinic populations at the UCSF Medical Center, we anticipate that our subjects will be 54% European American, 33% Latino, 20% African American, 20% Asian American, 5% Pacific Islander, and 1% Native American. (Note that the total percentage exceeds 100% because individuals in the Latino category also belong to some of the other categories.)

2. Inclusion Criteria:

- Individuals 14 years of age and older
- Both male and female
- All ethnicities
- English proficiency sufficient for study participation
- Living near San Francisco during study, and able to attend all required visits for study participation
- If participant is 18 years of age or older, able to give voluntary, informed consent
- If participant is 14 -17 years of age, able to give assent and parents/legal guardian give voluntary, informed consent

- Clinical diagnosis of major depression per screening Mini International Neuro-psychiatric Interview (MINI)
- Depression symptoms of mild to moderate severity, per score of 14-28 on screening Beck Depression Inventory (BDI-II)

3. Exclusion Criteria:

- Use of antidepressant medication during the study period, or during the 2 months prior to study period
  (at screening, individuals who report being in treatment with antidepressant medication will ***not*** be asked to discontinue medication; instead, these individuals will be excluded from study participation)
- Use of psychotherapy during the study period
  (at screening, individuals who report being in psychotherapy will ***not*** be asked to discontinue psychotherapy; instead, these individuals will be excluded from study participation)
- Use of any yoga exercises, other than study intervention, during the study period

- Cognitive Impairment (score < 24 on screening Folstein Mental Status Exam)
- Diagnosis of current substance use disorders per screening psychiatric interview
- Diagnosis of bipolar disorder or any major Axis I anxiety disorders per screening psychiatric interview
- Severe major depression, per score > 28 on screening Beck Depression Inventory (BDI-II)
- Current suicidal ideation or past suicide attempts
- Current psychosis

- Pregnancy
- Seizure disorder

- Carotid artery stenosis
- Uncontrolled hypertension
- Severe pulmonary disease
- Severe musculoskeletal problems
- Other medical conditions ~~making study participation difficult~~ with ***acute*** somatic or constitutional symptoms (such as fever, vertigo, nausea, severe fatigue, severe pain) present at the time of screening and likely to interfere with 8 weeks of study participation.

D. How (chart review, additional tests/exams for study purposes), when and by whom will eligibility be determined?

The study PI will determine participant eligibility based on a structured psychiatric interview and detailed medical evaluation performed during the screening visit.

**E.** Are there any inclusion or exclusion criteria based on *gender*, *race* or *ethnicity*? If "Yes," please explain the nature and rationale for the restrictions below.	[ ]Yes [X]No

F. Populations Likely to be Vulnerable to Coercion or Undue Influence:

1. List participant groups who are likely to be vulnerable to coercion or undue influence, such as mentally disabled persons, economically or educationally disadvantaged persons, or investigators' staff or students. **Omit *minors*, *those unable to consent for themselves*, and *prisoners*** (who are covered by separate Supplements); **for *pregnant women, fetuses, and neonates*, see section G below**):

Participants in this study will be diagnosed with mild to moderate depression.

2. Explain why it is appropriate to include the groups listed above in this particular study:

Since this study is specifically investigating potential therapeutic efficacy of yoga as an intervention for depression, it is necessary that participants meet diagnostic criteria for depression.

3. Describe **additional** safeguards that have been included in the study to protect the rights and welfare of these subjects and minimize coercion or undue influence. For example, you might provide competence evaluations (specify) for the mentally disabled, payment amounts calibrated to be non=coercive for the financially disadvantaged, extra-careful evaluations of subjects' understanding of the study, advocates to be involved in the consent process, or use flyers to recruit subjects instead of directly approaching staff or students:

There will be no direct monetary payment to incentivize participation in this study for financially disadvantaged participants. Rigorous screening will be conducted by the PI, who is a board-certified psychiatrist, to ensure that participants' depressive symptoms are limited to mild to moderate severity and do not result in significant cognitive impairment or diminished capacity to make informed decisions.

G. Pregnant Women, Human Fetuses, and Neonates:
Identify all sections of 45 CFR 46 Subpart B (see Chart) under which you believe the research falls and provide study-specific information showing why the research falls within those sections:

Pregnant women will be excluded from study participation.

PART 7: RECRUITMENT

A. Please review CHR Recruitment Guidelines for more information about acceptable recruitment methods. Note that all advertisements, whether posted or broadcast, and all correspondence used for purposes of recruitment require CHR review and approval before they are used. Check all that apply:

[ ]	Study investigators recruit their own patients directly and/or nurses or staff working with researchers approach patients. ***Please explain in Section B.***
[X]	Study investigators send a CHR-approved letter to colleagues asking for referrals of eligible patients interested in the study. The investigators may provide the referring physicians a CHR-approved Information Sheet about the study to give to the patients. If interested, the patient will contact the PI. Or, with documented permission from the patient, the PI may be allowed to talk directly with patients about enrollment. ***Attach letter for review.***
[ ]	Study investigators provide their colleagues with a "Dear Patient" letter describing the study. This letter can be signed by the treating physicians and would inform the patients how to contact the study investigators. The study investigators may not have access to patient names and addresses for mailing. ***Attach letter for review.***
[X]	Advertisements, notices, and/or media used to recruit subjects. The CHR must first approve the text of these, and interested subjects will initiate contact with study investigators. ***Attach ads, notices, or media text for review. In Section B, please explain where ads will be posted.***
[ ]	Study investigators request a Waiver of Consent/Authorization for recruitment purposes. This waiver is an exception to the policy but may be requested in circumstances such as:
	[ ] Minimal risk studies in which subjects will not be contacted (i.e., chart review only);
	[ ] Review of charts is needed to identify prospective subjects who will then be contacted. (Explain in Waiver form);
	[ ] Large-scale epidemiological studies and/or other population-based studies when subjects may be contacted by someone other than personal physician. (Explain in Waiver form.)
[ ]	Direct contact of potential subjects who have previously given consent to be contacted for participation in research. Clinic or program develops a CHR-approved recruitment protocol that asks patients if they agree to be contacted for research (a recruitment database) or consent for future contact was documented using the consent form for another CHR-approved study. ***Please explain in Section B.***
[X]	Study investigators list the study on the UCSF Clinical Trials Seeking Volunteers web page or a similarly managed web site. Interested subjects initiate contact with investigators.
[ ]	Study investigators recruit potential subjects who are unknown to them. Examples include snowball sampling, use of social networks, direct approach in public situations, random digit dialing. ***Please explain in Section B.***

B. Provide detail in the space below (*i.e., how, when, where and by whom are potential subjects approached?*).

CHR-approved ads will be posted at Langley Porter adult and adolescent psychiatric outpatient clinics, UCSF Osher Center for Integrative Medicine, UCSF primary care outpatient clinics, SFGH community mental health clinics, SFGH primary care outpatient clinics, bulletin boards available for such ads at UCSF and SFGH medical centers, UCSF clinical trials websites, internet advertisement venues such as Craig's List, local newspapers, and public bulletin boards available for such ads at community yoga studios and health food stores.

PART 8: INFORMED CONSENT PROCESS

A. Check all that apply:

[X]	Signed consent will be obtained from subjects and/or parents (if subjects are minors),
[ ]	Verbal consent will be obtained from subjects, using an:
	[ ] Information sheet (attach)
	[ ] Script (attach)
[ ]	Signed consent will be obtained from surrogates **Attach - Surrogate Consent Supplement**
[ ]	Informed consent will not be obtained. **Attach - either the Waiver of Consent/Authorization or the Emergency Waiver of Consent Supplement as appropriate.**

B. In the space below, describe *how*, *where*, *when* and *by whom* informed consent will be obtained. How much time will prospective subjects be given to consider study participation? If special participant populations will be included, be sure to describe any additional plans for obtaining consent from particular populations. Justify any plans to use verbal consent instead of signed consent.

Participants are consented in person in a private office space before any study procedures are completed. They are consented by the PI. Participants are first given a copy of the consent form to read over. The PI then reviews the consent form with the participant and answers any questions or clarifies medical information for the participant. If a participant wishes to consider the study in more detail at home or to discuss the study with friends/family before signing, he/she will be given a copy of the consent form to take home, and he/she will be given another appointment time to meet with the PI.

**C.** How will you make sure subjects understand the information provided to them?
All individuals who choose to participate in the study will be required to provide written informed consent/assent prior to being enrolled. Participants (and parents, if the participant is a minor) will have the option to sign informed consent at the time of the initial screening interview or to return consent at a later date if they choose. Participants will be given a copy of the signed consent form and the UCSF Experimental Subject's Bill of Rights. Participants will have several opportunities at the time of the screening visit to ask questions and obtain clarification from the PI regarding study procedures.

**D. Subjects Who Do Not Read, Speak, or Understand English.**	
**1.** If you will enroll subjects who are unable to Read, Speak or Understand English, what method will you use to obtain consent? *Preferred Method* should be used if a substantial number of prospective subjects are expected to be non-English speakers. See **Those Who Do Not Read, Speak or Understand English** for details of methods.	
[ ]	*Preferred Method*—Consent form and other study documents will be available in the participant's primary language. Personnel able to discuss participation in the patient's language will be present for the consent process.
[ ]	*Short-Form*—A qualified interpreter will translate the consent form verbally, and subjects will be given the Experimental Participant's Bill of Rights in their primary language, following instructions in **Those Who Do Not Read, Speak or Understand English** for required witnessing and signatures.
**2.** How will you maintain the ability to communicate with non-English speakers throughout their participation in the study?	
We will be recruiting only English speakers for this study, as the principal research personnel do not have sufficient proficiency in other languages to recruit non-English speakers.	

PART 9: FINANCIAL CONSIDERATIONS

**A. Payments to Subjects:**<br>**1.** Will subjects receive payments or gifts for study participation?<br>If "Yes," please review CHR Participant Payment Guidelines and complete the following:	[X]Yes [ ]No
**2.** Payments will be (check all that apply):	[ ] Cash [ ] Check [X] Other (describe below) One free meditation class after screening visit, and 16 free yoga classes if enrolled in study
**3.** Please describe the schedule and amounts of payments, including the total subjects can receive for completing the study. If deviating from recommendations in Participant Payment Guidelines, include specific justification below.	
1) All participants completing the screening visit will receive a voucher for attendance at one free meditation class at the UCSF Osher Center for Integrative Center.<br>2) At the conclusion of the study, all participants randomized to the control intervention will have the opportunity to learn the active yoga intervention by attending 16 free group yoga classes at the Osher Center for Integrative Medicine.	

**B. Costs to Subjects:** Will subjects or their insurance be charged for any study procedures? If "Yes," describe those costs below, and compare subjects' costs to the costs associated with alternative care off-study. Finally, explain why it is appropriate to charge those costs to the subjects.	[ ]Yes [X]No

C. Treatment and Compensation for Injury: The investigators are familiar with and will follow the University of California policy and (if applicable) Veteran's Affairs policy regarding treatment and compensation for injury. If subjects are injured as a result of being in this study, treatment will be available. The costs of such treatment may be covered by the University of California, by the Department of Veteran's Affairs (for subjects eligible for veteran's benefits, if the SF VAMC is a study site), or by the study sponsor, if any, depending on a number of factors. The University does not normally provide any other form of compensation for injury.

PART 10: BIBLIOGRAPHY

1. Murray CJL, Lopez AD (Eds). The global burden of disease: a comprehensive assessment of mortality and disability from diseases, injuries, and risk factors in 1990 and projected to 2020. Cambridge, MA: Harvard Univ Press, 1996. Published on behalf of the World Health Organization and the World Bank.

2. National Healthcare Quality Report: Summary. December 2003. AHRQ Publication No. 04-RG003. Agency for Healthcare Research and Quality, Rockville, MD. http://www.ahrq.gov/qual/nhqr03/nhqrsum03.htm

3. Miniño AM, Arias E, Kochanek KD, Murphy SL, Smith BL. Deaths: final data for 2000. National Vital Statistics Reports, 50(15). Hyattsville, MD: National Center for Health Statistics, 2002.

4. Angst J, Angst F, Stassen HH. (1999). Suicide risk in patients with major depressive disorder. Journal of Clinical Psychiatry, 60 (Suppl. 2), 57–62.

5. Gonzalez MB, Snyderman TB, Colket JT, Arias RM, Jiang JW, O'Connor CM, Krishnan KR. Depression in patients with coronary artery disease. Depression. 1996;4(2):57-62.

6. Glassman AH, Shapiro PA. Depression and the course of coronary artery disease. Am J Psychiatry. 1998 Jan;155(1):4-11.

7. Katon WJ, Rutter C, Simon G, Lin EH, Ludman E, Ciechanowski P, Kinder L, Young B, Von Korff M. The association of comorbid depression with mortality in patients with type 2 diabetes. Diabetes Care. 2005;28(11):2668–72.

8. Zhang X, Norris SL, Gregg EW, Cheng YJ, Beckles G, Kahn HS. Depressive symptoms and mortality among persons with and without diabetes. Am J Epidemiol. 2005;161:652-660.

9. Schulberg HC, Katon W, Simon GE, Rush AJ. Treating major depression in primary care practice: an update of the Agency for Health Care Policy and Research Practice Guidelines. Arch Gen Psychiatry. 1998 Dec;55(12):1121-7.

10. Mulrow CD, Williams JW, Jr., Trivedi M, et al. Treatment of Depression: Newer Pharmacotherapies. Evidence Report/Technology Assessment No. 7. (Prepared by the San Antonio Evidence-based Practice Center based at The University of Texas Health Science Center at San Antonio under Contract 290-97-0012.) AHCPR Publication No. 99-E014. Rockville, MD: Agency for Health Care Policy and Research. February 1999

11. Ferrier IN. Treatment of major depression: is improvement enough? J Clin Psychiatry. 1999;60 Suppl 6:10-4.

12. Quitkin FM, Rabkin JG, Gerald J, Davis JM, Klein DF. Validity of clinical trials of antidepressants. Am J Psychiatry. 2000 Mar;157 (3):327-37.

13. Wells KB, Sturm, Sherbourne C, Meredith L. 1996. Caring for Depression. Cambridge: Harvard University Press.

14. Thase ME. Effectiveness of antidepressants: comparative remission rates. J Clin Psychiatry. 2003;64 Suppl 2:3-7.

15. Nierenberg AA, Wright EC. Evolution of remission as the new standard in the treatment of depression. J Clin Psychiatry. 1999;60 Suppl 22:7-11.
16. Lam RW, Kennedy SH. Evidence-based strategies for achieving and sustaining full remission in depression: focus on metaanalyses. Can J Psychiatry. 2004 Mar; 49 (Suppl 1):17S-26S.

17. Hirschfeld R, Keller M, Panico S, Arons B, Barlow D, Davidoff F, and others. The National Depressive and Manic-Depressive Association consensus statement on the undertreatment of depression. JAMA 1997;277:333–40.

18. Van Voorhees BW, Cooper LA, Rost KM, Nutting P, Rubenstein LV, Meredith L, Wang NY, Ford DE. Primary care patients with depression are less accepting of treatment than those seen by mental health specialists. J Intern Med. 2003 Dec; 18(12):991-1000.

19. Edlund MJ, Wang PS, Berglund PA, Katz SJ, Lin E, Kessler RC. Dropping out of mental health treatment: patterns and predictors among epidemiological survey respondents in the U.S. and Ontario. Am J Psychiatry. 2002 May;159(5):845-51.

20. Lyons D, McLoughlin DM. Recent advances: Psychiatry. BMJ. 2001 Nov 24; 323(7323):1228-31.

21. Sirey JA, Bruce ML, Alexopoulos GS, Perlick DA, Raue P, Friedman SJ, Meyers BS. Perceived stigma as a predictor of treatment discontinuation in young and older outpatients with depression. Am J Psychiatry. 2001 Mar;158(3):479-81.

22. Wierzbicki, M., and Pekarik, G. (1993). A meta-analysis of outpatient therapy dropout studies. Professional Psychology: Research and Practice, 24, 190-195.

23. Hammad TA, Laughren T, Racoosin J. Suicidality in pediatric patients treated with antidepressant drugs. Arch Gen Psychiatry. 2006 Mar;63(3):332-9.

24. Leon AC. The revised warning for antidepressants and suicidality: unveiling the black box of statistical analyses. Am J Psychiatry. 2007 Dec;164(12):1786-9.

25. Bachar E. Psychotherapy--an active agent: assessing the effectiveness of psychotherapy and its curative factors. Isr J Psychiatry Relat Sci. 1998;35(2):128-35.

26. DeRubeis RJ, Hollon SD, Amsterdam JD, Shelton RC, Young PR, Salomon RM, O'Reardon JP, Lovett ML, Gladis MM, Brown LL, Gallop R. Cognitive therapy vs medications in the treatment of moderate to severe depression. Arch Gen Psychiatry. 2005 Apr;62(4):409-16.

27. Brown GS, Lambert MJ, Jones ER, Minami T. Identifying highly effective psychotherapists in a managed care environment. Am J Manag Care. 2005Aug;11(8):513-20.

28. Mohl PC, Martinez D, Ticknor C, Huang M, Cordell L. Early dropouts from psychotherapy. J Nerv Ment Dis. 1991 Aug;179 (8):478-81.

29. Ogrodniczuk JS, Joyce AS, Piper WE. Strategies for reducing patient-initiated premature termination of psychotherapy. Harv Rev Psych. 2005 Mar-Apr;13(2):57-70.

30. Piper, W.E., Ogrodniczuk, JS, Joyce, AS, McCallum, M, Rosie, JS, O'Kelly, JG, & Steinberg, PI (1999). Prediction of dropping out in time-limited, interpretive individual psychotherapy. Psychotherapy, 36, 114-122.

31. Wong EC, Kim BS, Zane NW, Kim IJ, Huang JS. Examining culturally based variables associated with ethnicity. Cultur Divers Ethnic Minor Psychol. 2003 Feb;9(1):88-96.

32. Kozuki Y, Kennedy MG. Cultural incommensurability in psychodynamic psychotherapy in Western and Japanese traditions. J Nurs Scholarsh. 2004;36(1):30-8.

33. Wen-Shing Tseng, Jon Streltzer, Editors Culture and Psychotherapy: A Guide to Clinical Practice. 2001 American Psychiatric Press, Inc.

34. Segal Z, Vincent P, Levitt A. Efficacy of combined, sequential and crossover psychotherapy and pharmacotherapy in improving outcomes in depression. J Psychiatry Neurosci. 2002 Jul;27(4):281-90.

35. Pampallona S, Bollini P, Tibaldi G, Kupelnick B, Munizza C. Combined pharmacotherapy and psychological treatment for depression: a systematic review. Arch Gen Psychiatry. 2004 Jul; 61(7):714-9.

36. Thase ME, Jindal RD. Combining psychotherapy and psychopharmacology for treatment of mental disorders. In: Lambert MJ, editor. Bergin and Garfield's Handbook of psychotherapy and behavior change. 5th ed. New York: John Wiley and Sons Inc; 2004. Pages 743–66.

37. Hegerl U, Plattner A, Moller HJ. Should combined pharmaco- and psychotherapy be offered to depressed patients? A qualitative review of randomized clinical trials from the 1990s. Eur Arch Psychiatry Clin Neurosci. 2004 Apr; 254(2):99-107.

38. March J, Silva S, Petrycki S, Curry J, Wells K, Fairbank J, Burns B, Domino M, McNulty S, Vitiello B, Severe J; Treatment for Adolescents With Depression Study (TADS) Team. Fluoxetine, cognitive-behavioral therapy, and their combination for adolescents with depression: TADS randomized controlled trial. JAMA. 2004 Aug 18; 292(7):807-20.

39. Goodyer IM, Dubicka B, Wilkinson P, Kelvin R, Roberts C, Byford S, Breen S, Ford C, Barrett B, Leech A, Rothwell J, White L, Harrington R. A randomised controlled trial of cognitive behaviour therapy in adolescents with major depression treated by selective serotonin reuptake inhibitors. The ADAPT trial. Health Technol Assess. 2008 May; 12(14):iii-iv, ix-60.

40. Brent D, Emslie G, Clarke G, Wagner KD, Asarnow JR, Keller M, Vitiello B, Ritz L, Iyengar S, Abebe K, Birmaher B, Ryan N, Kennard B, Hughes C, DeBar L, McCracken J, Strober M, Suddath R, Spirito A, Leonard H, Melhem N, Porta G, Onorato M, Zelazny J. Switching to another SSRI or to venlafaxine with or without cognitive behavioral therapy for adolescents with SSRI-resistant depression: the TORDIA randomized controlled trial. JAMA. 2008 Feb 27; 299(8):901-13.

41. Kessler RC, Berglund P, Demler O, Jin R, Koretz D, Merikangas KR, Rush AJ, Walters EE, Wang PS, National Comorbidity Survey Replication. The epidemiology of major depressive disorder: results from the National Comorbidity Survey Replication (NCS-R). JAMA. 2003;289(23):3095-3105.

42. Cassano P, Fava M. Depression and public health: an overview. J Psychosom Res. 2002 Oct;53(4):849-57.

43. Collins KA, Westra HA, Dozois DJ, Burns DD. Gaps in accessing treatment for anxiety and depression: challenges for the delivery of care. Clin Psychol Rev. 2004 Sep;24(5):583-616.

44. Eisenberg DM, Davis RB, Ettner SL, Appel S, Wilkey S, Van Rompay M, Kessler RC. Trends in alternative medicine use in the U.S, 1990-1997: results of a follow-up national survey. JAMA. 1998 Nov 11;280(18):1569-75

45. Wang JL, Patten SB, Russell ML. Alternative medicine use by individuals with major depression. Can J Psychiatry. 2001 Aug;46(6):528-33

46. Kessler RC, Soukup J, Davis RB, Foster DF, Wilkey SA, Van Rompay MI MI, Eisenberg DM. The use of complementary and alternative therapies to treat anxiety and depression in the United States. Am J Psychiatry. 2001 Feb;158 (2):289-94.

47. Unutzer J, Klap R, Sturm R, Young AS, Marmon T, Shatkin J, Wells KB. Mental disorders and the use of alternative medicine: results from a national survey. Am J Psychiatry. 2000 Nov;157 (11):1851-7.

48. Tindle HA, Davis RB, Phillips RS, Eisenberg DM. Trends in use of complementary and alternative medicine by US adults: 1997-2002. Alt Ther Health Med. 2005 Jan-Feb;11(1):42-9.

49. Pilkington K, Kirkwood G, Rampes H, Richardson J. Yoga for depression: the research evidence. J Affect Disord. 2005 Dec; 89(1-3):13-24. Epub 2005 Sep 26.

50. Woolery A, Myers H, Sternlieb B, Zeltzer L. A yoga intervention for young adults with elevated symptoms depression. Altern Ther Health Med. 2004 Mar-Apr;10(2):60-3.

51. Rohini V, Pandey RS, Janakiramaiah N, Gangadhar BN, Vedamurthachar A. A comparative study of full and partial Sudarshan Kriya Yoga in major depressive disorder. NIMHANS Journal, 2000; 18: 53-57.

52. Janakiramaiah N, Gangadhar BN et al. Antidepressant efficacy of sudarshan kriya yoga (SKY) in melancholia: a randomized comparison with ECT and imipramine. Journal of Affective Disorders, 2000; 57: 255-259.

53. Khumar SS, Kaur P, Kaur MS. Effectiveness of Shavasana on depression among university students. Indian J Clin Psych 1993;20(2):82-87.

54. Broota A, Dhir R. Efficacy of two relaxation techniques in depression. J Pers Clin Stud 1990;6:83-90.

55. Spence, J.C., Poon, P., Dyck, P. (1997). The effect of physical-activity participation on self-concept: A meta-analysis (Abstract). Journal of Sport and Exercise Psychology, 19, S109.

56. Milligan RA, Burke V, Beilin LJ, Richards J, Dunbar D, Spencer M, Balde E, Gracey MP. Health-related behaviours and psycho-social characteristics of 18 year-old Australians.
Soc Sci Med. 1997 Nov;45(10):1549-62.

57. Lee SW, Mancuso CA, Charlson ME. Prospective study of new participants in a community-based mind-body training program. J Gen Intern Med. 2004 Jul;19(7):760-5.

58. Anderson RT, King A, Stewart AL, Camacho F, Rejeski WJ. Physical activity counseling in primary care and patient well-being:Do patients benefit? Ann Behav Med. 2005 Oct;30(2):146-54

59. Piko BF, Keresztes N. Physical activity, psychosocial health, and life goals among youth. J Community

Health. 2006 Apr;31(2):136-45.

60. Brown SW, Welsh MC, Labbé EE, Vitulli WF, Kulkarni P. Aerobic exercise in the psychological treatment of adolescents. Percept Mot Skills. 1992 Apr;74(2):555-60.

61. Craft LL, Perna FM. The Benefits of Exercise for the Clinically Depressed. Prim Care Companion J Clin Psychiatry. 2004;6(3):104-111.

62. Daley AJ, Copeland RJ, Wright NP, Roalfe A, Wales JK. Exercise therapy as a treatment for psychopathologic conditions in obese and morbidly obese adolescents: a randomized, controlled trial. Pediatrics. 2006 Nov;118(5):2126-34.

63. Shin KR, Kang Y, Park HJ, Heitkemper M. Effects of exercise program on physical fitness, depression, and self-efficacy of low-income elderly women in South Korea. Pub Health Nurs. 2009 Nov-Dec;26(6):523-31.

64. Prathikanti S, Wolkowitz O, Fernandes R. Treating Depression with Yoga Breathing: an Open-label Feasibility Trial at UCSF. Unpublished data; 2005

65. Nagarathana, R, Nagendra, HR. Yoga Practices for Anxiety and Depression. Bangalore: Swami Vivekananda Yoga Prakashana; 2004.

66. Khan A, Warner HA, Brown WA. Symptom reduction and suicide risk in patients treated with placebo in antidepressant clinical trials. Arch Gen Psychiatry 2000; 57:311-7.

67. Storosum JG, van Zweiten BJ, van den Brink W, Gersons BPR, Broekmans AW. Suicide risk in placebo-controlled studies of major depression. Am J Psychiatry 2001;158:1271-5.

68. Hammad T, Laughren T, Racoosin JA. Suicide rates in short-term randomized controlled trials of newer antidepressants. J Clin Psychiatry 2006;26:203-7.

69. Stone M, Laughren T, Jones ML, Levenson M, Holland PC, Hughes A, Hammad TA, Temple R, Rochester G. Risk of suicidality in clinical trials of antidepressants in adults: analysis of proprietary data submitted to US Food and Drug Administration. BMJ. 2009 Aug 11;339:b2880.

70. Wang PS, Lane M, Olfson M, Pincus HA, Wells KB, Kessler RC. Twelve-month use of mental health services in the United States: results from the National Comorbidity Survey Replication. Arch Gen Psychiatry. 2005 Jun;62(6):629-40.

71. Pratt LA, Brody DJ. Depression in the United States household population, 2005-2006. Centers for Disease Control and Prevention National Center for Health Statistics Data Brief. 2008 Sep;(7):1-8.

72. González HM, Vega WA, Williams DR, Tarraf W, West BT, Neighbors HW. Arch Gen Psychiatry. 2010 Jan;67(1):37-46. Depression care in the United States: too little for too few.

73. Fournier, JC; Robert J. DeRubeis, RJ; Hollon, SD; Dimidjian, S; Amsterdam, JD; Shelton, RC; Fawcett, J. Antidepressant Drug Effects and Depression Severity: A Patient-Level Meta-analysis. JAMA. 2010;303(1):47-53.

74. Kirsch I, Deacon BJ, Huedo-Medina TB, Scoboria A, Moore TJ, Johnson BT. Initial severity and antidepressant benefits: a meta-analysis of data submitted to the FDA. PLoS Med. 2008 Feb;5(2):e45.

PART 11: ATTACHMENTS

Please list Attachments, Supplements and Appendices	Version number(s) or date(s)
Application Supplement: Inclusion Of Children And Minors In Research	3/12/10
Appendix A: Informed Consent Form	3/12/10
Appendix B: Telephone Screening Form	3/12/10
Appendix C: Physician Evaluation Form	3/12/10
Appendix D: Final Feedback Questionnaire	3/12/10
Appendix E: Recruitment Flyer	3/12/10
Appendix F: Recruitment Ad for online /print classifieds & for attachment to Dear Colleague Letter	3/12/10
Appendix G: Recruitment Ad for UCSF clinical trials website	3/12/10
Appendix H: Dear Colleague Recruitment Letter	3/12/10
