# Supplementary material for: Treating major depression with yoga: A prospective, randomized, controlled pilot trial
Source: PLoS One. 2017 Mar 16;12(3):e0173869. doi: 10.1371/journal.pone.0173869 (PMC5354384; doi:10.1371/journal.pone.0173869)
Supplement: S3 File — (PDF) [file pone.0173869.s003.pdf]

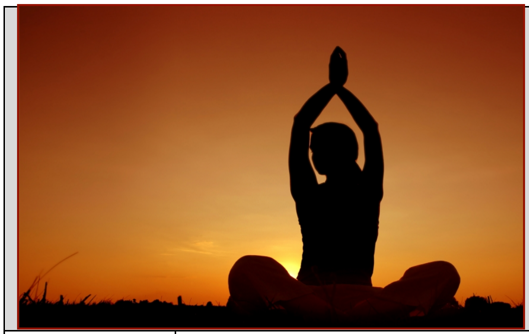

Treating Depression with Yoga Breathing: An Open-label Feasibility Study

Conducted at UCSF Bridge to Wellness Program

By Fernandes R, Prathikanti S

Poster Session
National Institute of Mental Health
Summer Training On Aging Research Topics In Mental Health
August 2005, San Diego, California

**Aims**	Assess recruitment response rate & enrollment rate of U.S. subjects in a study of yoga for depression<br>Assess attrition & adherence rates of depressed U.S. subjects in a time-intensive yoga-based intervention<br>Assess whether subjects practicing yoga intervention exhibit reduction in depressive symptoms
**Subjects**	Adults 18 and older interested in learning a yoga practice that may help reduce depressive symptoms<br>Consecutive sampling of outpatients recruited thru two UCSF outpatient clinics via flyers<br>Major depression diagnosed with Mini International Neuropsychiatric Interview<br>Baseline score on Hamilton Rating Scale for Depression (HRSD) >13<br>Baseline score on Beck Depression Inventory-II (BDI) >13<br>Not taking antidepressant medication OR Stable dose of antidepressant medication for > 2 months<br>No participation in any other yoga or mind-body practice during the study<br>No acute psychosis or suicidal ideation
**Intervention**	Daily 30-minute home practice of 4 yoga breathing exercises (with audiotape) and<br>Weekly 60-minute instructor-led group practice of 4 yoga breathing exercises (see attached)
**Duration**	Total duration of study: 4 weeks
**Outcome Measures**	Total number of people responding to recruitment ads over a one month period<br>Total number of subjects enrolled in the study over a one month period<br>Percentage of assigned intervention sessions completed by subjects<br>Change in mean scores on Beck Depression Inventory (BDI) pre- and post-intervention<br>Change in mean scores on Hamilton Rating Scale for Depression (HRSD) pre- and post-intervention

Results

Recruitment response rate:	42 subjects/month
Enrollment rate:	12 subjects/month (Female: 9; Age: 28 to 61 yrs)
Concurrent antidepressant medication use	9 subjects
Total Attrition:	3 subjects
Mean percentage Group Practice sessions completed:	68%
Mean percentage Home Practice sessions completed:	63% (home data was missing on 3 subjects)

At conclusion of study, 75% of subjects indicated willingness to attend 3 group practices/wk at research site

Pre-intervention to post-intervention change in mean BDI scores and in mean HRSD scores were both statistically significant, supporting potential antidepressant benefits of yoga in this sample:

	**Pre-Intervention N=12**	**Post-Intervention N=10**
Mean BDI	28.67	17.90
SD	12.61	13.82
SEM	3.64	4.37

Change in Mean BDI scores = 11.10
95% confidence interval: 1.41 to 20.79
Paired t-test, 2-tailed α=0.05: P value = 0.029

	**Pre-Intervention N=12**	**Post-Intervention N=9**
Mean HRSD	25.25	13.67
SD	6.00	6.40
SEM	1.73	2.13

Change in Mean HRSD scores = 11.22
95% confidence interval: 4.46 to 17.99
Paired t-test, 2-tailed α=0.05: P value= 0.005

TREATING DEPRESSION WITH YOGA BREATHING: AN OPEN-LABEL FEASIBILITY STUDY

Breathing Practice	Instructions
**ALTERNATE NOSTRIL BREATHING**<br>(Nadi Shodana)	1. Sit upright on a straight-back chair, with both feet planted firmly on the ground. Rest your left hand on your belly and your right hand on your leg. Close your eyes.<br>2. Bring your right hand to your face and gently press the right nostril shut with your thumb.<br>3. Take a deep breath in, feeling the flow of air through your left nostril. As you inhale, count to 4, allowing your belly to expand first, and then your chest.<br>4. Pause for a count of 2<br>5. Release the right nostril, pressing the left nostril shut with your index finger. Exhale through right nostril, counting to 4 and allowing your chest to relax first, and then your belly.<br>6. Pause for a count of 2<br>7. Now take a deep breath in through right nostril. As you inhale, count to 4, allowing your belly to expand first, and then your chest.<br>8. Pause for a count of 2<br>9. Release your left nostril, pressing the right nostril shut with your thumb. Exhale through left nostril, counting to 4 and allowing your chest to relax first, and then your belly.<br>10. Pause for a count of 2<br>11. Continue to breathe in this manner for 3 minutes, until you hear the bell ring.
**VICTORIOUS BREATH**<br>(Ujjayi)	1. Sit on a straight-back chair. Back and neck should be upright and both feet planted on ground. Close your eyes. Clasp your hands in front of your chest, pointing the index fingers upward.<br>2. Exhale through the nostrils emptying your lungs and abdomen of air.<br>3. Slightly contract the throat (glottis) and breathe in deeply through the nostrils. This will make a gentle snoring sound. You may feel some vibration in the back of your throat.<br>4. As you inhale, move your clasped hands above your head, counting to 4 as your belly and your chest to fill with air.<br>5. Pause for a count of 2.<br>6. Exhale slowly through your nostrils, continuing to contract the throat slightly.<br>7. As you exhale, lower your clasped hands back to chest, counting to 4 as belly and chest relax.<br>8. Pause for a count of 2.<br>9. Breathe as slowly as you can in this manner for 8 minutes, until you hear the bell ring.
**BELLOWS BREATH**<br>(Bhastrika)	1. Sit on a straightback chair. Back and neck should be upright and both feet planted on ground. Close your eyes. Curl fingers into fists and place fists next to shoulders, thumb-side to shoulder.<br>2. Take a deep breath in through your nostrils, counting to one and moving hands straight up and unfurling your fingers.<br>3. Exhale rapidly, counting to one and moving hands back alongside the shoulders and curling fingers back into a fist.<br>4. Continue to breathe forcefully in this manner for 8 minutes, until you hear the bell ring.
**BUMBLEBEE BREATH**<br>(Bhramari)	1. Sit on a straightback chair. Back and neck should be upright and both feet should be planted firmly on the ground. Close your eyes, resting your hands on the legs.<br>2. Gently bring your hands to your face, placing the index fingers over each eyebrow and gently resting the remaining fingers over each eye.<br>3. Block your ears by using the thumbs to close the flap in front of each ear.<br>4. Inhale deeply through both nostrils, allowing your belly to expand first, and then your chest. Count to 4 as you inhale.<br>5. Pause for a count of 2<br>6. Exhale smoothly through the nostrils and produce a gentle, continuous humming sound until the exhalation is complete. Count to 8 as you exhale.<br>7. Pause for a count of 2 after you exhale.<br>8. Continue to breathe in this manner for 8 minutes, until you hear the bell ring
