# Supplementary material for: Treating major depression with yoga: A prospective, randomized, controlled pilot trial
Source: PLoS One. 2017 Mar 16;12(3):e0173869. doi: 10.1371/journal.pone.0173869 (PMC5354384; doi:10.1371/journal.pone.0173869)
Supplement: S4 File — (PDF) [file pone.0173869.s004.pdf]

EDUCATIONAL MODULES: YOGA HISTORY & PHILOSOPHY

FORMAT FOR EACH SESSION:

1. Check attendance and briefly introduce new participants.
2. Present educational module on yoga history or philosophy, using the specific book chapter or adapted narrative provided for each module.
3. Pose 3 or 4 questions to consider for the film segment
4. Show selected film.
5. Facilitate discussion, with participants sharing responses to questions posed at start of session.
6. Close by reminding participants to notify staff re: any concerns about mental health status.

SESSION 1
Educational Module: "Essence of Yoga."
Narrative adapted from The Yoga Tradition by Georg Feuerstein [1]

Questions for Film Segments:
1) As you listen to different speakers in film, which definition of yoga resonates most with you? Why?
2) What ancient civilization gave rise to yoga, and where was it located geographically?
3) Name three ancient texts that provide some of the basis for yoga philosophy.
4) What aspect of yoga history was the most surprising or striking to you as you watched this film?

Film [2]: Yoga Unveiled (Disc One) Part 1. What is Yoga?
The film opens with a segment on the contemporary practice of yoga in the West. The experts define yoga in their own words.

Film [2]: Yoga Unveiled (Disc One) Part 2. Origins of Yoga
This segment covers the Indus Valley civilization, the Vedas, the Upanishads, the Bhagavad Gita, and the Yoga Sutras. Includes: An overview of the formation of the yogic philosophy in ancient India. Extensive commentary by Georg Feuerstein, Subhash Kak, Edwin Bryant, and other leading yoga scholars. Includes archival footage of the excavation of the Indus Valley Civilization, the birthplace of yoga.

SESSION 2
Educational Module: "The Wheel of Yoga."
Narrative adapted from The Yoga Tradition by Georg Feuerstein [1]

Questions for Film Segment:
1) What is the ultimate goal of yoga, from the standpoint of the classical yoga masters?
2) Name at least 3 different "paths" within the yoga tradition, and personality traits particularly suited for each.
3) What are the eight limbs of classical yoga, as described by Patanjali?
4) Which yoga master or scholar in this segment made the deepest impression on you, and why?

Film [2]: Yoga Unveiled (Disc One) Part 3. The Branches of Yoga

This segment begins with explanations of bhakti yoga, karma yoga, jnana yoga, and raja (ashtanga) yoga. Then there's a discussion of yogic views of the human body/mind followed by a brief discussion of tantra and a rather abstract discussion of kundalini yoga. Next, there is a detailed exploration of hatha yoga. The great modern masters and scholars share their insights on each branch. Film segment includes rare archival footage of the great Swami Sivananda.

SESSION 3
Educational Module: "Modern Yoga."
Narrative adapted from The Yoga Tradition by Georg Feuerstein [1]

Questions for Film Segment:
1) What was the significance of Swami Vivekananda to the yoga movement in the United States?
2) What aspect of Krishnamacharya's life, as presented in this film, was the most striking to you?
3) Name the four major students of Krishnamacharya who brought yoga to the West, and describe one specific contribution to modern yoga that each student made.

Film [2]: Yoga Unveiled: Disc One. Part 4. The Passage from East to West
This segment covers the Asiatic Society, the Transcendentalists, the 1893 World's Parliament of Religions in Chicago, and Swami Vivekananda, who introduced yoga to America. Includes old photos, newspaper reviews, and Swami Vivekananda's speeches at the Parliament of Religions in Chicago.

Film [2]: Yoga Unveiled: Disc One. Part 5. Modern Yoga
This segment profiles five teachers who were instrumental in reviving and shaping hatha yoga in the twentieth century and introducing it to the West: Krishnamacharya, Indra Devi, Iyengar, Pattabhi Jois, and Desikachar. This segment will be of special interest to yoga students who've already heard of some of these teachers. Includes old footage of Krishnamacharya, who preserved the ancient tradition of yoga and trained the greatest modern yoga masters. Contains insightful commentary about the place of yoga in modern world

SESSION 4
Educational Module: "Role of the Teacher."
Narrative adapted from The Yoga Tradition by Georg Feuerstein [1]

Questions for Film Segment :
1) Which yogi in the film was the most difficult for you to relate to? Why?
2) Which yogi in the film was the most inspirational to you? Why?
3) The teacher-student relationship is considered very important in the classical yoga tradition. Do you think this relationship is still important to maintain in the modern era? Why or why not?

Film [3]: Origins of Yoga: Quest for the Spiritual
Yoga is often seen as an exercise regime, yet it originated as a path to the inner self, and the physical benefits of yoga are just a small part of its benefits. The path of yoga is shrouded in antiquity - when Himalayan sages probed the deepest mysteries of life -- and what lies beyond death. In this film, the Yogis of India themselves are interviewed on location as they describe their quest, their lifestyle, and the true meaning of Yoga.

SESSION 5
Educational Module: "Yoga for Health."
Narrative adapted from *Ayurveda and Mental Health*, by Sudha Prathikanti [4]

Questions for Film Segment:
1) What are some reasons that people in the west, including yourself, may be interested in using yoga for the treatment of various medical conditions?
2) How do you think the ancient founders of yoga might have viewed the notion of yoga as a healthcare modality?
3) Which modern doctor or health care provider in this film segment is most inspiring to you, and why?

Film [2]: Yoga Unveiled: Disc Two. Part 6. Yoga and Modern Medicine
This segment explores yoga's uses for physical and mental health and healing and the integration of yoga with Western medicine. Contains interviews with the pioneers in the integrative medicine field, including Dr. Dean Ornish, Jon Kabat-Zinn, Dr. Herbert Benson, Dr. Mehmet Oz, Father Joe Pereira, among others.

SESSION 6
Educational Module: “Kumbha Mela.”
Narrative adapted from Kumbha Mela by Jack Hebner and David Osborn [5]

Questions for Film Segment:
1) In what four cities is the Kumba Mela held, and what is the significance of these four places according to the legends describing the origin of the Kumbha Mela?
2) Name two groups of ascetics (sadhus) who participate in the main procession at the Kumbha Mela, and briefly describe the main practices of these groups.
3) Which sadhu in this film made the strongest impression on you, and why?

Film [6]: Shortcut to Nirvana (Selected 45 minutes)
The Kumbh Mela is the biggest gathering of people in the history of humanity – although few in the West have ever heard of it. More than 70 million pilgrims attend this extraordinary spiritual festival, which has been held every 12 years near Allahabad, India, for over two millennia. A vast tent city is established to accommodate the masses, and many of India’s greatest gurus and spiritual leaders set up camp to give discourses to their devotees. On the main bathing day, more than 25 million people bathe in the sacred waters. This single act is believed to secure release from the endless cycle of rebirth – literally a short cut to the state of purest bliss: nirvana. This film takes a voyage of discovery through this vivid and vibrant world, accompanied by a young Hindu monk, Swami Krishnanand, and several Westerners, each on their own spiritual quest.

SESSION 7
Educational Module: “Reaching Beyond the Ego Personality”
Narrative adapted from The Yoga Tradition by Georg Feuerstein [1]

Questions for Film Segment:
1) According to this film, how may yoga enhance one’s personal growth, regardless of one’s religion or culture?
2) Which Indian yoga guru and which western yoga guru made the deepest impressions on you? Why?
3) In this film, to what extent have modern yoga gurus kept the traditional message of yoga the same, and in what ways have they changed it?

Film [7]: The Yoga Gurus (Selected 45 minutes)
Come and meet the top yoga teachers from east and west. Go beyond the physical asanas and deep into the mind and inner being of a yoga guru. In this film, listen to the words of wisdom from these yoga teachers:

HH Swami Chidanand is one of India’s most revered saints and spiritual leaders, and the founder of Parmarth Niketan, a major Yoga Ashram in Rishikesh. He teaches “mind, body, spirit and soul yoga.”
Sharath is the grandson of Pattabhi Jois, one of Yoga’s most important teachers. He runs the Ashtanga school in Mysore and travels the world teaching.
Yogacharya Venkatesha is another leading Yoga teacher in Mysore, India. A Hatha Yoga master, his school is very popular with foreign students.
Gurmukh is America’s top Kundalini master and runs the largest Yoga school in the world with 5,000 students. Her style and brand of teaching is unique, her insight and wisdom is worthy of a movie by itself.
Dr David Frawley is a leading author and a recognized expert in Vedic and Yoga teaching. He has received major awards and accolades in India for his work.
Shyamdas is musician, scholar and Bhakti Yoga master of the devotional practice. With many books and music CD’s to his credit, he is sought after by leading Yoga institutions all over the world for lectures.
Gabriela Bozic runs a large Jivamukti Yoga center in Germany. She is a leading expert in this hatha style Yoga and travels worldwide to teach at seminars.
Bhavini Kalan is a Vinyasa Yoga teacher and has trained with all the top masters in Mysore, including Pattabhi Jois and Srivatsa Ramaswami.
Hikaru Hashimoto founded the Shinto Yoga style in Japan and is a top Hatha Yoga master. His association currently runs more than 700 classes throughout Japan.

SESSION 8
Educational Module: "Patanjali's Classical Yoga Philosophy"
Narrative adapted from The Yoga Tradition by Georg Feuerstein [1]

Questions for Film Segment:
1) What do you think was one of Swami Satchidananda's most important yoga-related teachings for the West?
2) Within this film, what are some examples of how yoga might have positive effects beyond promoting personal health and wellness?
3) Which speaker, other than Swami Satchidananda, affected you most positively or most negatively, and why?

Film [8]: "Living Yoga: The Life and Teachings of Swami Satchidananda"
Swami Satchidananda, a disciple of the renowned guru Sri Swami Sivananda of Rishikesh, was known for his combination of practical wisdom and spiritual insight. He was invited to America in 1966 by the iconic pop artist Peter Max. During this period of cultural awakening, a small circle of Peter Max's artist friends beseeched the swami to extend what was supposed to be a brief stop in New York City so they could learn from him the secret of finding physical, mental and spiritual health, peace and enlightenment. Three years later, he led some half a million American youth in chanting OM, when he delivered the official opening remarks at the 1969 Woodstock Music and Art Festival, and he became known as "the Woodstock guru." The distinctive teachings he brought with him blend the physical discipline of Yoga, the spiritual philosophy of Vedantic literature and the interfaith ideals he pioneered.

SESSION 9
Educational Module: "Classical Yoga Philosophy: Yama."
Narrative adapted from The Yoga Tradition by Georg Feuerstein [1]

Questions for Film Segment:
1) How do you square yoga's spiritual intent with the notion of a yoga competition?
2) In your opinion, is there justification for the patenting of yoga exercises?
3) What happens to the traditional importance of the teacher-student relationship when yoga classes can sometimes swell to dozens, even hundreds, of students?

Film [9]: Yoga Inc. (First 45 mins)
This film is Australian director John Philp's feature-length documentary on the marketing of yoga in the United States, narrated with an engaging mix of seriousness and humor. Wanting to make a point about the unabashed commercialism in American yoga, Philp found Bikram Choudhury, with his enormously profitable and regulated franchise, to be a perfect example. Interviewing various yoga teachers, Philp suggests that yoga is in a process of external accommodation and adaptation, while holding firm to its spiritual core.

SESSION 10
Educational Module: "Classical Yoga Philosophy: Niyama"
Narrative adapted from The Yoga Tradition by Georg Feuerstein [1].

Questions for Film Segment:
1) According to this film, how may yoga play a key role in coping with physical & psychological pain?
2) Discuss our modern culture's conflicted relationship to food, and ways that yoga may help to re-establish a healthier approach to eating.
3) One yoga teacher says, 'yoga has been the foundation to my relationship.' What do you think she means by this?
4) Which speaker do you think illustrates most clearly the idea that yoga practice helps create a connection, not just with our bodies and our breath but also with our communities and the environment?

Film [10]: Yoga and Me (Portions 3 through 11)
This independent documentary features anecdotes from the lives of several yoga teachers. Yoga philosophy can be hard to digest from texts, but comes alive when teachers tell stories from their own experiences.

SESSION 11
Educational Module: "Classical Yoga Philosophy: Asana"
Narrative adapted from The Yoga Tradition by Georg Feuerstein [1].

Questions for Film Segment:
1) After watching this film, do you think yoga can function as an effective form of community participation to guide people through devastating events? Why or why not?
2) In this film, which individual story about the effects of Ashtanga practice affected you most strongly? Why?
3) How did the celebrity yoga practitioners featured in this film affect your reaction to the film?

Film [11]: Ashtanga, NY - A Yoga Documentary
Ashtanga, NY was originally intended as a concert film celebrating the Ashtanga style of yoga, now wildly popular in the United States. It follows the aging yoga guru Sri K. Pattabhi Jois as he gives his final North American workshop in New York City in September 2001. Devotees were arriving from all over the world to join this rare opportunity to practice with the 86 year-old Jois. The 9/11 terrorist attacks took place in the midst of this celebration, and dramatically changed the meaning and purpose of the yoga gathering.

SESSION 12
Educational Module: Classical Yoga Philosophy: "Pranayama"
Narrative adapted from: The Science of Pranayama by Swami Sivananda [12]

Questions for Film Segment:
1) What are the three main elements of Anusara Yoga?
2) The poses and breathing exercises in Anusara yoga are considered to be "heart-oriented," meaning that they are expressed from the "inside out." Instead of trying to control the body and mind from the outside, the yoga exercises are said to originate from a "deep creative and devotional feeling" inside. Explain how this philosophy may have both pros and cons for a beginning yoga student.
3) Which speaker in this film made the deepest impression on you? Why?

Film [13]: Anusara Yoga: The Heart of Transformation
Inspired by the stories of transformation they were hearing from Anusara yoga students, film makers Saraswati and Kate Clere decided to look into what makes this system of yoga so effective. Features interviews with founder John Friend, and Anusara teachers and practitioners from around the world.

SESSION 13
Educational Module: Classical Yoga Philosophy: "Pratyahara"
Narrative adapted from The Yoga System by Swami Krishnananda [14]

Questions for Film Segment:
1) What was Sri Aurobindo's main critique of the ascetic traditions within yoga?
2) Explain two key concepts unique to Integral Yoga philosophy, such as the Supermind or Conscious Evolution.
3) How does Integral Yoga come to influence Esalen and other institutions in the human potential movement?

Film [15]: Integral Consciousness: Sri Aurobindo's Yoga & how Chaudhuri brought it to the West
This film outlines Integral Yoga, the spiritual philosophy developed by Sri Aurobindo--one of the great sages of the 20th Century-- and how this yoga was brought to the West by Dr. Haridas Chaudhuri. The film includes a history of Integral Yoga, from Sri Aurobindo's origins as a revolutionary leader determined to free India from British rule, to his founding an ashram at Pondicherry and the beginnings of Auroville. The film presents pivotal figures in America including Alan Watts and Michael Murphy, and recounts the birth of the California Institute of Integral Studies, the Esalen Institute, and the Cultural Integration Fellowship.

SESSION 14
Educational Module: Patanjali's Yoga Philosophy: "Dharana"
Narrative adapted from The Yoga System by Swami Krishnananda [14]

Questions for Film Segment :
1) In what ways did the yogi in this film fit or not fit your own pre-existing notions of a yogi?
2) What effect do you think yoga demonstrations such as this Fire Ritual have upon most viewers, and do you this is positive, negative or neutral?
3) How many different aspects of classical yoga did you observe this yogi embody over the course of the film?

Film [16]: The Fire Yogi
This film portrays an unusual Fire Ritual performed by an Indian yogi, using a unique breathing and meditative technique to "get into union with Fire."

SESSION 15
Educational Module: Patanjali's Yoga Philosophy: Dhyana
Narrative adapted from The Yoga Tradition by Georg Feuerstein [1].

Questions for Film Segment A:
1) After watching this film, what do you think was the key element that transformed Richard Alpert, Harvard professor of psychology, into Ram Dass, Hindu holy man and yogi?
2) What position does Ram Dass, in contrast to Richard Alpert, seem to take re: LSD as a path to enlightenment?
3) Name one positive and one negative aspect of having figures like Ram Dass introduce yoga to the West.

Film Segment A [17]: Evolution of a Yogi (28 min)
This film captures the uniqueness of a particular moment in American life during the 1960s, offering a rare window into the metamorphosis of Richard Alpert, Harvard psychologist, into Ram Dass, American mystic.

Questions for Film Segment B:
1) How does this film address the relevance of yoga practices (such as Transcendental Meditation) in addressing societal problems such as poverty?
2) In the time period portrayed in this film, what role do you think young people played in popularizing a yoga practice such as Transcendental Meditation? Would young people play a similar role today?

Film Segment B [18] : Maharishi Mahesh Yogi (First 25 minutes, up to the nature montage)
This is the only documentary style film ever made with MAHARISHI MAHESH YOGI, talking about Transcendental Meditation. He is seen throughout the film, interacting with individuals of the 60's.

SESSION 16
Educational Module: "Classical Yoga Philosophy: Samadhi
Narrative adapted from The Yoga Tradition by Georg Feuerstein [1].

Questions for Film Segment:
1) Describe your reaction to the initiation rites of 14-year-old Santosh Giri into the ancient Naga Baba sect: did you feel his welfare would be safeguarded? Did you feel he was responding to a calling?
2) As Shiv Raj Giri and his band of disciples wander over north India, what do they observe about the larger society around them? What does this seem to portend about their survival in modern India?
3) What do you think the yogis in this film meant by the following statements:
   "Even after becoming a yogi, there is no peace."
   "You can't become a Yogi by wearing some robes, you can't become a Yogi by just wanting to be one."

Film [19]: Naked In Ashes (selected 45 minutes)
Paula Fouce's illuminating documentary takes the viewer into the profound spiritual world of India's yogis. In an increasingly materialistic and chaotic world, the yogis offer a dramatically alternative way of life.

REFERENCES

1. Feuerstein G. The yoga tradition: its history, literature, philosophy, and practice. Prescott, AZ.: Hohm Press; 2001.

2. Desai G. Yoga Unveiled: The Evolution and Essence of a Spiritual Tradition. YogaUnveiled.Com; 2004.

3. Fouce P. Origins of Yoga: Quest for the Spiritual. Paradise Film Works; 2005.

4. Lake J, Spiegel D, editors. Prathikanti, S. Ayurveda and mental health. In Complementary and alternative treatments in mental health care. 1st ed. Washington, DC: American Psychiatric Pub; 2007.

5. Hebner J, Osborn D. Kumbha Mela: the world's largest act of faith. La Jolla, CA: Ganesh Editions; 1990.

6. Benazzo M, Day N. Shortcut to Nirvana. Mela Fims LCC; 2004.

7. Braeley J. The Yoga Gurus. Empty Mind Films; 2009.

8. Kumar S. Living Yoga: The Life and Teachings of Swami Satchidananda. Stories to Remember; 2008.

9. Philip J. Yoga Inc. Bad Dog Tales; 2007.

10. Baldwin R. Yoga and Me. Robbie Baldwin; 2008.

11. Laskow C, Wigmore M. Ashtanga, NY. Americas in Transition; 2003.

12. Sivananda. The science of Pranayama. Himalayas, India: Divine Life Society; 1993.

13. Clere S, Clere K. Anusara Yoga: The Heart of Transformation. Yoga Kula; 2010.

14. Swami Krishnananda. THE YOGA SYSTEM [Internet]. Rishikesh, INDIA: Divine Life Society; 1981. Available: www.swami-krishnananda.org

15. Kitchell M. Integral Consciousness: Sri Aurobindo's Yoga and how Haridas Chaudhuri brought it to the West. Cultural Integration Fellowship; 2010.

16. Vasan M. The Fire Yogi. 3dMaxMedia, Inc.; 2006.

17. Hartley E. Evolution of a yogi. Hartley Productions.; 2009.

18. Waite A. Maharishi Mahesh Yogi: Sage for a New Generation. Alan Waite Productions; 1968.

19. Fouce P. Naked in Ashes. Paradise Film Works International; 2006.
